# 1,3,4-Thiadiazole-Containing Azo Dyes: Synthesis, Spectroscopic Properties and Molecular Structure

**DOI:** 10.3390/molecules25122822

**Published:** 2020-06-18

**Authors:** Agnieszka Kudelko, Monika Olesiejuk, Marcin Luczynski, Marcin Swiatkowski, Tomasz Sieranski, Rafal Kruszynski

**Affiliations:** 1 Department of Chemical Organic Technology and Petrochemistry, The Silesian University of Technology, Krzywoustego 4, PL-44100 Gliwice, Poland; monika.olesiejuk@polsl.pl (M.O.); marcin.luczynski@polsl.pl (M.L.); 2 Department of X-ray Crystallography and Crystal Chemistry, Institute of General and Ecological Chemistry, Lodz University of Technology, Żeromskiego 116, PL-90924 Łódź, Poland; marcin.swiatkowski@p.lodz.pl (M.S.); tomasz.sieranski@p.lodz.pl (T.S.); rafal.kruszynski@p.lodz.pl (R.K.)

**Keywords:** heterocycles, 2-arylazo-5-aryl-1,3,4-thiadiazoles, azo-coupling reactions, crystal structure

## Abstract

Three series of azo dyes derived from 2-amino-5-aryl-1,3,4-thiadiazoles and aniline, *N,N*-dimethylaniline and phenol were synthesized in high yields by a conventional diazotization-coupling sequence. The chemical structures of the prepared compounds were confirmed by ^1^H-NMR, ^13^C-NMR, IR, UV-Vis spectroscopy, mass spectrometry and elemental analysis. In addition, the X-ray single crystal structure of a representative azo dye was presented. For explicit determination of the influence of a substituent on radiation absorption in UV-Vis range, time-dependent density functional theory calculations were performed.

## 1. Introduction

Thiadiazoles are five-membered heterocyclic arrangements which are rarely found in nature. Nitrogen, sulfur and carbon atoms can be arranged in several different ways in such a ring, which gives rise to several isomers: 1,2,3-thiadiazole, 1,2,4-thiadiazole, 1,2,5-thiadiazole and 1,3,4-thiadiazole [1,2]. Of these four isomers, derivatives of 1,3,4-thiadiazole seem to be the most popular among scientists. Many compounds containing such a scaffold exhibit a broad spectrum of biologic interactions and have been shown to have antifungal [3,4], antimicrobial [5], anti-inflammatory [6] and anticancer activities [7]. As well as other industrial applications, it is worth mentioning their use as viscosity stabilizers in rubber processing [8], additives for the production of lithium battery electrodes [9], dyes [10] and optoelectronic materials [11]. There are also reports of the applications of 1,3,4-thiadiazole derivatives, in particular 2,5-dimercapto-1,3,4-thiadiazoles, as lubricants [12].

Azo dyes, characterized by the presence of azo moiety (N=N) in their structure, are the most essential group of disperse dyes [13]. They have found a broad application mainly in dyestuff industry, but also in food, cosmetics and pharmaceuticals production. Generally azo dyes exhibit excellent coloring properties and offer vivid colors, starting from yellows, through oranges, reds and ending up in blues. One of the subgroups of this family are conjugated 1,3,4-thiadiazoles containing an azo group (N=N) in their structure. Such heterocyclic azo disperse dyes have received attention from the scientific community due to their brightness, clarity and affinity to various fibers [14,15]. The most common methodology to introduce this type of group into a final azo compound is a two-step transformation through appropriate diazonium salts [16]. The latter are usually produced from the reaction of primary aromatic amines with nitrites in the presence of strong mineral acids at low temperatures. Due to the extreme instability of diazonium salts [17], they are used immediately after their formation in coupling reactions with phenols or amines, substituted with electron-donating groups. Other methods used to prepare azo dyes include the condensation of nitro compounds with amines [18], reduction of nitro compounds [19,20], oxidation of amines [21,22] or condensation of nitroso compounds with amines [23]. In contrast to typical aromatic amines, diazotization and subsequent coupling of 1,3,4-thiadiazole-2-amine derivatives is rarely described in the literature [24,25,26], although thiadiazoles contain an important thiazole chromophoric core in their structure. This may be due to the presence of an additional electronegative nitrogen atom, which decreases the basicity of the external amino group and reduces its reactivity.

In continuation of our study on the synthesis and versatile applications of conjugated 1,3,4-thiadiazole derivatives [27,28,29], we attempted to synthesize and characterize the structural features of a new series of 2-phenylazo-1,3,4-thiadiazoles, functionalized with aryl substituents directly on the heterocyclic ring. Such systems combining the 1,3,4-thiadiazole ring with other aromatic compounds through an azo linker may find potential industrial applications, not only as classical synthetic dyes and pigments, laser dyes and monomers for the production of OLEDs, but also in medicine and agriculture due to the presence of a toxophoric N–C–S moiety [30].

## 2. Results and Discussion

### 2.1. Synthesis

**Scheme 1:**
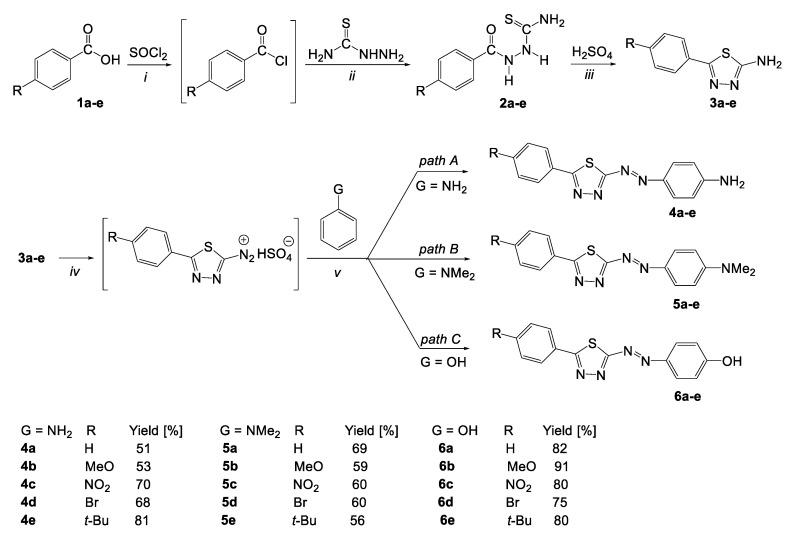
Preparation of 2-amino-1,3,4-thiadiazole precursors (**3a**–**e**) and synthesis of 2-arylazo-5-aryl-1,3,4-thiadiazole dyes (**4a**–**e**, **5a**–**e**, **6a**–**e**). Reaction conditions: (i) toluene, reflux, 5–15 h; (ii) toluene, NaHCO_3_, H_2_O, rt, 24 h; (iii) conc. H_2_SO_4_, 24 h and then aqueous NH_3_; (iv) NaNO_2_, conc. H_2_SO_4_, AcOH/EtCOOH, 0–5 °C; (v) H_2_O, 0–5 °C and then Na_2_CO_3_.

The starting reagents in the synthesis of 1,3,4-thiadiazole-containing azo dyes were commercially available aromatic carboxylic acids substituted at position 4 with electron-donating or withdrawing groups (**1a**–**e**, Scheme 1). They were heated with thionyl chloride SOCl_2_ and the resulting crude acid chlorides were transformed into derivatives of 2-benzoylhydrazinecarbothioamide (**2a**–**e**) in a two-phase water–toluene solvent system in the presence of base (NaHCO_3_). The resulting acyclic intermediates underwent cyclization in concentrated sulfuric acid to give the desired 2-amino-5-aryl-1,3,4-thiadiazoles (**3a**–**e**). The next stages in the few-step reaction sequence were the diazotization of 2-amino-1,3,4-thiadiazole derivatives (**3a**–**e**) and the subsequent coupling of the diazonium salts formed from aniline, *N,N*-dimethylaniline and phenol (Scheme 1). Diazotization involved the reaction of a primary heteroaromatic amine (**3a**–**e**) with a nitrosating agent (nitrosyl cation) generated in situ from sodium nitrite and concentrated sulfuric acid at low temperatures. Reactions were performed in a mixture of glacial acetic acid and propionic acid in order to improve the solubility of the starting thiadiazoles. An excess of nitrous acid in the post-reaction mixture was detected with potassium iodide–starch study and eliminated by adding urea. The resulting colored diazonium salts were then used directly without purification in the coupling sequence with aromatic amines: aniline (G=NH_2_) and *N,N*-dimethylaniline (G=NMe_2_) and with phenol (G=OH). Due to the limited stability of diazonium salts, the transformations were carried out at low temperatures (0–5 °C) and the final products were precipitated from solution by adding base (Na_2_CO_3_) after reagents were combined. The reactions resulted in the formation of three series of 2-arylazo-5-aryl-1,3,4-thiadiazole dyes in various yields (**4a**–**e**, **5a**–**e**, **6a**–**e**, Scheme 1). The highest yields were obtained from the transformations involving phenol (**6a**–**e**, 75–91%, Scheme 1), while the reactions using *N,N*-dimethylaniline (**5a**–**e**, 56–69%, Scheme 1) and aniline (**4a**–**e**, 51–81%, Scheme 1) produced lower yields. Such situation may be caused by partial deactivation of anilines in acidic media. Generally, 1,3,4-thiadiazole-containing azo dyes are high-melting solids (melting point range 169–293 °C) and are insoluble in both water and hydrocarbons (hexane, toluene). They are highly soluble in DMSO, acetone and methanol. It is worth noting that among the synthesized derivatives, 12 are new compounds not yet described in the literature.

### 2.2. Spectral Characterization

The structures of the final diazotization products of 2-amino-5-aryl-1,3,4-thiadiazole and their subsequent coupling with aniline, *N,N*-dimethylaniline and phenol were confirmed by typical spectroscopic methods (^1^H-NMR, ^13^C-NMR, UV-Vis, FT-IR and HRMS). In the ^1^H-NMR spectra of 2-arylazo-5-aryl-1,3,4-thiadiazoles (**4a**–**e**, **5a**–**e**, **6a**–**e**), the characteristic signals become from protons located in the benzene rings associated with the 1,3,4-thiadiazole core and appeared in the range between 6.74–8.40 ppm. Signals of the characteristic amino group (7.06–7.18 ppm), *N,N*-dimethylamino group (~3.18 ppm), *t*-butyl (~1.33 ppm) and methoxy (~3.86 ppm) were also observed. In the ^13^C-NMR spectra the characteristic peaks from 1,3,4-thiadiazole carbon atoms C-2 and C-5 were observed at 164–166 ppm and 178–181 ppm, respectively. Signals due to methoxy group (~55.5 ppm), *t*-butyl group (30.8–34.8 ppm) and *N,N*-dimethylamino group (40.0–40.2 ppm) were observed upfield in the spectra. The formation of azo dyes **4a**–**e**, **5a**–**e**, **6a**–**e** was further shown by HRMS and elemental analyses.

The IR spectra of all prepared dyes were recorded from 4000 and 650 cm^−1^. For the dyes **4a**–**e**, two bands, respectively at 3400–3310 cm^−1^ and 3201–3190 cm^−1^, were visible, which were attributed to the presence of a free amino group. Such bands were not observed in the case of derivatives **5a**–**e**, representing a group of tertiary amines. In series **6a**–**e**, a broad band appeared from 3500–3100 cm^−1^, which confirms the presence of a hydroxyl group. All dyes showed a weak band at 1505–1525 cm^−1^ for an azo group (N=N).

**Figure 1:**
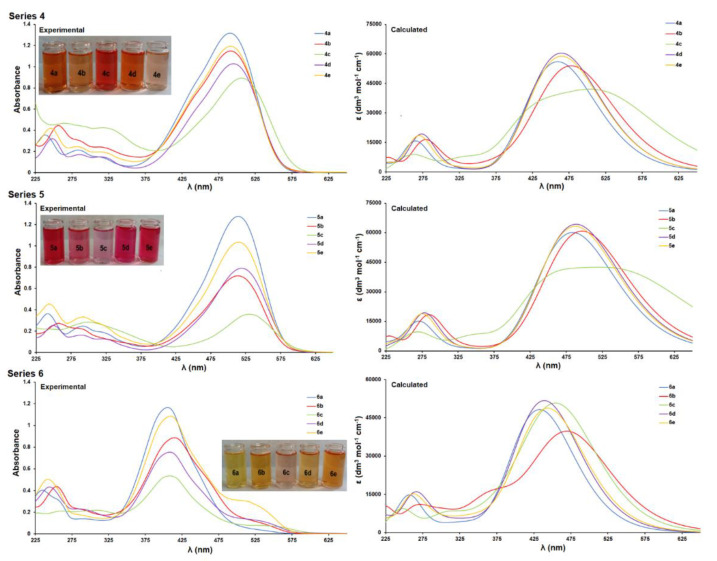
UV-Vis absorption spectra of the studied azo dyes in MeOH at a concentration of 4.0 × 10^−5^-mol/L at room temperature: (**Series 4**) Absorption spectra of azo dyes **4a**–**e** containing 4-aminophenylazo group; (**Series 5**) absorption spectra of azo dyes **5a**–**e** containing 4-(*N,N*-dimethylamino)phenylazo group; (**Series 6**) absorption spectra of azo dyes **6a**–**e** containing 4-hydroxyphenylazo group. Respective calculated spectra are shown as well.

The UV-Vis spectra of three series of 2-arylazo-5-aryl-1,3,4-thiadiazole dyes (**4a**–**e**, **5a**–**e**, **6a**–**e**) measured in MeOH exhibit several (four in most cases) absorption maxima (Figure 1, Table 1). The same is observed for calculated spectra (Figure 1, Table 1) which are in good comparison with those measured. All maxima result from multiple different transitions and include both n→π* and π→π* transitions. For series **5** and **6** with compounds possessing a t-butyl group, additionally the σ→π* transitions are observed. Due to complexity of electronic transition creating each absorption maximum, there is no one evident dependence between the substituents and absorption maximum position/absorption coefficient (Figure S1). Nevertheless, some dependences exist within groups with one kind of R or G substituent. The change of the G substituent causes the red shift of the most intense absorption maximum (appearing in the visible regions of 490–507 nm for **4a**–**e**, 515–530 nm for **5a**–**e** and 405–415 nm for **6a**–**e**) in order of G substituents: OH, NH_2_, NMe_2_. This sequence is in agreement with electron donating properties of the studied substituents. These red shifts are well reproduced in calculated spectra (Table 1). For the compounds with amino substituents (G=NH_2_, NMe_2_) this maximum is the most red-shifting for a compound possessing a nitro group (Table 1). For the compounds with hydroxy group (G=OH) the red shift forced by the presence of a nitro group (**6c**) is between some other red shifted compounds, and it is smaller than that shift observed for the compound **6b**. This effect is also reflected in the calculated spectra (Table 1). The simultaneous presence of a hydroxy group (G=OH) and methoxy one (R=MeO) in **6b** is an explanation of this phenomenon. For each series, the methoxy moiety induces red-shift effect (toward most of the compounds), but for amino substituents G=NH_2_, NMe_2_ it was smaller than for their hydroxy counterpart G=OH (influence of this group could only be noticed for calculated spectra). Increase of the red shift for stronger electron donating EDG substituents (G=NMe_2_ and NH_2_) is larger than caused by methoxy moiety and overrule strong electron withdrawing properties of nitro substituent. In case of the weaker EDG substituent (G=OH) with electron donating properties close to the methoxy substituent, the electron withdrawing properties of nitro substituent dominate at some point, and cause above described difference in red shifts. For each compound, the most intense (global) maximum is attributed to HOMO–LUMO (H→L) transitions (Table 1, Supplementary Materials, Figures S2–S4). For compounds possessing the nitro substituent these experimentally observed absorption maxima result also from other than H→L transitions, possessing relatively large oscillator strengths (Table 1, Figures S2–S4). In all cases, these transitions do not engage the antibonding orbitals of the nitro group (Table 1). For the second most intense absorption maximum (experimentally observed at lower wavelengths), the changes of its shift between the corresponding compounds (e.g., **4a** vs. **5a** vs. **6a**) are not as high as those observed for the most intense maxima. Those maxima appear in the ultraviolet regions of 237–267 nm for **4a**–**e**, 243–256 nm for **5a**–**e** and 243–260 nm for **6a**–**e** and they results from multiple π→ π* and n→ π* transitions involving antibonding orbitals of all substituents (i.e., NH_2_, NMe_2_, OH, MeO, NO_2_ and Br, Table 1). The experimental and calculated spectra show some minor differences. For compounds with amino groups (G=NH_2_, NMe_2_), the calculated spectra do not contain absorption maxima observed experimentally in the range of 284–299 nm. This may result from the interaction of compound molecules with the solvent (i.e., formation of specific hydrogen bonds) defectively reproduced in continuous model of solvation.

### 2.3. Molecular Structure

**Figure 2:**
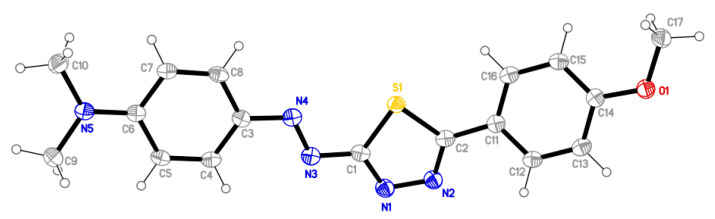
Molecular structure of compound **5b** plotted with 50% probability of displacement ellipsoids. Hydrogen atoms are drawn as spheres with arbitrary radii.

The structure of one of the obtained azo dyes was confirmed by X-ray crystal structure analysis (CCDC1946289). All atoms of the studied 2-[4-(*N,N*-dimethylamino)phenylazo]-5-(4-methoxyphenyl)-1,3,4-thiadiazole (**5b**) occupied general positions (Figure 2).

The five- and six- membered rings were practically planar (the largest deviating atom C2, C7, C14 stuck out 0.0060(8), 0.0079(9), 0.0073(9) Å from the weighted least squares plane containing the respective atom), but the whole molecule is considerably bent. The benzene rings containing C3 and C11 atoms were inclined at 10.11(7) and 24.90(6)° to the central 1,3,4-thiadiazole ring, respectively and mutually at 26.85(6)°. The *N,N*-dimethylamino group was inclined at 11.53(14)° to the neighboring benzene ring, which is a larger value than is typically observed for similar systems (4.6%–5.8°) [30]. However, it still falls within the range of 2.3%–18.8° (the angle values of median population). The C–S bonds (Table 2) were shorter than typical single C–S bonds (i.e., a mean value of 1.819 Å for a bond between an sp^3^ carbon atom and a divalent sulfur) and similar to the values observed for slightly delocalized systems containing sp^2^ carbon atoms (i.e., a mean value of 1.741 Å) [31]. A similar effect exists for an N–N bond (a formal bond length of 1.454 Å) and opposite effects were observed for slightly longer C=N bonds (Table 2) and double C=N bonds (i.e., a mean value of 1.279 Å [32]). Combined, these effects demonstrate small, but observable delocalization of electron density within the 1,3,4-thiadiazole ring. The azo N=N bond (Table 2) shows a transitional character between a well-localized and a completely delocalized system (mean values of 1.245 and 1.304 Å) [33]. This, along with the shortening of the neighboring C–N bonds, also demonstrates the considerable degree of delocalization of electron density within the C–N=N–C moiety. Due to the absence of classical hydrogen bond donors, the studied compounds are connected only by weak C–H**•••**A non-classic hydrogen bonds (where A denotes the following acceptors: N, O, S and π electrons) [34] and π**•••**π stacking interactions (Table 3, Table 4) [35].

**Figure 3:**
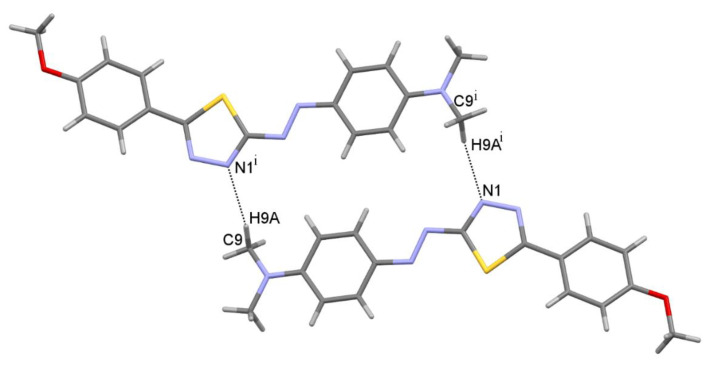
Supramolecular dimers forming a R_2_^2^(22) motif of a unitary graph set. Symmetry codes as in Table 3. Non-classic hydrogen bonds are indicated by dotted lines.

**Figure 4:**
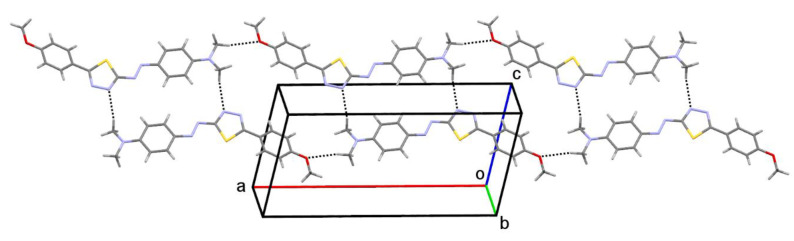
Part of molecular packing showing a supramolecular ribbon extending along the crystallographic axis. Hydrogen bonds indicated by dotted lines.

The unitary graph set is composed of R_2_^2^(22)C(17)S(5)C(2) motifs of the lowest degree. The ring motif assembles the molecules into supramolecular dimers (Figure 3), and the C(17) chain motif extends these dimers to a supramolecular ribbon extending along the crystallographic axis (Figure 4). This ribbon is constructed from alternating R_2_^2^(22)R_4_^4^(26) ring motifs of a binary graph set of the lowest degree. The neighboring ribbons are interconnected by C–H**•••**π and π**•••**π interactions (Table 3, Table 4) and form a three-dimensional network assembled by non-covalent interactions.

## 3. Experimental

### 3.1. General Information

All reagents were purchased from commercial sources and used without further purification. Melting points were measured on a Stuart SMP3 melting point apparatus. The ^1^H-NMR and ^13^C-NMR spectra were recorded on an Agilent 400-NMR spectrometer in DMSO-*d*_6_ solution using TMS as the internal standard. FT-IR spectra were recorded between 4000 and 650 cm^−1^ using a FT-IR Nicolet 6700 apparatus with a Smart iTR accessory. UV-Vis spectra were recorded at room temperature in MeOH solutions (c = 4.0 × 10^−5^ mol/L) on a Jasco V-660 spectrophotometer. High-resolution mass spectra were recorded on a Waters ACQUITY UPLC/Xevo G2QT instrument. Thin-layer chromatography was performed on silica gel 60 F_254_ (Merck) TLC plates using CHCl_3_/EtOAc (5:1 *v*/*v*) as the mobile phase. Elemental analyses were performed with a VarioEL analyzer. The excited states of the obtained compounds were calculated using TD–DFT method. All calculations (including geometry optimization) were performed utilizing Gaussian09 rev. D.01 [36] with B3LYP functional and employing 6.31 g++(2d, 2p) basis set. The geometry of all compounds was optimized and during this calculation D3 version of Grimme’s dispersion with Becke–Johnson damping [37] was used. In both, the optimization process and TD–DFT calculations, the polarizable continuum model [38] was used to include overall solvation effects (with methanol selected as a solvent). The assignment of the calculated excited states to the observed experimental maxima was based on the comparison of excitation energies and the oscillator strengths/intensities of the corresponding maxima. The analysis of the character of respective orbital excitations was based on orbital contour plots.

### 3.2. Synthesis and Characterization

#### 3.2.1. General Procedure for the Synthesis of 2-Benzoylhydrazinecarbothioamide Derivatives (**2a**–**e**)

A carboxylic acid (**1a**–**e**, 0.10 mol) and thionyl chloride SOCl_2_ (22 mL, 0.30 mol) were refluxed in dry toluene (10 mL) until the acid was fully consumed (TLC; 5–15 h). After cooling, the mixture was concentrated on a rotary evaporator, washed with additional dry toluene (10 mL) and concentrated again. The crude acid chloride was then dissolved in 50 mL of dry toluene and added dropwise to a mixture of thiosemicarbazide (9.11 g, 0.10 mol), NaHCO_3_ (8.40 g, 0.10 mol) and H_2_O (150 mL). The whole solution was agitated at room temperature overnight. The precipitated solid was filtered off, dried in air and recrystallized from a mixture of EtOH–H_2_O to obtain pure 2-benzoylhydrazinecarbothioamide derivatives (**2a**–**e**).

*2-Benzoylhydrazinecarbothioamide* (**2a**). The product was obtained as a white solid (8.60 g, 44%); mp 197–198 °C (196–198 °C [39]).

*2-(4-Methoxybenzoylhydrazinecarbothioamide* (**2b**). The product was obtained as a white solid (15.32 g, 68%); mp 225–227 °C (226 °C [40]).

*2-(4-Nitrobenzoylhydrazinecarbothioamide* (**2c**). The product was obtained as a yellow solid (18.02 g, 75%); mp 212–214 °C (214 °C [41]).

*2-(4-Bromobenzoylhydrazinecarbothioamide* (**2d**). The product was obtained as a white solid (16.72 g, 75%); mp 216–218 °C. ^1^H-NMR (400 MHz, DMSO-d_6_): δ 7.68–7.72 (m, 3H, Ar: H-3, H-5, NH), 7.82–7.88 (m, 3H, Ar: H-2, H-6, NH), 9.34 (s, 1H, NH), 10.45 (s, 1H, NH); ^13^C-NMR (100 MHz, DMSO-d_6_): δ 125.5, 129.5, 129.9, 131.1, 131.7, 164.9 (C=O), 182.0 (C=S); Anal. Calcd for C_8_H_8_BrN_3_OS: C, 35.05; H, 2.94; N, 15.33. Found: C, 34.95; H, 2.89; N, 15.40.

*2-(4-t-Butylbenzoylhydrazinecarbothioamide* (**2e**). The product was obtained as a beige solid (12.06 g, 48%); mp 210–211 °C. ^1^H-NMR (400 MHz, DMSO-d_6_): δ 1.33 (s, 9H, C(CH_3_)_3_), 7.48 (d, 2H, *J* = 8.4 Hz, Ar: H-3, H-5), 7.52–7.60 (m, 2H, NH_2_), 7.84 (d, 2H, *J* = 8.4 Hz, Ar: H-2, H-6), 9.31 (s, 1H, NH), 10.39 (s, 1H, NH); ^13^C-NMR (100 MHz, DMSO-d_6_): δ 30.8, 34.8, 124.9, 126.1, 128.3, 154.6, 165.6 (C=O), 182.0 (C=S); Anal. Calcd for C_12_H_17_N_3_OS: C, 57.34; H, 6.82; N, 16.72. Found: C, 57.21; H, 6.88; N, 16.63.

#### 3.2.2. General Procedure for the Synthesis of 2-Amino-1,3,4-thiadiazole Derivatives (**3a**–**e**)

Derivatives of 2-benzoylhydrazinecarbothioamide **2a**–**e** (0.05 mol) were dissolved in 50 mL of concentrated H_2_SO_4_ and agitated at room temperature overnight. Then, the mixture was alkalized with 25% ammonia and the precipitated product was filtered off, dried in air and recrystallized from a mixture of EtOH–H_2_O to yield the corresponding 2-amino-1,3,4-thiadiazoles **3a**–**e**.

*2-Amino-5-phenyl-1,3,4-thiadiazole* (**3a**). The product was obtained as a white solid (3.89 g, 45%); mp 232–233 °C (230 °C [42]).

*2-Amino-5-(4-methoxyphenyl)-1,3,4-thiadiazole* (**3b**). The product was obtained as a white solid (3.42 g, 33%); mp 184–185 °C (185–187 °C [43]).

*2-Amino-5-(4-nitrophenyl)-1,3,4-thiadiazole* (**3c**). The product was obtained as a yellow solid (8.33 g, 75%); mp 254–256 °C (250–252 °C [44]).

*2-Amino-5-(4-bromophenyl)-1,3,4-thiadiazole* (**3d**). The product was obtained as a white solid (8.96 g, 70%); mp 226 °C (222–224 °C [41]).

*2-Amino-5-(4-t-butylphenyl)-1,3,4-thiadiazole* (**3e**). The product was obtained as a white solid (4.78 g, 41%); mp 251–253 °C. ^1^H-NMR (400 MHz, DMSO-d_6_): δ 1.29 (s, 9H, C(CH_3_)_3_), 7.35 (s, 2H, NH_2_), 7.48 (d, 2H, *J* = 8.4 Hz, Ar: H-3, H-5), 7.67 (d, 2H, *J* = 8.4 Hz, Ar: H-2, H-6); ^13^C-NMR (100 MHz, DMSO-d_6_): δ 30.8, 34.5, 125.8, 126.0, 128.3, 152.2, 156.3, 168.1; Anal. Calcd for C_12_H_15_N_3_S: C, 61.77; H, 6.48; N, 18.01. Found: C, 61.70; H, 6.40; N, 18.12.

#### 3.2.3. General Procedure for the Synthesis of 2-(4-Aminophenylazo)-5-phenyl-1,3,4-thiadiazole Derivatives (**4a**–**e**)

Sodium nitrite (1.31 g, 0.019 mol) was introduced portion wise into concentrated sulfuric acid (20 mL). The solution was heated to 50 °C in a water bath until complete dissolution and then rapidly cooled in an ice/salt bath to 0 °C. In the meantime, the solution of the appropriate 2-amino-5-aryl-1,3,4-thiadiazole **3a**–**e** (0.015 mol) in glacial acetic acid (30 mL) and propionic acid (15 mL) was prepared and added dropwise to an agitated solution of sodium nitrite in concentrated sulfuric acid at 0–5 °C. Then, the mixture was stirred for 24 h, and excess nitrous acid was decomposed by the addition of urea. The resulting diazonium salt solution was slowly introduced into the mixture of aniline (1.37 mL, 1.39 g, 0.015 mol) in 15 mL of water at 0–5 °C. The colored mixture was stirred at room temperature for the next 24 h and finally neutralized with saturated sodium carbonate solution (75 mL). The solid was filtered off, washed twice with hot water (2 × 25 mL) and dried in air. The crude product (**4a**–**e**) was purified by column chromatography on silica gel (CHCl_3_/EtOAc, 5:1 *v*/*v*).

*2-(4-Aminophenylazo)-5-phenyl-1,3,4-thiadiazole* (**4a**). The product was obtained as a brown reddish solid (2.11 g, 51%); mp 236–238 °C; R_f_ (CHCl_3_/EtOAc, 5:1 *v*/*v*) 0.18. ^1^H-NMR (400 MHz, DMSO-d_6_): δ 6.75 (d, 2H, *J* = 8.8 Hz, 2-Ar: H-3, H-5), 7.13 (s, 2H, NH_2_), 7.57–7.59 (m, 3H, 5-Ar: H-3′, H-4′, H-5′), 7.77 (d, 2H, *J* = 8.8 Hz, 2-Ar: H-2, H-6), 8.04 (d, 2H, *J* = 8.4 Hz, 5-Ar: H-2′, H-6′); ^13^C-NMR (100 MHz, DMSO-d_6_): δ 114.1, 127.5, 127.8, 129.4, 130.1, 131.5, 142.3, 156.6, 165.7, 180.2. IR (ATR) ν: 3311, 1636, 1507 cm^−1^; HRMS calcd for (C_14_H_11_N_5_S + H^+^): 282.0813; found: 282.0818; Anal. Calcd for C_14_H_11_N_5_S: C, 59.77; H, 3.94; N, 24.89. Found: C, 59.83; H, 3.97; N, 24.93.

*2-(4-Aminophenylazo)-5-(4-methoxyphenyl)-1,3,4-thiadiazole* (**4b**). The product was obtained as a brown reddish solid (2.47 g, 53%); mp 269–270 °C; R_f_ (CHCl_3_/EtOAc, 5:1 *v*/*v*) 0.16. ^1^H-NMR (400 MHz, DMSO-d_6_): δ 3.86 (s, 3H, OCH_3_), 6.74 (d, 2H, *J* = 8.8 Hz, 2-Ar: H-3, H-5), 7.06 (s, 2H, NH_2_), 7.11 (d, 2H, *J* = 8.8 Hz, 5-Ar: H-3′, H-5′), 7.75 (d, 2H, *J* = 8.8 Hz, 2-Ar: H-2, H-6), 7.98 (d, 2H, *J* = 8.8 Hz, 5-Ar: H-2′, H-6′); ^13^C-NMR (100 MHz, DMSO-d_6_): δ 55.5, 114.1, 114.8, 122.6, 127.6, 129.2, 142.3, 156.3, 161.8, 165.6, 179.5. IR (ATR) ν: 3422, 3333, 1651, 1508 cm^−1^; HRMS calcd for (C_15_H_13_N_5_SO + H^+^): 312.0919; found: 312.0912; Anal. Calcd for C_15_H_13_N_5_SO: C, 57.86; H, 4.21; N, 22.49. Found: C, 57.73; H, 4.24; N, 22.43.

*2-(4-Aminophenylazo)-5-(4-nitrophenyl)-1,3,4-thiadiazole* (**4c**). The product was obtained as a dark brown solid (3.42 g, 70%); mp 176–178 °C (178–179 °C [24]); R_f_ (CHCl_3_/EtOAc, 5:1 *v*/*v*) 0.23. IR (ATR) ν: 3403, 1637, 1513 cm^−1^; Anal. Calcd for C_14_H_10_N_6_O_2_S: C, 51.53; H, 3.09; N, 25.75. Found: C, 51.47; H, 3.05; N, 25.77.

*2-(4-Aminophenylazo)-5-(4-bromophenyl)-1,3,4-thiadiazole* (**4d**). The product was obtained as a dark violet solid (3.67 g, 68%); mp 261–263 °C; R_f_ (CHCl_3_/EtOAc, 3:1 *v*/*v*) 0.23. ^1^H-NMR (400 MHz DMSO-d_6_): δ 6.75 (d, 2H, *J* = 9.2 Hz, 2-Ar: H-3, H-5), 7.18 (s, 2H, NH_2_), 7.75–7.78 (m, 4H, 2-Ar, 5-Ar: H-2, H-6, H-3′, H-5′), 7.97 (d, 2H, *J* = 8.8 Hz, 5-Ar: H-2′, H-6′); ^13^C-NMR (100 MHz, DMSO-d_6_): δ 114.2, 124.9, 129.2, 129.3, 130.4, 132.4, 142.3, 156.7, 164.6, 180.5. IR (ATR) ν: 3321, 1636, 1507 cm^−1^; HRMS calcd for (C_14_H_10_N_5_SBr+H^+^): 359.9919; found: 359.9914; Anal. Calcd for C_14_H_10_N_5_SBr: C, 46.68; H, 2.80; N, 19.44. Found: C, 46.81; H, 2.79; N, 19.49.

*2-(4-Aminophenylazo)-5-(4-t-butylphenyl)-1,3,4-thiadiazole* (**4e**). The product was obtained as a brown solid (2.43 g, 81%); mp 171–173 °C; R_f_ (CHCl_3_/EtOAc, 5:1 *v*/*v*) 0.27. ^1^H-NMR (400 MHz, DMSO-d_6_): δ 1.33 (s, 9H, C(CH_3_)_3_), 6.74 (d, 2H, *J* = 8.4 Hz, 2-Ar: H-3, H-5), 7.10 (s, 2H, NH_2_), 7.58 (d, 2H, *J* = 8.8 Hz, 5-Ar: H-3′, H-5′), 7.76 (d, 2H, *J* = 8.4 Hz, 2-Ar: H-2, H-6), 7.96 (d, 2H, *J* = 8.8 Hz, 5-Ar: H-2′, H-6′); ^13^C-NMR (100 MHz, DMSO-d_6_): δ 30.8, 34.8, 114.1, 126.2, 127.3, 127.4, 130.2, 142.3, 154.5, 156.5, 165.7, 179.9. IR (ATR) ν: 3325, 1621, 1507 cm^−1^; HRMS calcd for (C_18_H_19_N_5_S+H^+^): 338.1440; found: 338.1438; Anal. Calcd for C_18_H_19_N_5_S: C, 64.07; H, 5.68; N, 20.75. Found: C, 64.21; H, 5.70; N, 20.71.

#### 3.2.4. General procedure for the synthesis of 2-[4-(*N,N*-dimethylamino)phenylazo]-5-phenyl-1,3,4-thiadiazole derivatives (**5a**–**e**)

Sodium nitrite (1.31 g, 0.019 mol) was introduced portion wise into concentrated sulfuric acid (20 mL). The solution was heated to 50 °C in a water bath until complete dissolution and then rapidly cooled in an ice/salt bath to 0 °C. In the meantime, a solution of the appropriate 2-amino-5-aryl-1,3,4-thiadiazole **3a**–**e** (0.015 mol) in glacial acetic acid (30 mL) and propionic acid (15 mL) was prepared and added dropwise to an agitated solution of sodium nitrite in concentrated sulfuric acid at 0–5 °C. Then, the mixture was stirred for 24 h and excess nitrous acid was decomposed by the addition of urea. The resulting diazonium salt solution was slowly introduced into the mixture of N,N-dimethylaniline (1.90 mL, 1.82 g, 0.015 mol) in 15 mL of water at 0–5 °C. The colored mixture was stirred at room temperature for the next 24 h and finally neutralized with saturated sodium carbonate solution (75 mL). The solid was filtered off, washed twice with hot water (2 × 25 mL) and dried in air. The crude product (**5a**–**e**) was purified by column chromatography on silica gel (CHCl_3_/EtOAc, 5:1 *v*/*v*).

*2-[4-(N,N-Dimethylamino)phenylazo]-5-phenyl-1,3,4-thiadiazole* (**5a**). The product was obtained as a brown solid (3.20 g, 69%); mp 194–195 °C (191 °C [26]); R_f_ (CHCl_3_/EtOAc, 5:1 *v*/*v*) 0.32. IR (ATR) ν: 1603, 1525 cm^−1^; Anal. Calcd for C_16_H_15_N_5_S: C, 62.11; H, 4.89; N, 22.64. Found: C, 62.08; H, 4.86; N, 22.67.

*2-[4-(N,N-Dimethylamino)phenylazo]-5-(4-methoxyphenyl)-1,3,4-thiadiazole* (**5b**). The product was obtained as a dark red solid (3.00 g, 59%); mp 219–221 °C; R_f_ (CHCl_3_/EtOAc, 5:1 *v*/*v*) 0.27. ^1^H-NMR (400 MHz, DMSO-d_6_): δ 3.17 (s, 6H, N(CH_3_)_2_), 3.86 (s, 3H, OCH_3_), 6.92 (d, 2H, *J* = 9.2 Hz, 2-Ar: H-3, H-5), 7.12 (d, 2H, *J* = 8.8 Hz, 5-Ar: H-3′, H-5′), 7.85 (d, 2H, *J* = 9.2 Hz, 2-Ar: H-2, H-6), 7.99 (d, 2H, *J* = 8.8 Hz, 5-Ar: H-2′, H-6′); ^13^C-NMR (100 MHz, DMSO-d_6_): δ 40.2, 55.5, 112.2, 114.8, 122.5, 126.0, 129.2, 129.4, 142.0, 154.6, 164.8, 179.4. IR (ATR) ν: 1603, 1516 cm^−1^; HRMS calcd for (C_17_H_17_N_5_SO+H^+^): 340.1232; found: 340.1233; Anal. Calcd for C_17_H_17_N_5_SO: C, 60.16; H, 5.05; N, 20.63. Found: C, 60.27; H, 5.03; N, 20.61.

*2-[4-(N,N-Dimethylamino)phenylazo]-5-(4-nitrophenyl)-1,3,4-thiadiazole* (**5c**). The product was obtained as a brown solid (3.19 g, 60%); mp 169–171 °C; R_f_ (CHCl_3_/EtOAc, 5:1 *v*/*v*) 0.38. ^1^H-NMR (400 MHz, DMSO-d_6_): δ 3.18 (s, 6H, N(CH_3_)_2_), 7.04 (d, 2H, *J* = 8.8 Hz, 2-Ar: H-3, H-5), 7.96 (d, 2H, *J* = 9.2 Hz, 5-Ar: H-3′, H-5′), 8.37–8.40 (m, 4H, 2-Ar, 5-Ar: H-2, H-6, H-2′, H-6′); ^13^C-NMR (100 MHz, DMSO-d_6_): δ 40.0, 112.3, 124.4, 124.5, 126.9, 129.1, 130.1, 142.1, 155.5, 164.8, 179.8. IR (ATR) ν: 1597, 1516 cm^−1^; HRMS calcd for (C_16_H_14_N_6_SO_2_ + H^+^): 355.0977; found: 355.0979; Anal. Calcd for C_16_H_14_N_6_SO_2_: C, 54.23; H, 3.98; N, 23.71. Found: C, 54.40; H, 3.99; N, 23.76.

*5-(4-Bromophenyl)-2-[4-(N,N-dimethylamino)phenylazo]-1,3,4-thiadiazole* (**5d**). The product was obtained as a dark violet solid (3.49 g, 60%); mp 201–203 °C; R_f_ (CHCl_3_/EtOAc, 5:1 *v*/*v*) 0.40. ^1^H-NMR (400 MHz, DMSO-d_6_): δ 3.18 (s, 6H, N(CH_3_)_2_), 6.91 (d, 2H, *J* = 9.2 Hz, 2-Ar: H-3, H-5), 7.77 (d, 2H, *J* = 8.4 Hz, 5-Ar: H-3′, H-5′), 7.84 (d, 2H, *J* = 9.2 Hz, 2-Ar: H-2, H-6), 7.97 (d, 2H, *J* = 8.4 Hz, 5-Ar: H-2′, H-6′); ^13^C-NMR (100 MHz, DMSO-d_6_): δ 40.0, 112.3, 124.9, 128.8, 129.2, 129.3, 132.4, 142.1, 154.8, 164.7, 180.5. IR (ATR) ν: 1597, 1522 cm^−1^; HRMS calcd for (C_16_H_14_N_5_SBr+H^+^): 388.0232; found: 388.0238; Anal. Calcd for C_16_H_14_N_5_SBr: C, 49.49; H, 3.63; N, 18.04. Found: C, 49.37; H, 3.66; N, 18.09.

*5-(4-t-Butylphenyl)-2-[4-(N,N-dimethylamino)phenylazo]-1,3,4-thiadiazole* (**5e**). The product was obtained as a dark violet solid (3.07 g, 56%); mp 225–227 °C; R_f_ (CHCl_3_/EtOAc, 5:1 *v*/*v*) 0.39. ^1^H-NMR (400 MHz, DMSO-d_6_): δ 1.33 (s, 9H, C(CH_3_)_3_), 3.18 (s, 6H, N(CH_3_)_2_), 6.93 (d, 2H, *J* = 9.2 Hz, 2-Ar: H-3, H-5), 7.59 (d, 2H, *J* = 8.4 Hz, 5-Ar: H-3′, H-5′), 7.86 (d, 2H, *J* = 9.2 Hz, 2-Ar: H-2, H-6), 7.97 (d, 2H, *J* = 8.4 Hz, 5-Ar: H-2′, H-6′); ^13^C-NMR (100 MHz, DMSO-d_6_): δ 30.8, 34.8, 40.1, 112.3, 126.2, 127.4, 127.6, 142.1, 153.4, 154.5, 154.7, 165.8, 179.8. IR (ATR) ν: 1604, 1524 cm^−1^; Anal. Calcd for C_20_H_23_N_5_S: HRMS calcd for (C_20_H_23_N_5_S+H^+^): 366.1752; found: 366.1754; Anal. Calcd for C_20_H_23_N_5_S: C, 65.72; H, 6.34; N, 19.16. Found: C, 65.80; H, 6.33; N, 19.18.

#### 3.2.5. General Procedure for the Synthesis of 2-(4-Hydroxyphenylazo)-5-phenyl-1,3,4-thiadiazole Derivatives (**6a**–**e**)

Sodium nitrite (1.31 g, 0.019 mol) was introduced portion wise into concentrated sulfuric acid (20 mL). The solution was heated to 50 °C in a water bath until complete dissolution and then rapidly cooled in an ice/salt bath to 0 °C. In the meantime, a solution of the appropriate 2-amino-5-aryl-1,3,4-thiadiazole **3a**–**e** (0.015 mol) in glacial acetic acid (30 mL) and propionic acid (15 mL) was prepared and added dropwise to an agitated solution of sodium nitrite in concentrated sulfuric acid at 0–5 °C. Then, the mixture was stirred for 24 h and excess nitrous acid was decomposed by the addition of urea. The resulting diazonium salt solution was slowly introduced into the mixture of phenol (1.41 g, 0.015 mol) in 15 mL of water at 0–5 °C. The colored mixture was stirred at room temperature for 24 h and finally neutralized with saturated sodium carbonate solution (75 mL). The solid was filtered off, washed twice with hot water (2 × 25 mL) and dried in air. The crude product (**6a**–**e**) was purified by column chromatography on silica gel (CHCl_3_/EtOAc, 5:1 *v*/*v*).

*2-(4-Hydroxyphenylazo)-5-phenyl-1,3,4-thiadiazole* (**6a**). The product was obtained as a red solid (3.47 g, 82%); mp 200–202 °C (196–198 °C [25]); R_f_ (CHCl_3_/EtOAc, 5:1 *v*/*v*) 0.25. IR (ATR) ν: 3060, 1608, 1512 cm^−1^; Anal. Calcd for C_14_H_10_N_4_OS: C, 59.56; H, 3.57; N, 19.85. Found: C, 59.50; H, 3.52; N, 19.87.

*2-(4-Hydroxyphenylazo)-5-(4-methoxyphenyl)-1,3,4-thiadiazole* (**6b**). The product was obtained as an orange solid (4.26 g, 91%); mp 271–273 °C; R_f_ (CHCl_3_/EtOAc, 5:1 *v*/*v*) 0.23. ^1^H-NMR (400 MHz, DMSO-d_6_): δ 3.86 (s, 3H, OCH_3_), 7.02 (d, 2H, *J* = 9.2 Hz, 2-Ar: H-3, H-5), 7.13 (d, 2H, *J* = 8.8 Hz, 5-Ar: H-3′, H-5′), 7.92 (d, 2H, *J* = 9.2 Hz, 2-Ar: H-2, H-6), 8.03 (d, 2H, *J* = 8.8 Hz, 5-Ar: H-2′, H-6′); ^13^C-NMR (100 MHz, DMSO-d_6_): δ 55.5, 114.9, 116.7, 122.1, 126.8, 129.6, 144.7, 162.2, 164.1, 167.3, 178.4. IR (ATR) ν: 3099, 1601, 1518 cm^−1^; HRMS calcd for (C_15_H_12_N_4_SO_2_+H^+^): 313.0759; found: 313.0755; Anal. Calcd for C_15_H_12_N_4_SO_2_: C, 57.68; H, 3.87; N, 17.94. Found: C, 57.59; H, 3.85; N, 17.99.

*2-(4-Hydroxyphenylazo)-5-(4-nitrophenyl)-1,3,4-thiadiazole* (**6c**). The product was obtained as a dark brown solid (3.92 g, 80%); mp 258–260 °C; R_f_ (CHCl_3_/EtOAc, 5:1 *v*/*v*) 0.35. ^1^H-NMR (400 MHz, DMSO-d_6_): δ 6.97 (d, 2H, *J* = 8.8 Hz, 2-Ar: H-3, H-5), 7.92 (d, 2H, *J* = 8.8 Hz, 5-Ar: H-3′, H-5′), 8.28–8.40 (m, 4H, 2-Ar, 5-Ar: H-2, H-6, H-2′, H-6′); ^13^C-NMR (100 MHz, DMSO-d_6_): δ 117.4, 124.4, 124.6, 127.5, 129.0, 144.7, 157.3, 164.1, 167.4, 179.1. IR (ATR) ν: 3080, 1598, 1515 cm^−1^; HRMS calcd for (C_14_H_9_N_5_SO_3_ + H^+^): 328.0504; found: 328.0502; Anal. Calcd for C_14_H_9_N_5_SO_3_: C, 51.37; H, 2.77; N, 21.40. Found: C, 51.50; H, 2.78; N, 21.47.

*5-(4-Bromophenyl)-2-(4-hydroxyphenylazo)-1,3,4-thiadiazole* (**6d**). The product was obtained as a brown solid (4.06 g, 75%); mp 263–265 °C; R_f_ (CHCl_3_/EtOAc, 5:1 *v*/*v*) 0.33. ^1^H-NMR (400 MHz, DMSO-d_6_): δ 7.03 (d, 2H, J= 9.2 Hz, 2-Ar: H-3, H-5), 7.80 (d, 2H, *J* = 8.8 Hz, 5-Ar: H-3′, H-5′), 7.94 (d, 2H, *J* = 9.2 Hz, 2-Ar: H-2, H-6), 8.03 (d, 2H, *J* = 8.8 Hz, 5-Ar: H-2′, H-6′); ^13^C-NMR (100 MHz, DMSO-d_6_): δ 116.8, 125.5, 127.1, 129.6, 131.7, 132.5, 144.7, 164.4, 166.4, 179.4. IR (ATR) ν: 3047, 1587, 1507 cm^−1^; HRMS calcd for (C_14_H_9_N_4_SOBr + H^+^): 360.9759; found: 360.9757; Anal. Calcd for C_14_H_9_N_4_SOBr: C, 46.55; H, 2.51; N, 15.51. Found: C, 46.61; H, 2.50; N, 15.59.

*5-(4-t-Butylphenyl)-2-(4-hydroxyphenylazo)-1,3,4-thiadiazole* (**6e**). The product was obtained as an orange solid (4.06 g, 80%); mp 291–293 °C; R_f_ (CHCl_3_/EtOAc, 5:1 *v*/*v*) 0.37. ^1^H-NMR (400 MHz, DMSO-d_6_): δ 1.33 (s, 9H, C(CH_3_)_3_), 7.03 (d, 2H, *J* = 9.2 Hz, 2-Ar: H-3; H-5), 7.60 (d, 2H, *J* = 8.8 Hz, 5-Ar: H-3′, H-5′), 7.94 (d, 2H, *J* = 9.2 Hz, 2-Ar: H-2, H-6), 8.01 (d, 2H, *J* = 8.8 Hz, 5-Ar: H-2′, H-6′); ^13^C-NMR (100 MHz, DMSO-d_6_): δ 30.8, 34.8, 116.7, 126.3, 126.9, 127.6, 129.6, 144.8, 155.1, 164.1, 167.5, 178.8. IR (ATR) ν: 3100, 1582, 1507 cm^−1^; HRMS calcd for (C_18_H_18_N_4_SO + H^+^): 339.1280; found: 339.1281; Anal. Calcd for C_18_H_18_N_4_SO: C, 63.88; H, 5.36; N, 16.56. Found: C, 63.76; H, 5.39; N, 16.51.

## 4. Conclusions

An efficient synthetic strategy for the preparation of new azo dyes using weak heteroaromatic amines containing a 1,3,4-thiadiazole scaffold as the diazo component and aniline, *N,N*-dimethylaniline and phenol as simultaneous coupling precursors, was presented. Three series of azo dyes, the derivatives of 2-phenylazo-5-phenyl-1,3,4-thiadiazole, were obtained in good to excellent yields. The new dyes obtained—demonstrating a broad color spectrum and good solubility in some polar solvents—may serve as potential candidates for the dye industry and will be tested in the near future.

## Figures and Tables

**Table 1:** Most important electronic transitions. H letter indicates Highest Occupied Molecular Orbital HOMO, L indicates Lowest Unoccupied Molecular Orbital LUMO and +/− (number) represent subsequent orbitals above HOMO and LUMO, respectively. The logarithm of molar absorption coefficients log ε and oscillator strengths are given in parenthesis under the wavelengths, respectively for experimental and calculated maxima. The letters **a**–**e** stand for the particular compound in a given series (**4**, **5** or **6**).

λ_max_ (nm)										The Most Important Orbitals Involved in Electronic Transitions	Character of Transition
Experimental					Calculated						
**4 (G=NH_2_)**											
**a**	**b**	**c**	**d**	**e**	**a**	**b**	**c**	**d**	**e**		
237 (3.95)	256 (4.05)	267 (4.07)	249 (3.90)	248 (4.02)	261.44 (0.0909)	276.70 (0.1045)	237.50 (0.0536)	256.39 (0.0432)	270.17 (0.2800)	(**a**, e)H-2→L+1 (**b**)H→L+2 (**c**)H-2→L+2 (**d**)H-8→L	(**a**, **e**)n(NH_2_)/π→π* (**b**)n(NH_2_)/n(MeO)/π→π*(C6-rings) (**c**)n(NH_2_)/n(NO_2_)/π→π* (**d**)n(Br)/π(S-ring)→π*
					262.74 (0.0776)	280.67 (0.2599)	253.28 (0.0806)	274.45 (0.3976)	273.29 (0.1360)	(**a**)H-6→L (**b**)H-1→L+1 (**c**)H-6→L+1 (**d**)H-2→L+1 (**e**) H→L+2	(**a**)n(NH_2_)/π→π* (**b**)n(NH_2_)/n(MeO)/π→π* (**c**)π→π* (**d**)n(Br)/n(NH_2_)/π→π* (**e**)n(NH_2_)/π→π*(C6-rings)
					266.87 (0.1051)		268.23 (0.0893)			(**a**)H→L+3 (**c**)H→L+4	(**a**, **c**)n(NH_2_)/π→π*(C6-rings)
					272.57 (0.1083)					(**a**)H→L+2	(**a**)n(NH_2_)/π→π*(C6-rings)
285 (3.72)	286 (3.88)		286 (3.63)	284 (3.79)							
316 (3.56)	319 (3.78)	321 (4.03)	318 (3.56)	319 (3.68)	325.66 (0.0203)	322.13 (0.0500)	312.40 (0.0522)	333.77 (0.0204)	324.73 (0.0294)	(**a**, **b**, **d**, **e**)H→L+1 (**c**)H-2→L+1	(**a**, **c**, **e**)n(NH_2_)/π→π* (**b**)n(NH_2_)/n(MeO)/π→π* (**d**)n(Br)/n(NH_2_)/π→π*
							344.08 (0.1372)			(**c**)H-2→L	(**c**)n(NH_2_)/π→π*
491 (4.52)	491 (4.46)	507 (4.35)	496 (4.41)	492 (4.47)		383.91 (0.1281)	428.57 (0.7203)			(**b**)H-1→L (**c**)H→L+1	(**b**)n(NH_2_)/n(MeO)/π→π* (**c**)n(NH_2_)/π→π*
					459.54 (1.4831)	478.70 (1.3233)	529.34 (0.8884)	464.44 (1.4918)	464.82 (1.4522)	H→L	(**a**, **c**, **e**)n(NH_2_)/π→π* (**b**) n(NH_2_)/n(MeO)/π→π* (**d**)n(Br)/n(NH_2_)/π→π*
**5 (G=NMe_2_)**											
**a**	**b**	**c**	**d**	**e**	**a**	**b**	**c**	**d**	**e**		
243 (3.96)	256 (3.83)	249 (3.73)	251 (3.80)	245 (4.06)	264.95 (0.1359)	278.83 (0.0592)	241.40 (0.0442)	256.30 (0.0430)	270.21 (0.0655)	(**a**)H-2→L+1 (**b**, **e**) H-1→L+1 (**c**)H-2→L+2 (**d**)H-8→L	(**a**)π→π* (**b**) n(MeO)/n(NMe_2_)/π→π* (**c**)n(NO_2_)/π→π* (**d**)n(Br)/π→π* (**e**)σ(*t*-Bu)/n(Me_2_)/π→π*
					275.87 (0.1374)	283.75 (0.1408)	261.09 (0.0967)	274.64 (0.1508)	270.38 (0.0853)	(**a**)H-6→L (**b**)H→L+2 (**c**)H-6→L+1 (**d**)H-2→L+1 (**e**)H-1→L+1	(**a**, **c**)π→π* (**b**)n(MeO)/n(NMe_2_)/π→π*(C6-ring near MeO) (**d**)n(Br)/n(NMe_2_)/π→π* (**e**)σ(*t*-Bu)/n(Me_2_)/π→π*
					281.40 (0.0925)	287.77 (0.2354)	277.34 (0.1087)	282.30 (0.2530)	279.49 (0.2116)	(**a**)H→L+3 (**b**, **d**)H-5→L (**c**)H→L+4 (**e**)H-6→L	(**a**)n(NMe_2_)/π→π* (**b**)n(MeO)/π→π* (**c**)n(NMe_2_)/π→π*(C6-ring near NMe_2_) (**d**)n(Br)/π→π* (**e**)σ(*t*-Bu)/π→π*
									281.87 (0.0869)	(**e**)H→L+2	(**e**)n(NMe_2_)/π→π*(C6-rings)
293 (3.79)	293 (3.73)	299 (3.85)	290 (3.61)	295 (3.92)							
320 (3.68)	334 (3.46)		325 (3.49)	323 (3.80)	337.91 (0.0197)	333.19 (0.0329)	353.80 (0.1689)	346.81 (0.0206)	336.73 (0.0263)	(**a**, **b**, **d**, **e**)H→L+1 (**c**)H-2→L	(**a**, **d**, **e**)n(NMe_2_)/π→π* (**b**)n(MeO)/n(NMe_2_)/π→π* (**c**)n(NO_2_)/π→π*
515 (4.50)	515 (4.25)	530 (3.95)	520 (4.30)	515 (4.41)		393.91 (0.0395)	453.15 (0.8353)			(**b**)H-1→L (**c**)H→L+1	(**b**)n(MeO)/n(NMe_2_)/π→π* (**c**)n(NMe_2_)/π→π*
					483.86 (1.4831)	497.00 (1.1993)	568.89 (0.8657)	488.69 (1.5864)	487.35 (1.5439)	H→L	(**a**, **c**, **d**, **e**)n(NMe_2_)/π→π* (**b**)n(MeO)/n(NMe_2_)/π→π*
**6 (G=OH)**											
**a**	**b**	**c**	**d**	**e**	**a**	**b**	**c**	**d**	**e**		
						220.52 (0.0983)				(**b**)H-4→L+1	(**b**)π(C6-ring near OH)→π*
236 (4.00)		231 (3.68)			234.73 (0.0439)		232.01 (0.0382)			(**a**)H→L+3 (**c**)H-2→L+2	(**a**)n(OH)/π→π*(C6-ring near S-ring) (**c**)n(NO_2_)/(OH)/π→π*
256 (3.90)	254 (4.04)	260 (3.72)	245 (4.04)	243 (4.10)	251.23 (0.0758)	234.51 (0.0525)	249.20 (0.0821)	238.98 (0.0306)	234.61 (0.0464)	(**a**, **e**)H→L+2 (**b**)H-3→L+1 (**c**)H-8→L (**d**)H-4→L+1	(**a**)n(OH)/π→π*(C6-ring near OH) (**b**)π(C6-ring near MeO)→π* (**c**)n(NO_2_)/(OH)/π→π* (**d**)π(C6-rings)→π* (**e**)σ(*t*-Bu)/n(OH)/π→π*(C6-ring near *t*-Bu)
					254.98 (0.1078)	271.99 (0.1002)	264.75 (0.0369)	251.23 (0.0556)	265.07 (0.2413)	(**a**)H-2→L+1 (**b**, **c**, **e**)H-6→L (**d**)H→L+3	(**a**, **c**)n(OH)/π→π* (**b**)n(MeO)/n(OH)/π→π* (**d**)n(Br)/n(OH)/π→π*(C6-ring near OH) (**e**)σ(*t*-Bu)/n(OH)/π→π*
					262.06 (0.1766)			266.81 (0.1192)		(**a**)H-6→L+1 (**d**)H-2→L+1	(**a**)n(OH)/π→π* (**d**)n(Br)/n(OH)/π→π*
								270.78 (0.1692)		(**d**)H-6→L	(**d**)n(Br)/n(OH)/π→π*
298 (3.54)	291 (3.74)		290 (3.76)	290 (3.64)	297.41 (0.0427)	302.67 (0.1542)		304.17 (0.0596)	298.30 (0.0985)	(**a**, **b**, **c**, **e**)H→L+1	(**a**)n(OH)/π→π* (**b**)n(MeO)/n(OH)/π→π* (**d**)n(Br)/n(OH)/π→π* (**e**)σ(*t*-Bu)/n(OH)/π→π*
					325.44 (0.0438)					(**a**)H-4→L	(**a**)π(C6-ring near OH)→π*
		310 (3.74)					305.19 (0.1361)			(**c**)H-2→L+1	(**c**)n(NO_2_)/(OH)/π→π*
405 (4.46)	415 (4.35)	410 (4.13)	409 (4.28)	409 (3.96)			341.08 (0.0630)			(**c**)H-3→L	(**c**)π(C6-ring near OH)→π*
					345.44 (0.0461)	370.43 (0.3618)	385.35 (0.2630)	355.56 (0.0770)	355.42 (0.1288)	(**a**, **d**, **e**)H-2→L (**b**)H-1→L (**c**)H-1→L+1	(**a**, **c**)n(OH)/π→π* (**b**)n(MeO)/n(OH)/π→π* (**d**)n(Br)/n(OH)/π→π* (**e**)σ(*t*-Bu)/n(OH)/π→π*
					432.77 (1.1904)	471.46 (0.9677)	458.02 (1.1983)	439.27 (1.2728)	444.00 (1.1983)	H→L	(**a**, **c**)n(OH)/π→π* (**b**)n(MeO)/n(OH)/π→π* (**d**)n(Br)/n(OH)/π→π* (**e**)σ(*t*-Bu)/n(OH)/π→π*

Used abbreviations: n—non-bonding orbital; S-ring—thiadiazole ring; C6-ring—benzene ring; *—antibonding orbital.

**Table 2:** Selected structural data of studied compound **5b**.

i—j	d_ij_ [Å]	i—j—k	α_ijk_ [°]	i—j—k	α_ijk_ [°]
S1—C1	1.7462(14)	C1—S1—C2	86.22(7)	C2—C11—C16	122.25(13)
S1—C2	1.7366(14)	S1—C2—N2	114.29(11)	C5—C6—N5	121.16(12)
C1—N1	1.3092(18)	C2—N2—N1	112.80(12)	C7—C6—N5	121.27(12)
C2—N2	1.3124(18)	N2—N1—C1	112.25(12)	C6—N5—C9	120.76(12)
N1—N2	1.3757(17)	N1—C1—S1	114.42(11)	C6—N5—C10	120.34(12)
C1—N3	1.3821(18)	N1—C1—N3	120.62(13)	C9—N5—C10	118.16(12)
N3—N4	1.2907(16)	S1—C1—N3	124.91(10)	C13—C14—O1	115.86(12)
N4—C3	1.3851(18)	C1—N3—N4	111.67(11)	C15—C14—O1	124.38(13)
C2—C11	1.4651(19)	N3—N4—C3	115.29(11)	C14—O1—C17	117.14(11)
N5—C6	1.3520(18)	N4—C3—C4	125.47(12)		
N5—C9	1.4635(18)	N4—C3—C8	116.30(12)		
N5—C10	1.4606(18)	S1—C2—C11	122.98(10)		
O1—C14	1.3610(17)	N2—C2—C11	122.55(13)		
O1—C17	1.4346(17)	C2—C11—C12	118.81(13)		

**Table 3:** Non-classic hydrogen bonds and the first level graph motifs in the studied compound. Cg(C3) indicates centroid of ring containing C3 atom.

D-H•••A	d(D-H) [Å]	d(H•••A) [Å]	d(D•••A) [Å]	<(DHA) [°]	G_d_^a^(n)
C9—H9A•••N1 ^i^	0.98	2.58	3.5351(1)	165.4	R_2_^2^(22)
C10—H10B•••O1 ^ii^	0.98	2.54	3.5031(1)	166.3	C(17)
C16—H16•••S1	0.95	2.86	3.2088(1)	102.7	S(5)
C7—H7•••Cg(C3) ^iii^	0.95	2.97	3.8190(1)	148.8	C(2)

Symmetry transformations used to generate equivalent atoms: (i) −x + 1, −y + 1, −z − 2; (ii) x + 1, y, z; (iii) x, −y + 1.5, z − 0.5.

**Table 4:** Interactions in the studied compound. Each ring is indicated by one atom, which belongs solely to this ring. α is a dihedral angle between planes I and J, β is an angle between Cg(I)-Cg(J) vector and normal to plane I and d_p_ is the perpendicular distance of Cg(I) on ring J plane.

R(I)•••R(J)	d(Cg•••Cg) [Å]	α [°]	β [°]	d_p_ [Å]
C3•••C3 ^iv^	3.6217(1)	0	15.5	3.4908
C11•••C11 ^v^	3.5355(1)	0	22.3	3.2703

Symmetry transformations used to generate equivalent atoms: (iv) −x+1, −y+1, −z+1;(v) −x, −y+1, −z+1.

## Supplementary Materials

Copies of the 1H-NMR, 13C-NMR, UV-Vis and IR spectra of the compounds as well as figures showing the calculated spectra and the contour plots of the most important molecular orbitals are available in the online Supplementary Materials.
