# Supplementary material for: 1,3,4-Thiadiazole-Containing Azo Dyes: Synthesis, Spectroscopic Properties and Molecular Structure
Source: Molecules. 2020 Jun 18;25(12):2822. doi: 10.3390/molecules25122822 (PMC7356117; doi:10.3390/molecules25122822)

Supplementary Material

1,3,4-Thiadiazole-containing Azo Dyes: Synthesis, Spectroscopic Properties and Molecular Structure

Agnieszka Kudelko [1,*], Monika Olesiejuk [1], Marcin Luczynski [1], Marcin Swiatkowski [2], Tomasz Sieranski [2], Rafal Kruszynski [2]

[1] *Department of Chemical Organic Technology and Petrochemistry, The Silesian University of Technology, Krzywoustego 4, PL-44100 Gliwice, Poland*

[2] *Institute of General and Ecological Chemistry, Lodz University of Technology, Żeromskiego 116, PL-90924 Łódź, Poland*

** Corresponding author*

E-mail: Agnieszka.Kudelko@polsl.pl (A. Kudelko).

Table of contents

1. ^{1}H and ^{13}C NMR spectra 3

2. UV-Vis absorption data for compounds **4a-e**, **5a-e**, **6a-e** 33

3. Plots of the most important molecular orbitals of compounds **4a-e**, **5a-e**, **6a-e** 39

4. FT-IR spectra of compounds **4a-e**, **5a-e**, **6a-e** 42

5. Crystal structure determination 57

6. CheckCIF file for **5b**……..59

1. ^{1}H and ^{13}C NMR spectra

2-(4-Aminophenylazo)-5-phenyl-1,3,4-thiadiazole (**4a**)

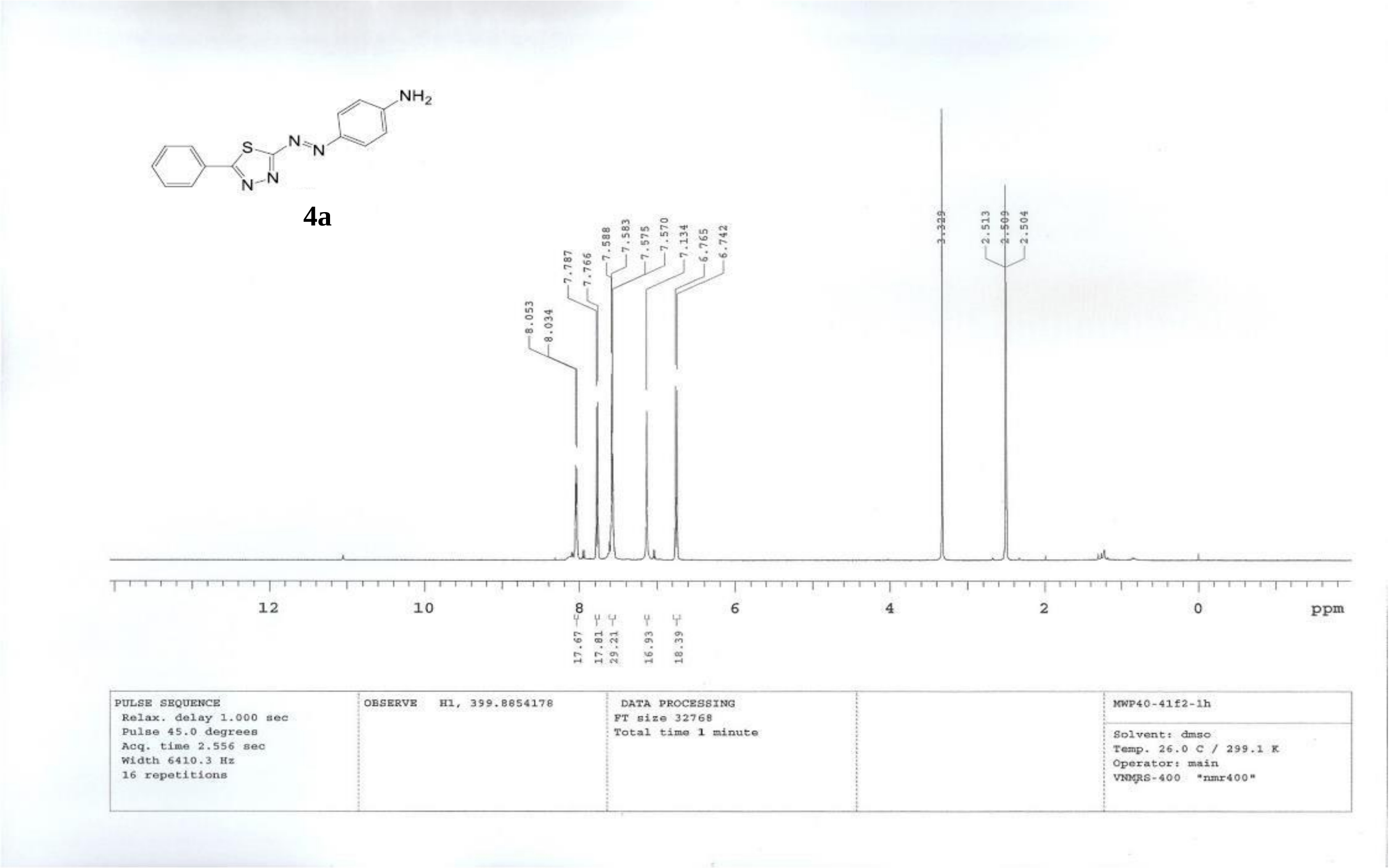

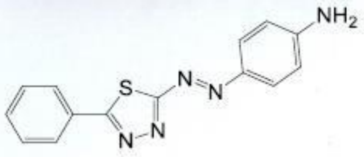
S
N
N
N
N
NH2

4a

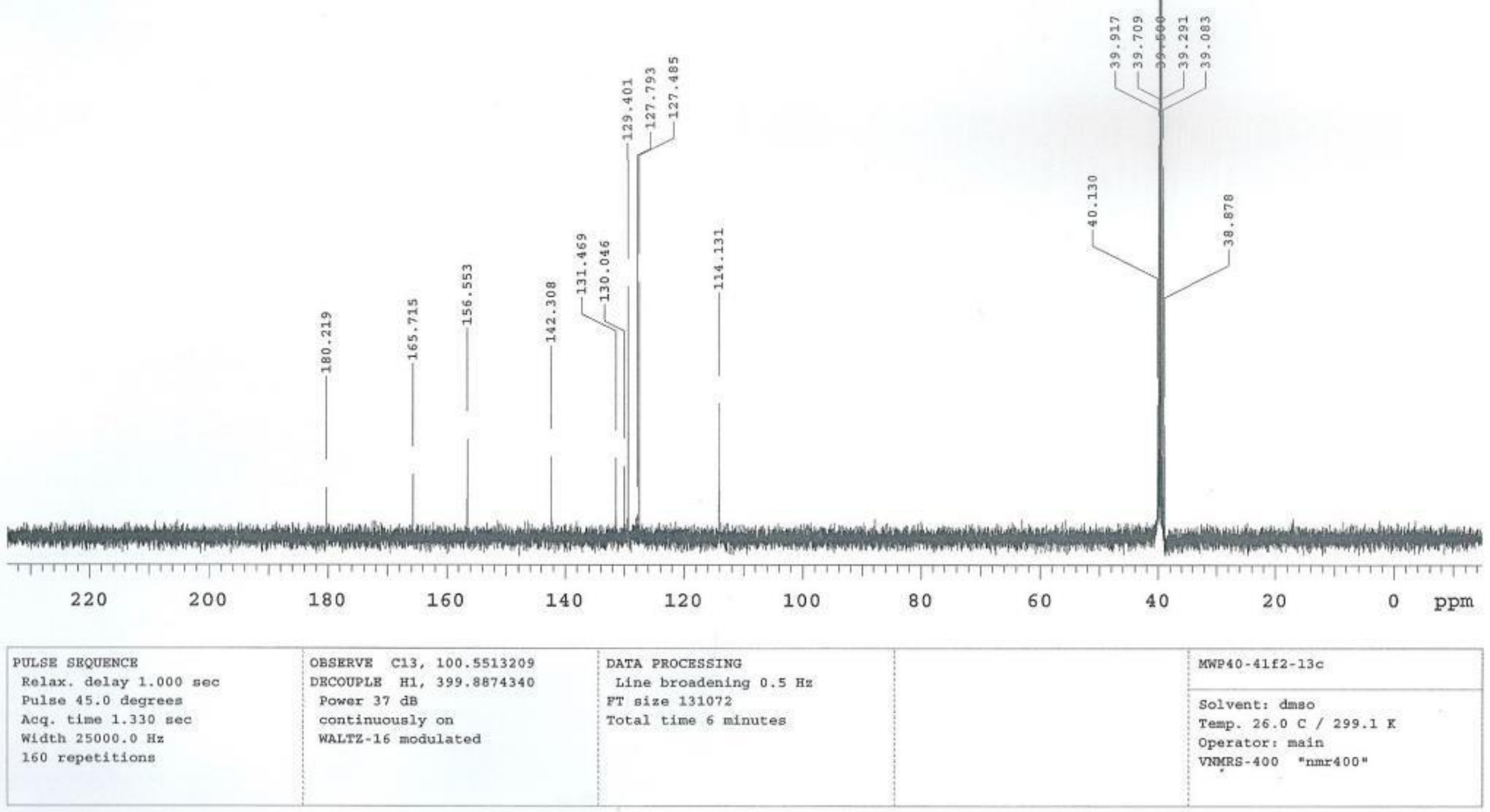
180.219
165.715
156.553
142.308
131.469
130.046
129.401
127.793
127.485
114.131
40.130
39.917
39.709
39.500
39.291
39.083
38.878
220 200 180 160 140 120 100 80 60 40 20 0 ppm
PULSE SEQUENCE
Relax. delay 1.000 sec
Pulse 45.0 degrees
Acq. time 1.330 sec
Width 25000.0 Hz
160 repetitions
OBSERVE C13, 100.5513209
DECOUPLE H1, 399.8874340
Power 37 dB
continuously on
WALTZ-16 modulated
DATA PROCESSING
Line broadening 0.5 Hz
FT size 131072
Total time 6 minutes
MWP40-41f2-13c
Solvent: dmso
Temp. 26.0 C / 299.1 K
Operator: main
VNMRS-400 "nmr400"

2-(4-Aminophenylazo)-5-(4-methoxyphenyl)-1,3,4-thiadiazole (**4b**)

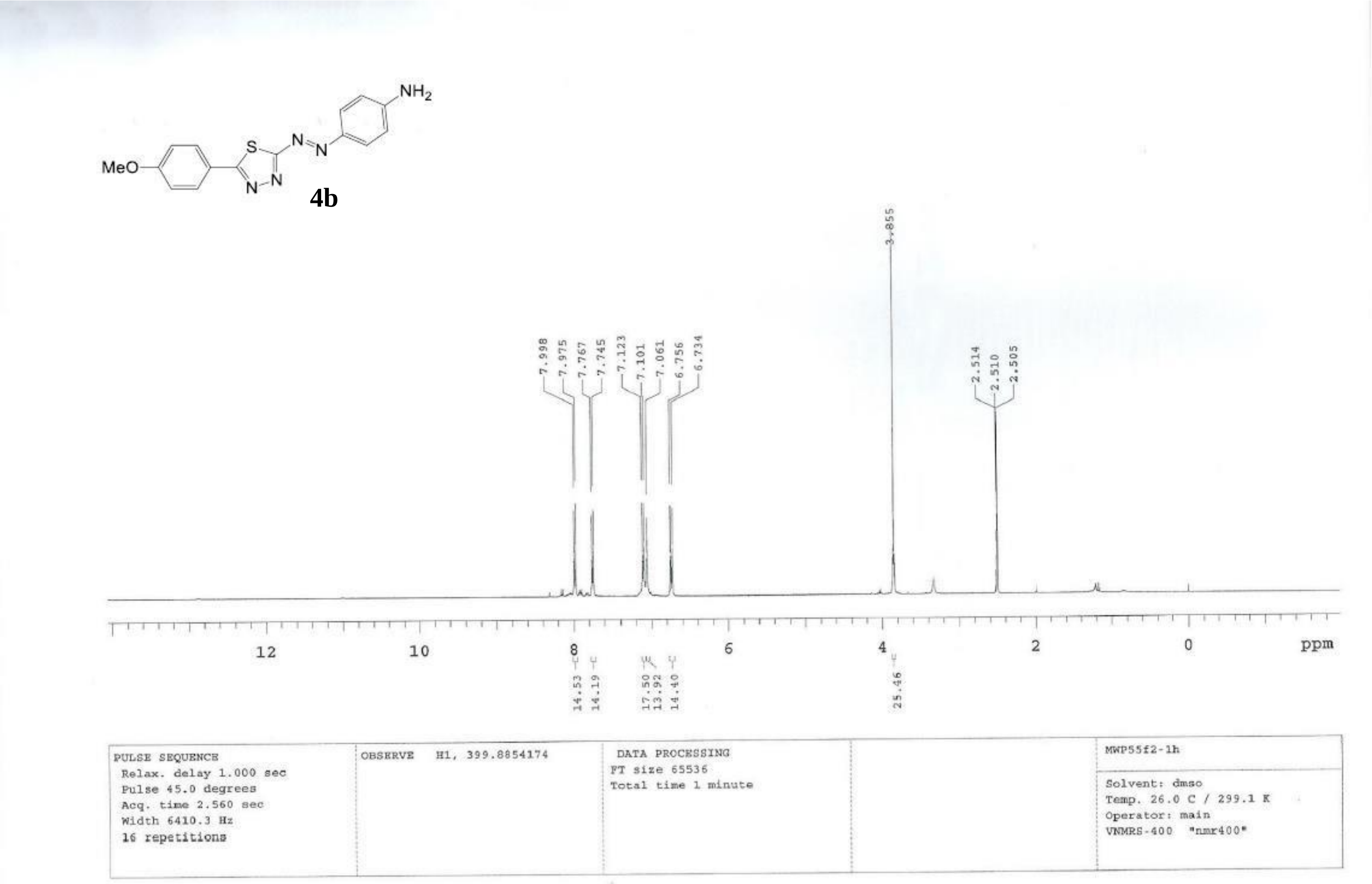

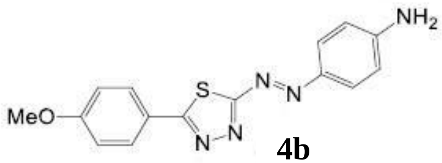
NH2
MeO
S
N
N
N-N

4b

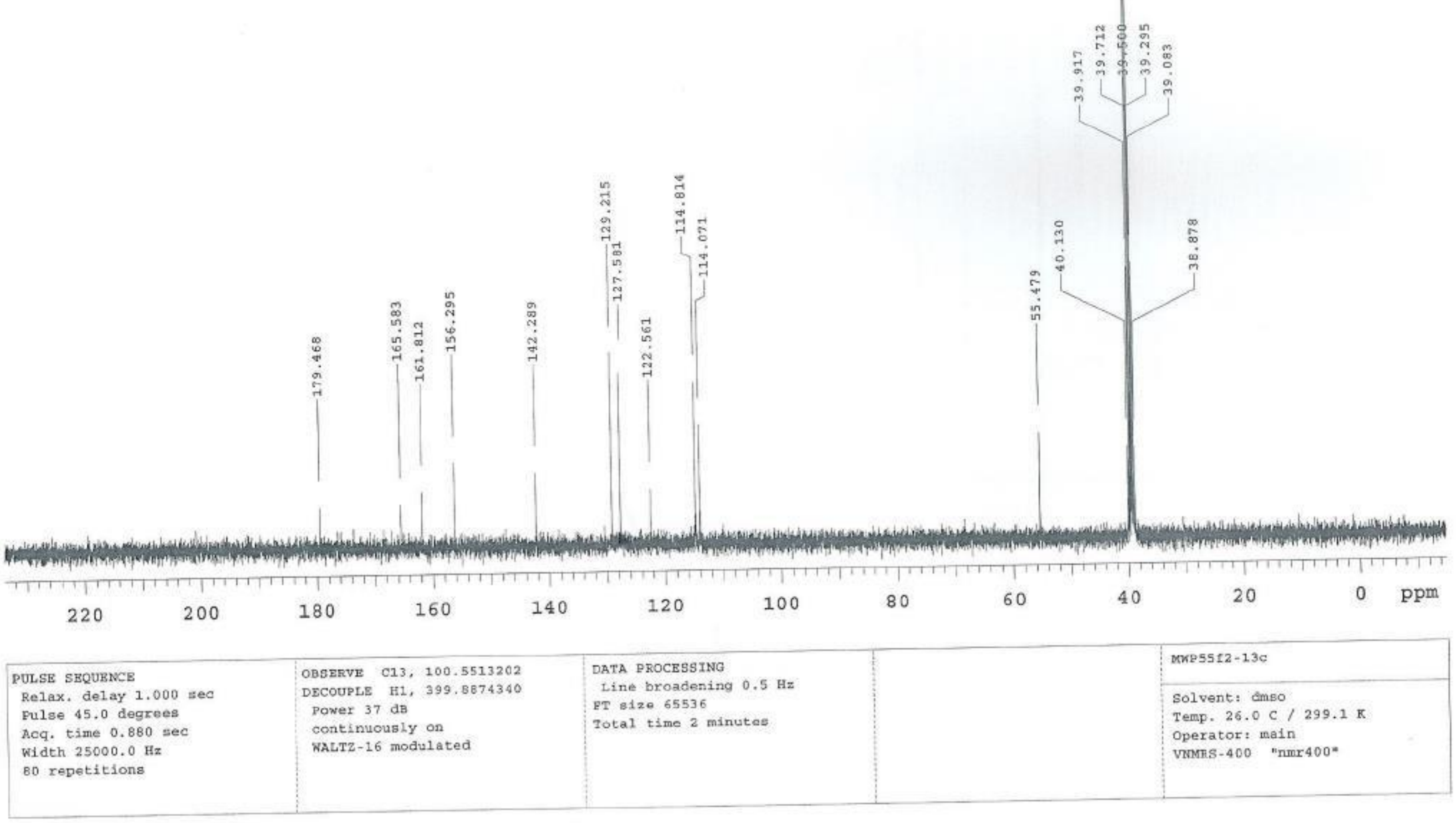
179.468
165.583
161.812
156.295
142.289
129.215
127.581
122.561
114.814
114.071
55.479
40.130
39.917
39.712
39.500
39.295
39.083
38.878
220
200
180
160
140
120
100
80
60
40
20
0
ppm
PULSE SEQUENCE
Relax. delay 1.000 sec
Pulse 45.0 degrees
Acq. time 0.880 sec
Width 25000.0 Hz
80 repetitions
OBSERVE C13, 100.5513202
DECOUPLE H1, 399.8874340
Power 37 dB
continuously on
WALTZ-16 modulated
DATA PROCESSING
Line broadening 0.5 Hz
FT size 65536
Total time 2 minutes
MWP55f2-13c
Solvent: dmso
Temp. 26.0 C / 299.1 K
Operator: main
VNMRS-400 "nmr400"

2-(4-Aminophenylazo)-5-(4-nitrophenyl)-1,3,4-thiadiazole (**4c**)

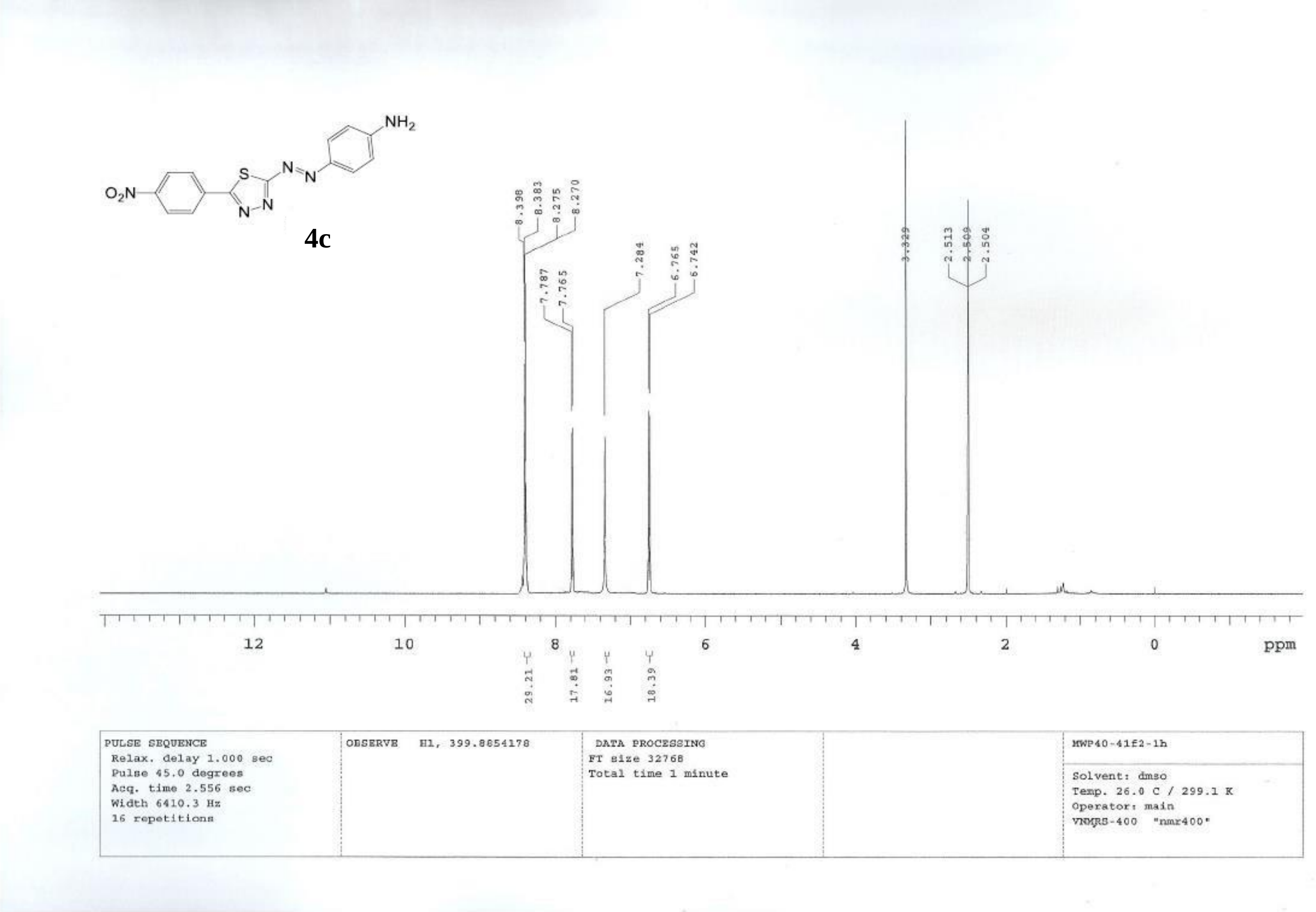

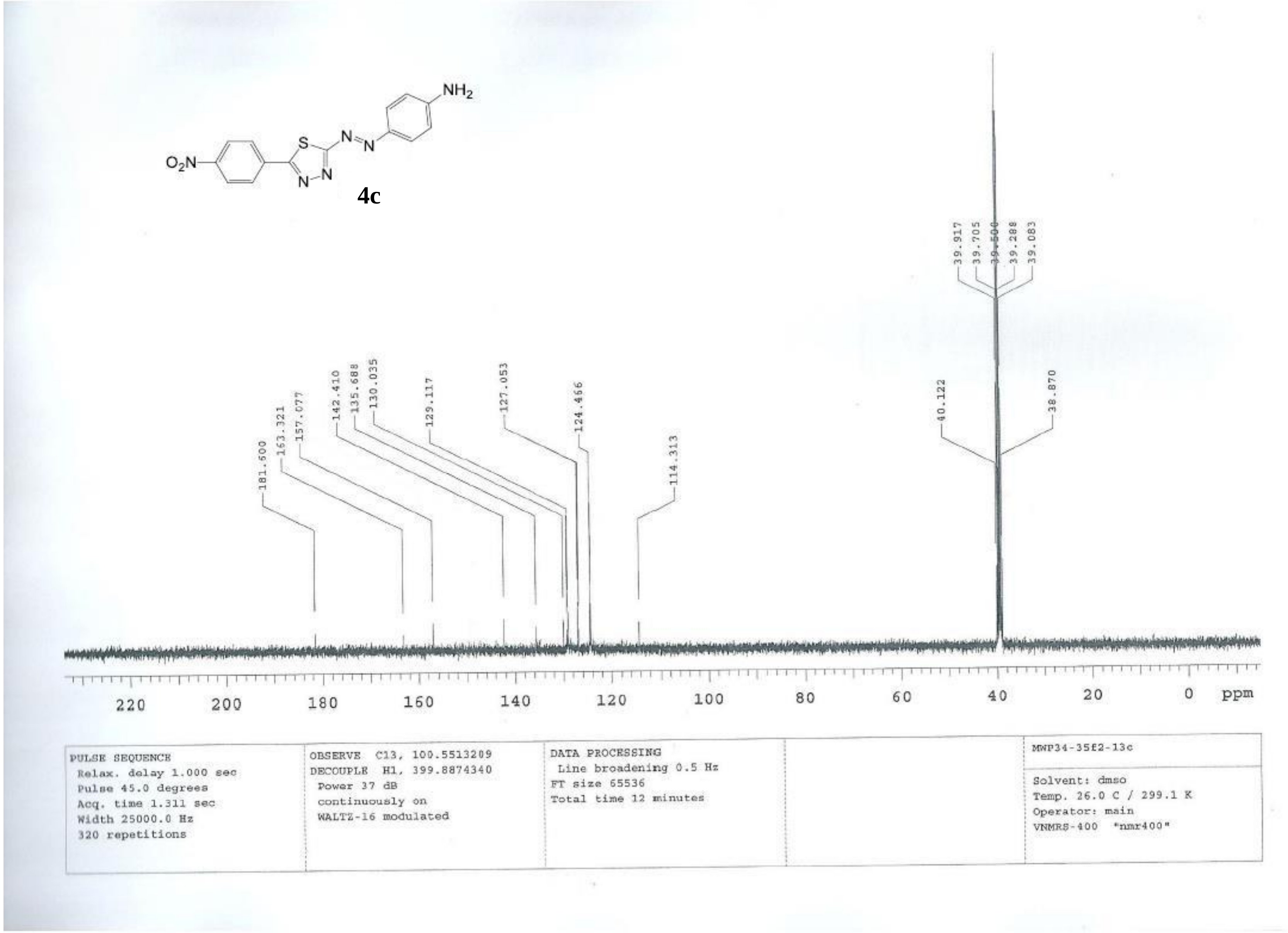

NH_2
S
N=N
O_2N
N–N
4c
39.917
39.705
39.500
39.288
39.083
181.600
163.321
157.077
142.410
135.688
130.035
129.117
127.053
124.466
114.313
40.122
38.870
220
200
180
160
140
120
100
80
60
40
20
0
ppm
PULSE SEQUENCE
Relax. delay 1.000 sec
Pulse 45.0 degrees
Acq. time 1.311 sec
Width 25000.0 Hz
320 repetitions
OBSERVE C13, 100.5513209
DECOUPLE H1, 399.8874340
Power 37 dB
continuously on
WALTZ-16 modulated
DATA PROCESSING
Line broadening 0.5 Hz
FT size 65536
Total time 12 minutes
MWP34-35f2-13c
Solvent: dmso
Temp. 26.0 C / 299.1 K
Operator: main
VNMRS-400 "nmr400"

2-(4-Aminophenylazo)-5-(4-bromophenyl)-1,3,4-thiadiazole (**4d**)

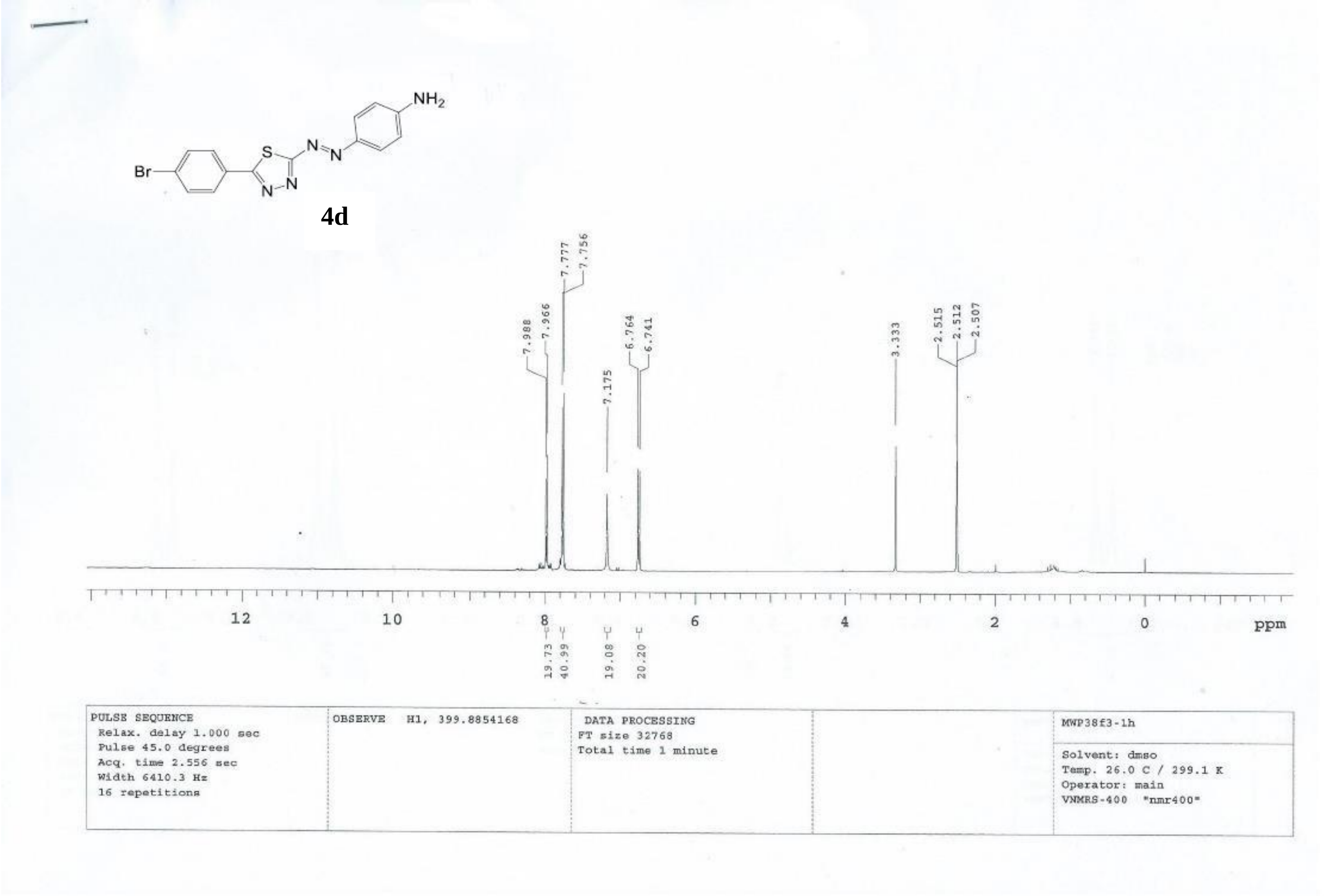

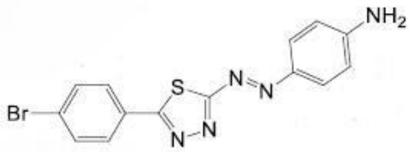
NH2
N=N
S
N–N
Br

4d

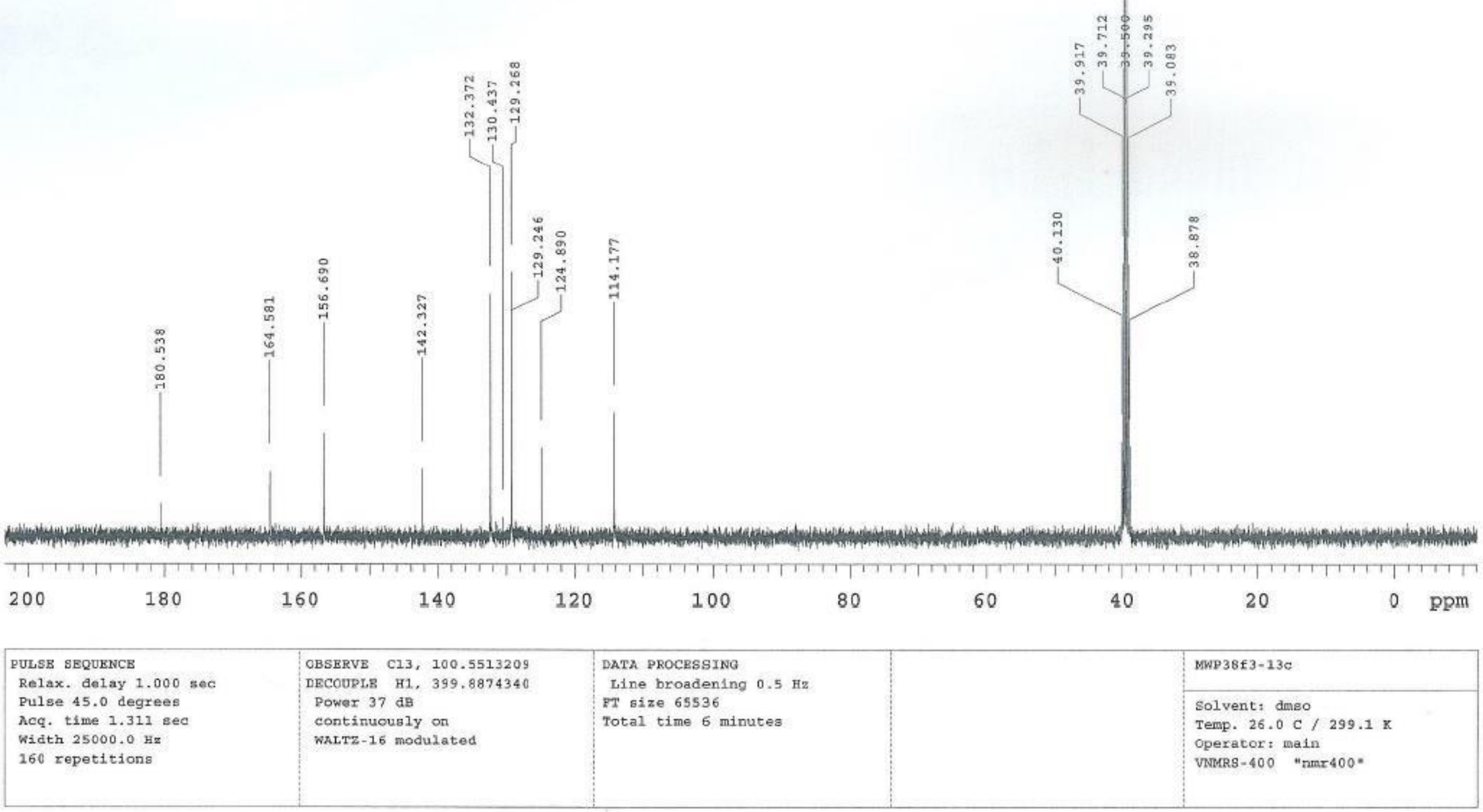
180.538
164.581
156.690
142.327
132.372
130.437
129.268
129.246
124.890
114.177
40.130
39.917
39.712
39.500
39.295
39.083
38.878
200 180 160 140 120 100 80 60 40 20 0 ppm
PULSE SEQUENCE
Relax. delay 1.000 sec
Pulse 45.0 degrees
Acq. time 1.311 sec
Width 25000.0 Hz
160 repetitions
OBSERVE C13, 100.5513209
DECOUPLE H1, 399.8874340
Power 37 dB
continuously on
WALTZ-16 modulated
DATA PROCESSING
Line broadening 0.5 Hz
FT size 65536
Total time 6 minutes
MWP38f3-13c
Solvent: dmso
Temp. 26.0 C / 299.1 K
Operator: main
VNMRS-400 "nmr400"

2-(4-Aminophenylazo)-5-(4-t-butylphenyl)-1,3,4-thiadiazole (**4e**)

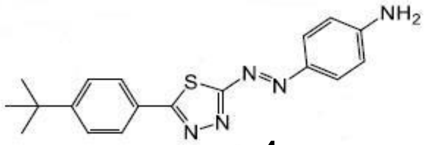

4e

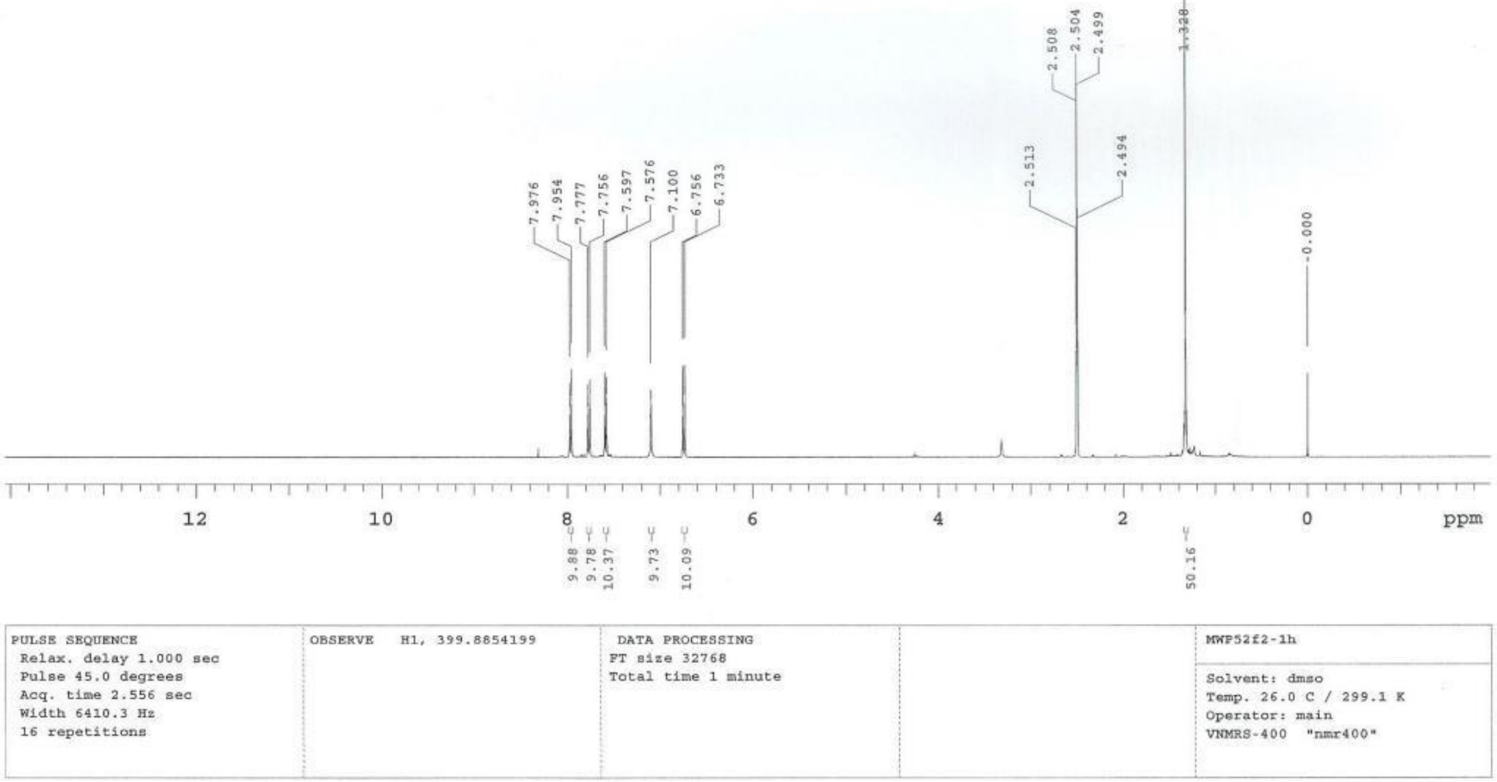

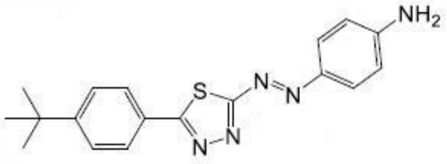

4e

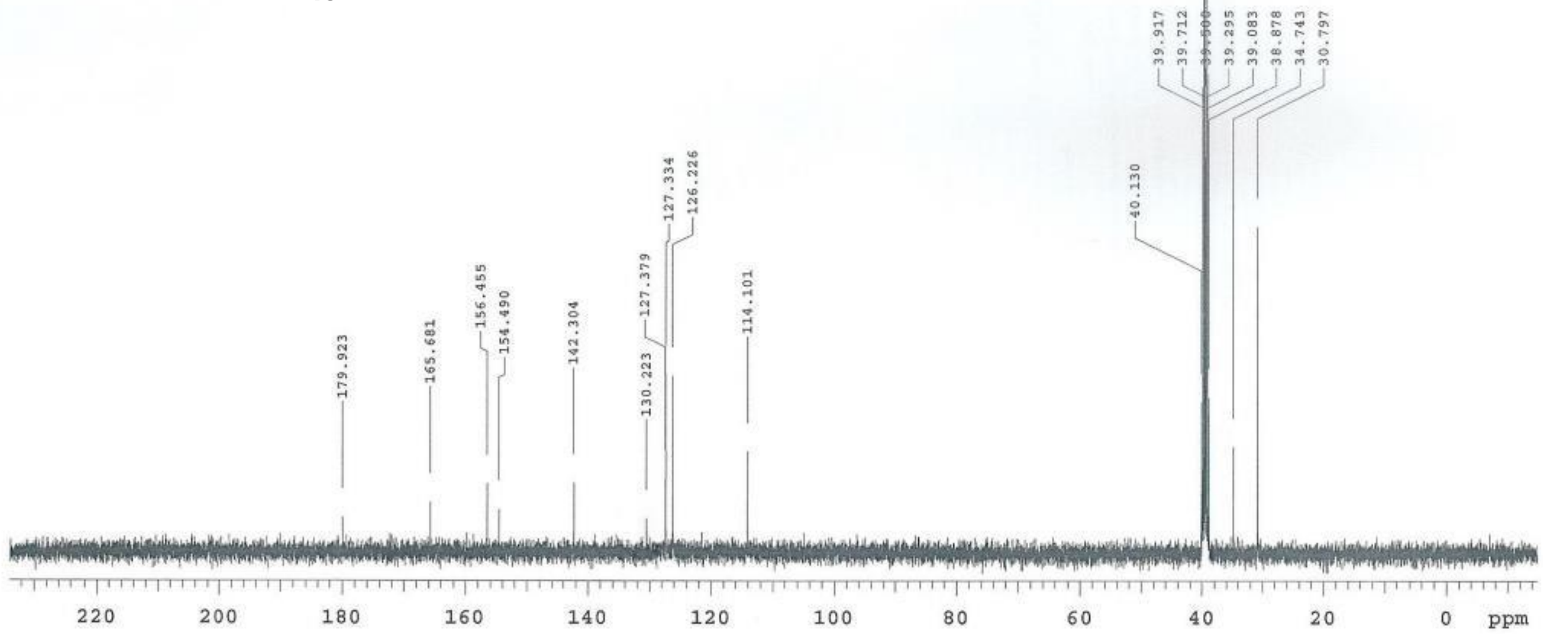

PULSE SEQUENCE
Relax. delay 1.000 sec
Pulse 45.0 degrees
Acq. time 1.311 sec
Width 25000.0 Hz
288 repetitions

OBSERVE C13, 100.5513209
DECOUPLE H1, 399.8874340
Power 37 dB
continuously on
WALTZ-16 modulated

DATA PROCESSING
Line broadening 0.5 Hz
FT size 65536
Total time 11 minutes

MWP52f2-13c

Solvent: dmso
Temp. 26.0 C / 299.1 K
Operator: main
VNMRS-400 "nmr400"

2-[4-(*N*,*N*-Dimethylamino)phenylazo]-5-phenyl-1,3,4-thiadiazole (**5a**)

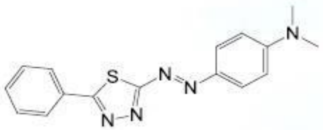

5a

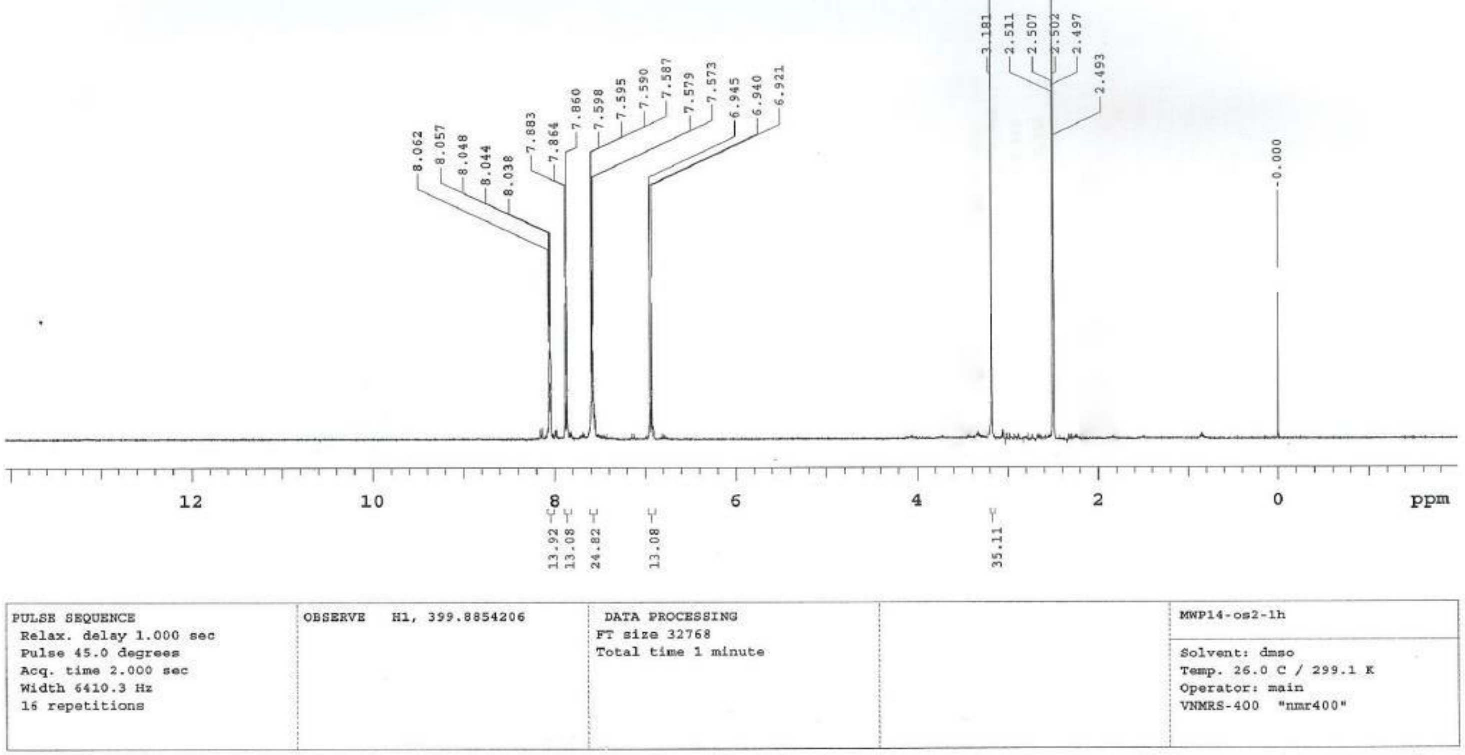

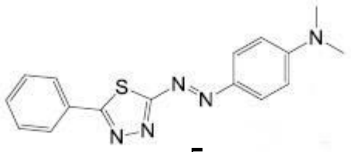

5a

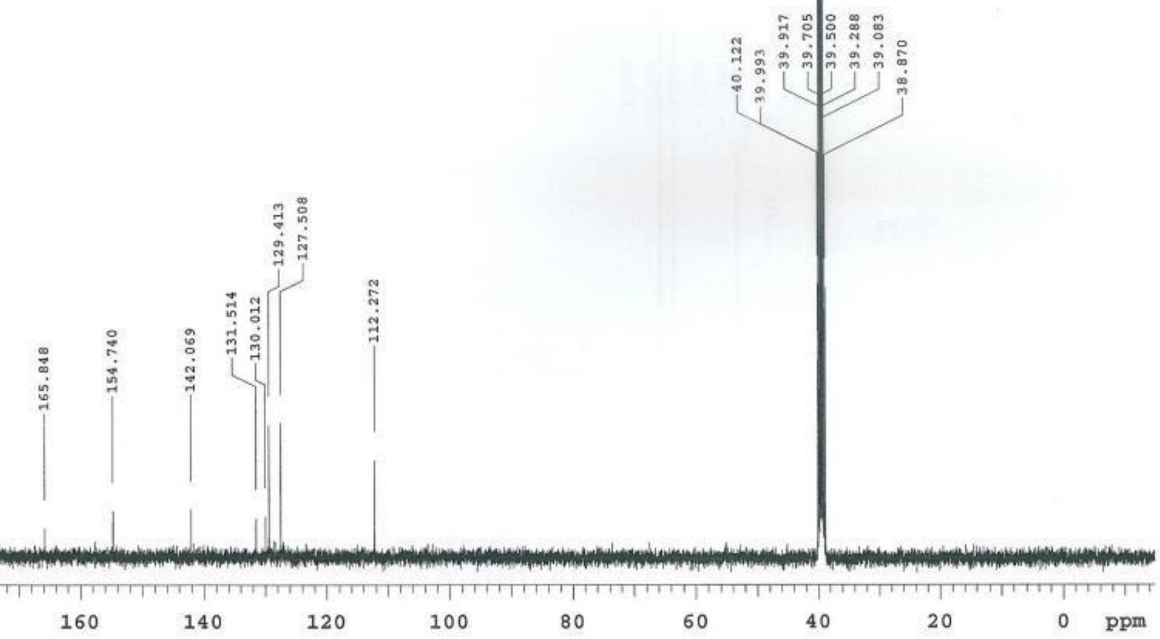

PULSE SEQUENCE
Relax. delay 2.000 sec
Pulse 45.0 degrees
Acq. time 1.311 sec
Width 25000.0 Hz
1000 repetitions

OBSERVE C13, 100.5513217
DECOUPLE H1, 399.8874340
Power 37 dB
continuously on
WALTZ-16 modulated

DATA PROCESSING
Line broadening 0.5 Hz
FT size 65536
Total time 55 minutes

MWP14-os2-13c

Solvent: dmso
Temp. 26.0 C / 299.1 K
Operator: main
VNMRS-400 "nmr400"

2-[4-(*N*,*N*-Dimethylamino)phenylazo]-5-(4-methoxyphenyl)-1,3,4-thiadiazole (**5b**)

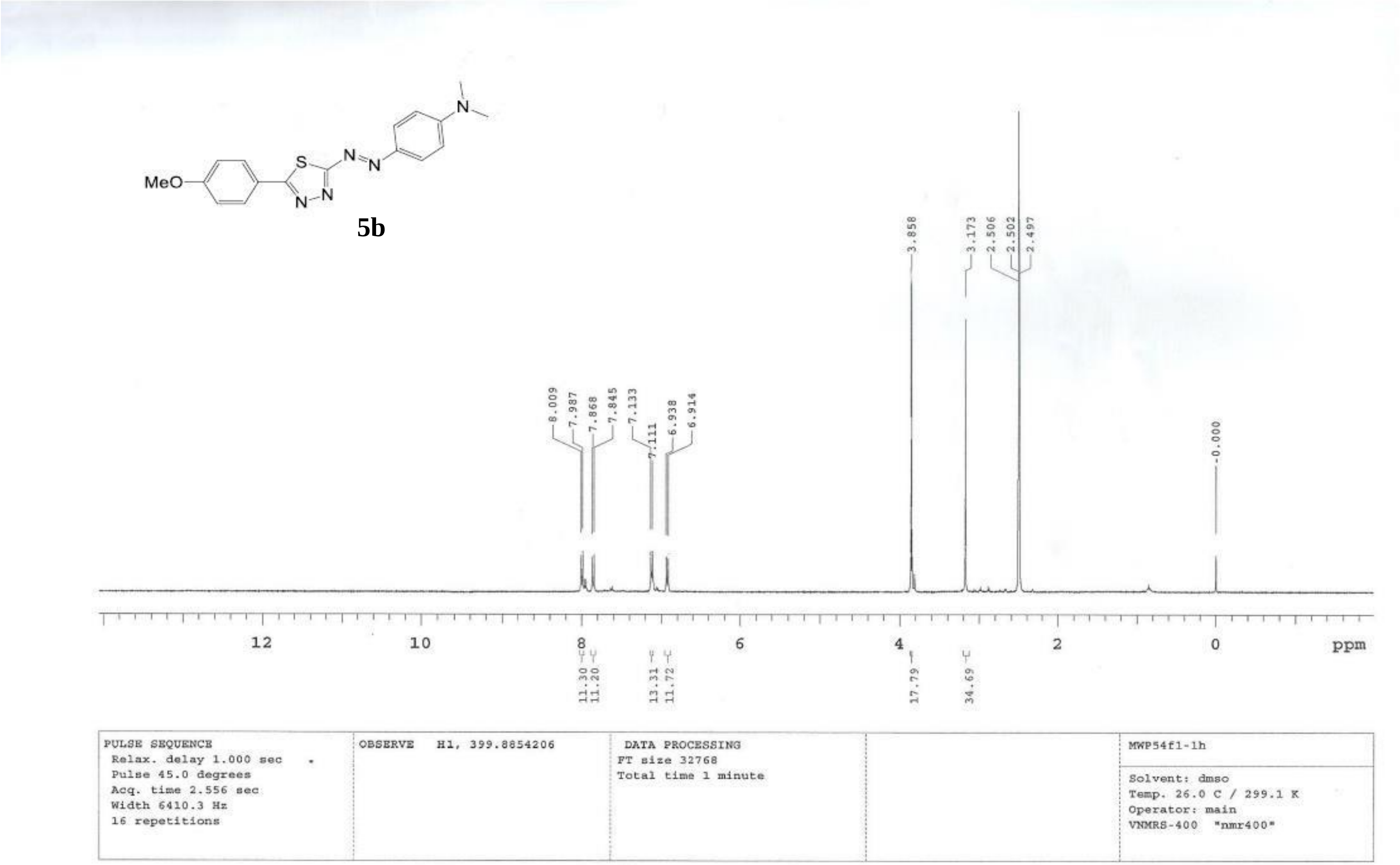

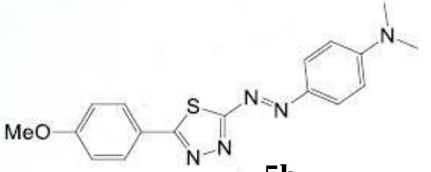

5b

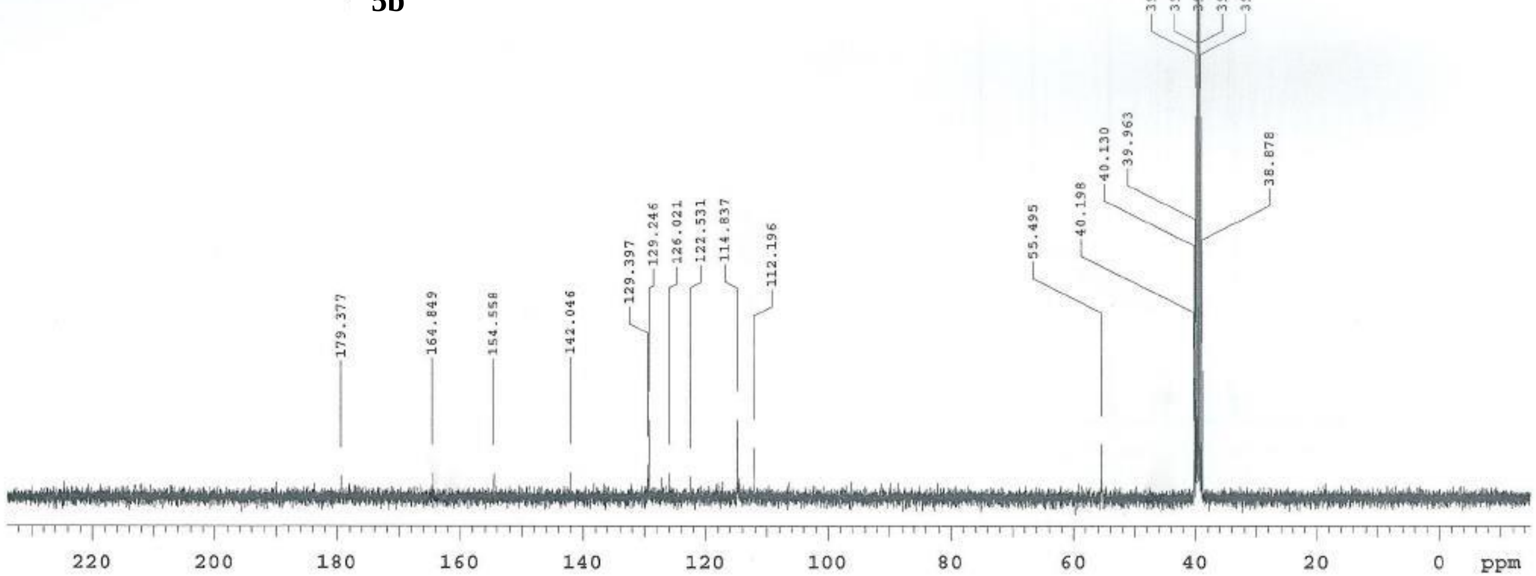

PULSE SEQUENCE	OBSERVE C13, 100.5513202	DATA PROCESSING		MWP54-13c
Relax. delay 1.000 sec	DECOUPLE H1, 399.8874340	Line broadening 0.5 Hz		
Pulse 45.0 degrees	Power 37 dB	FT size 65536		Solvent: dmso
Acq. time 1.311 sec	continuously on	Total time 12 minutes		Temp. 26.0 C / 299.1 K
Width 25000.0 Hz	WALTZ-16 modulated			Operator: main
320 repetitions				VNMRS-400 "nmr400"

2-[4-(*N*,*N*-Dimethylamino)phenylazo]-5-(4-nitrophenyl)-1,3,4-thiadiazole (**5c**)

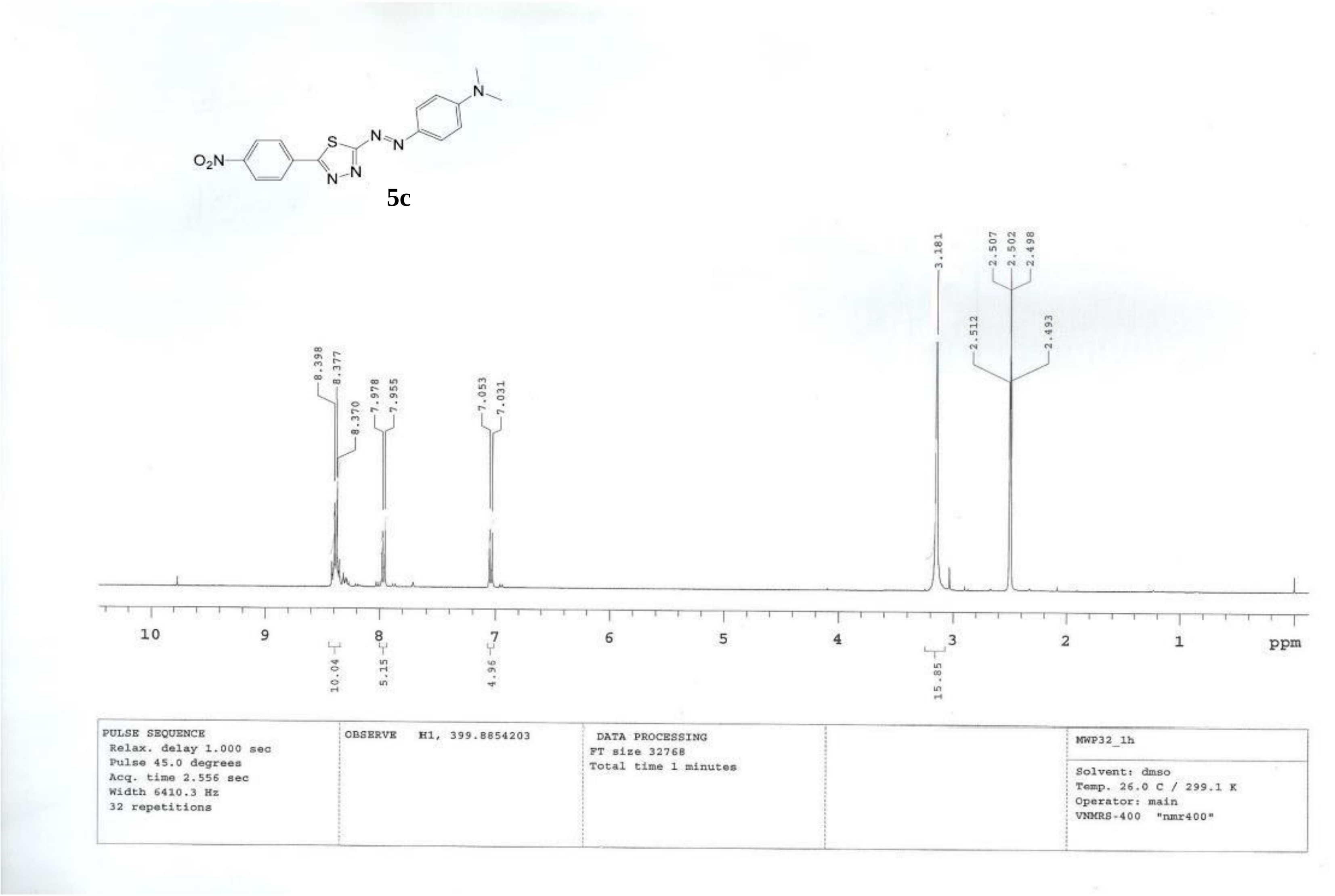

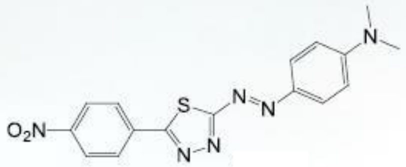

5c

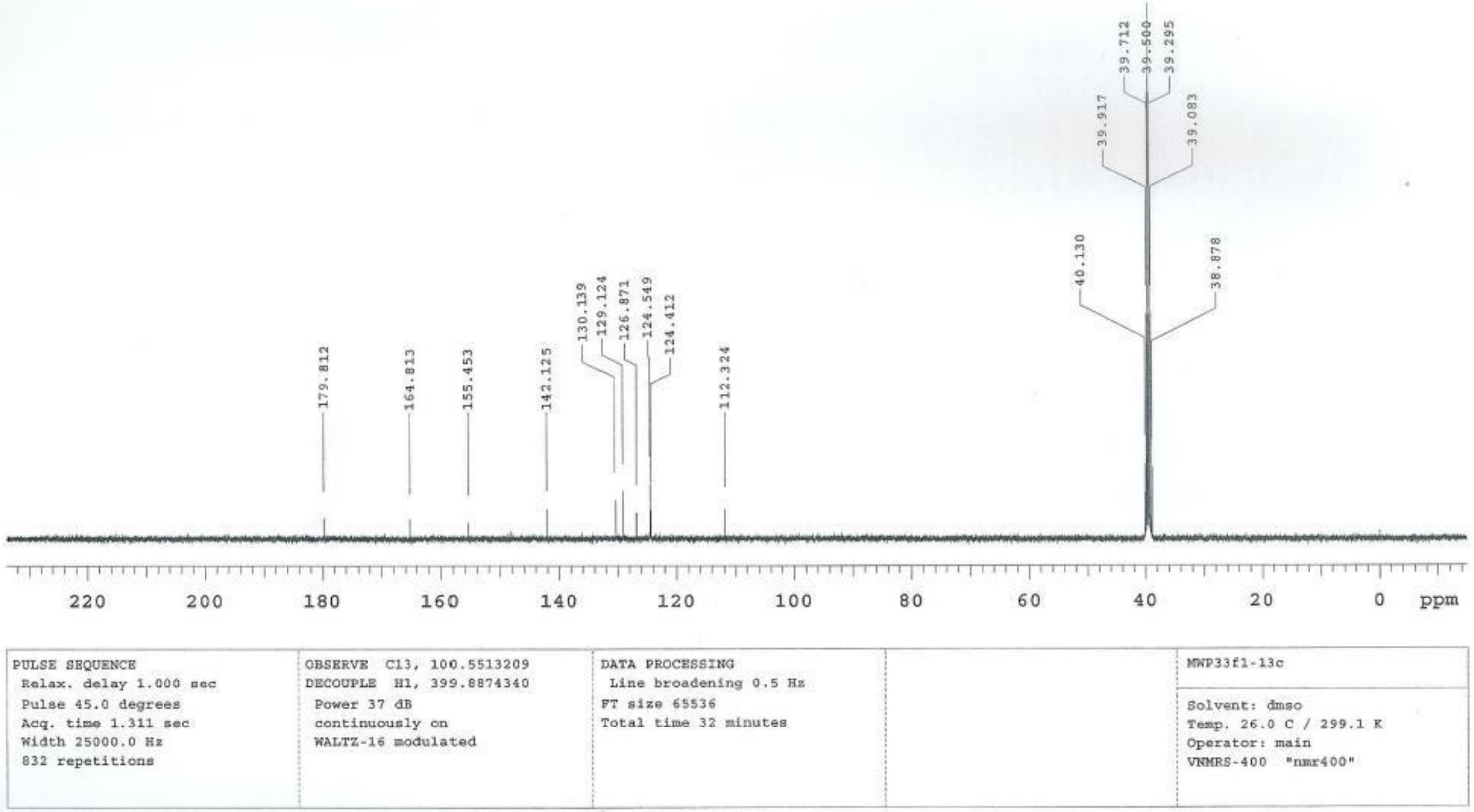

39.712
39.500
39.295
39.917
39.083
40.130
38.878
179.812
164.813
155.453
142.125
130.139
129.124
126.871
124.549
124.412
112.324
220 200 180 160 140 120 100 80 60 40 20 0 ppm
PULSE SEQUENCE
Relax. delay 1.000 sec
Pulse 45.0 degrees
Acq. time 1.311 sec
Width 25000.0 Hz
832 repetitions
OBSERVE C13, 100.5513209
DECOUPLE H1, 399.8874340
Power 37 dB
continuously on
WALTZ-16 modulated
DATA PROCESSING
Line broadening 0.5 Hz
FT size 65536
Total time 32 minutes
MWP33f1-13c
Solvent: dmso
Temp. 26.0 C / 299.1 K
Operator: main
VNMRS-400 "nmr400"

5-(4-Bromophenyl)-2-[4-(N,N-dimethylamino)phenylazo]-1,3,4-thiadiazole (**5d**)

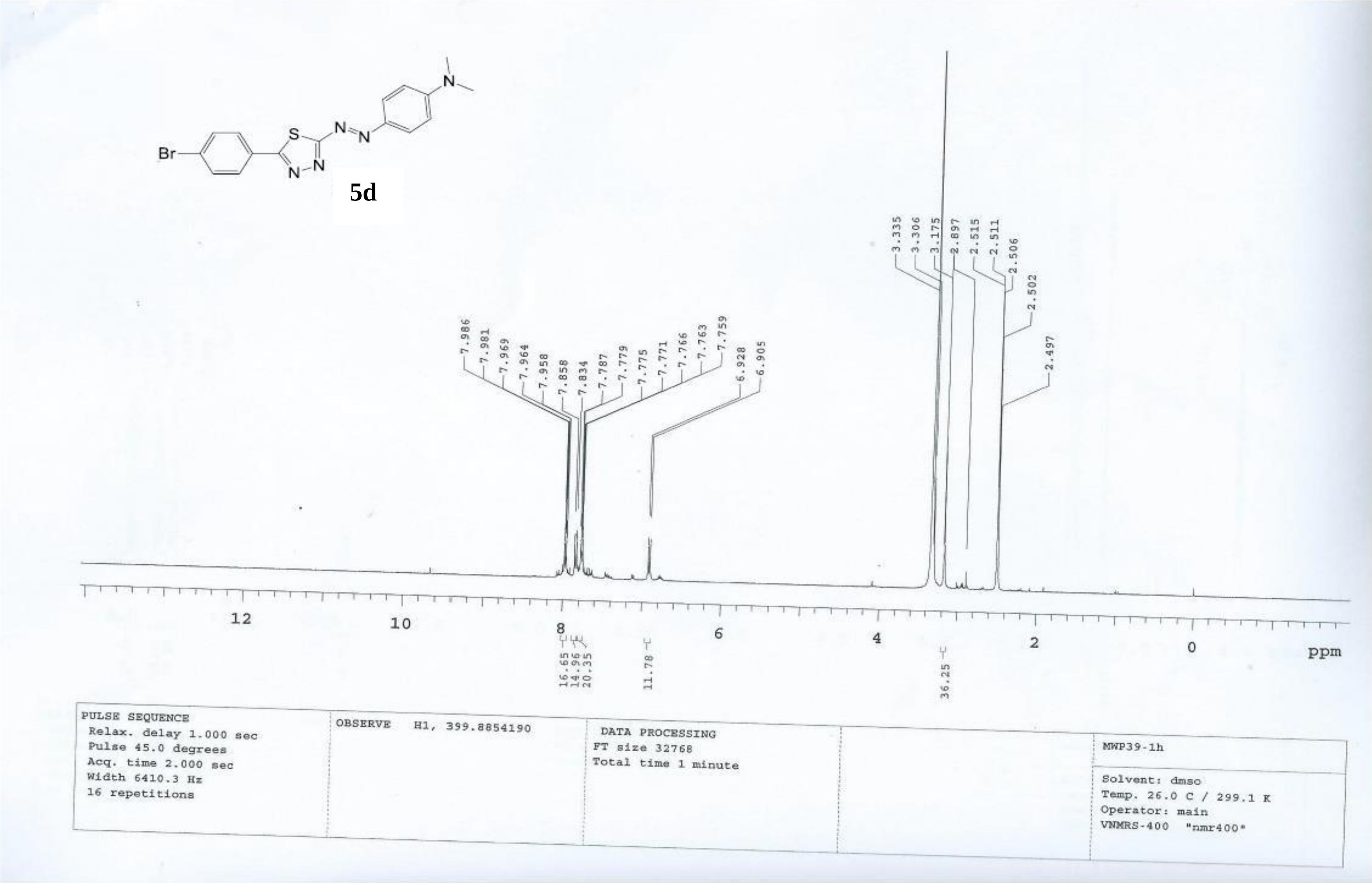

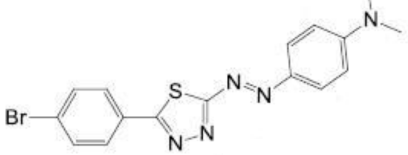

5d

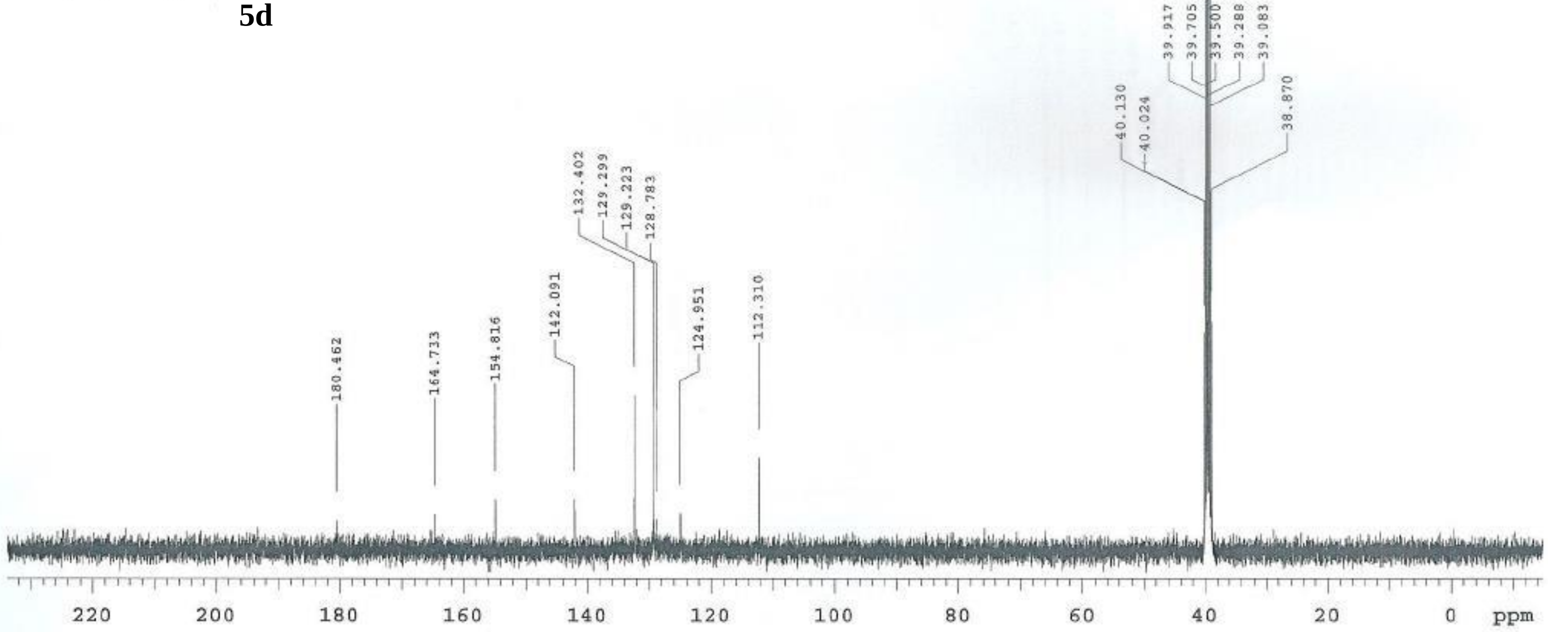

PULSE SEQUENCE	OBSERVE C13, 100.5513194	DATA PROCESSING		MWP39-13c
Relax. delay 1.000 sec<br>Pulse 45.0 degrees<br>Acq. time 1.311 sec<br>Width 25000.0 Hz<br>480 repetitions	DECOUPLE H1, 399.8874340<br>Power 37 dB<br>continuously on<br>WALTZ-16 modulated	Line broadening 0.5 Hz<br>FT size 65536<br>Total time 18 minutes		Solvent: dmso<br>Temp. 26.0 C / 299.1 K<br>Operator: main<br>VNMRS-400 "nmr400"

5-(4-*t*-Butylphenyl)-2-[4-(*N*,*N*-dimethylamino)phenylazo]-1,3,4-thiadiazole (**5e**)

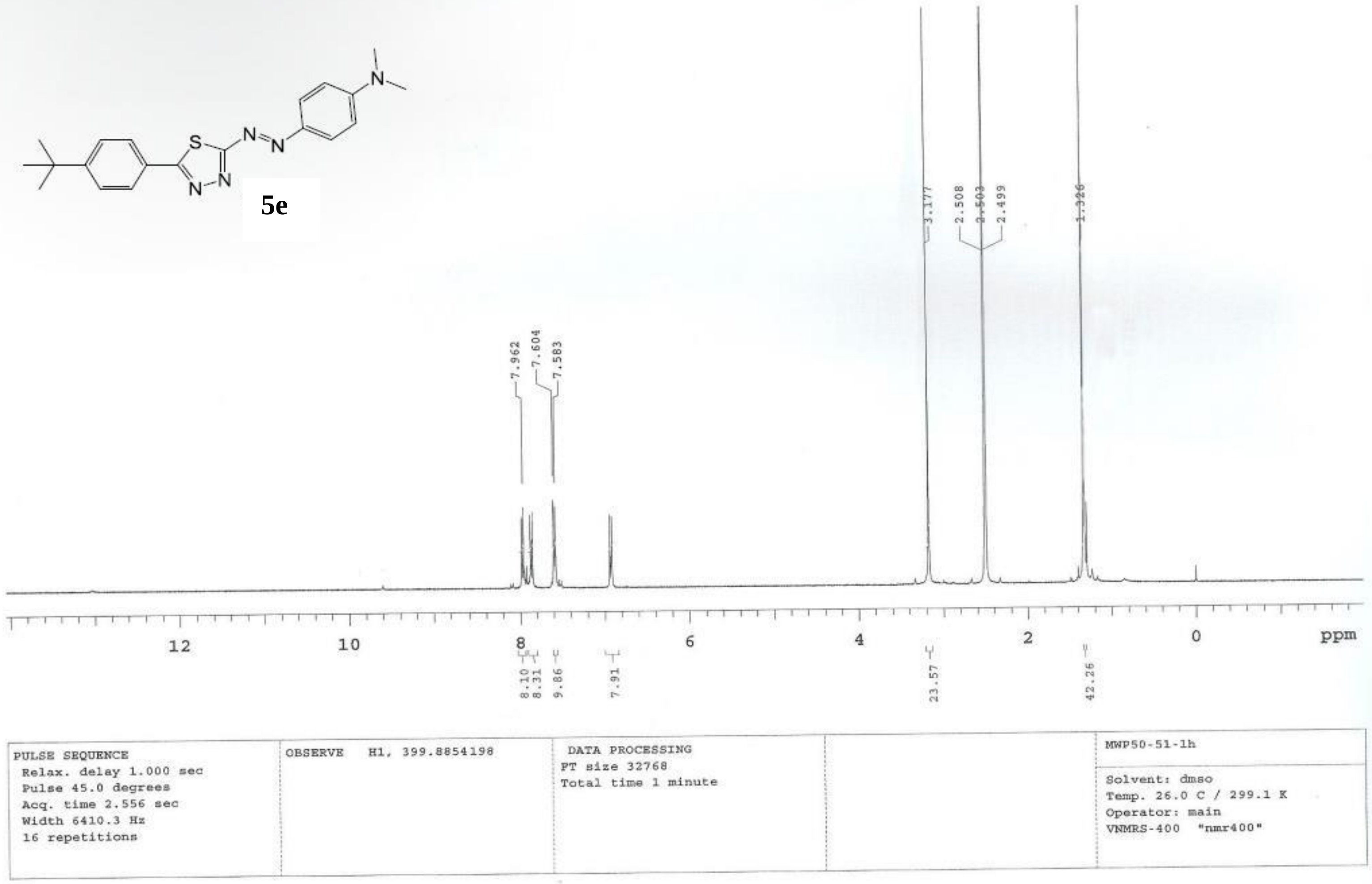

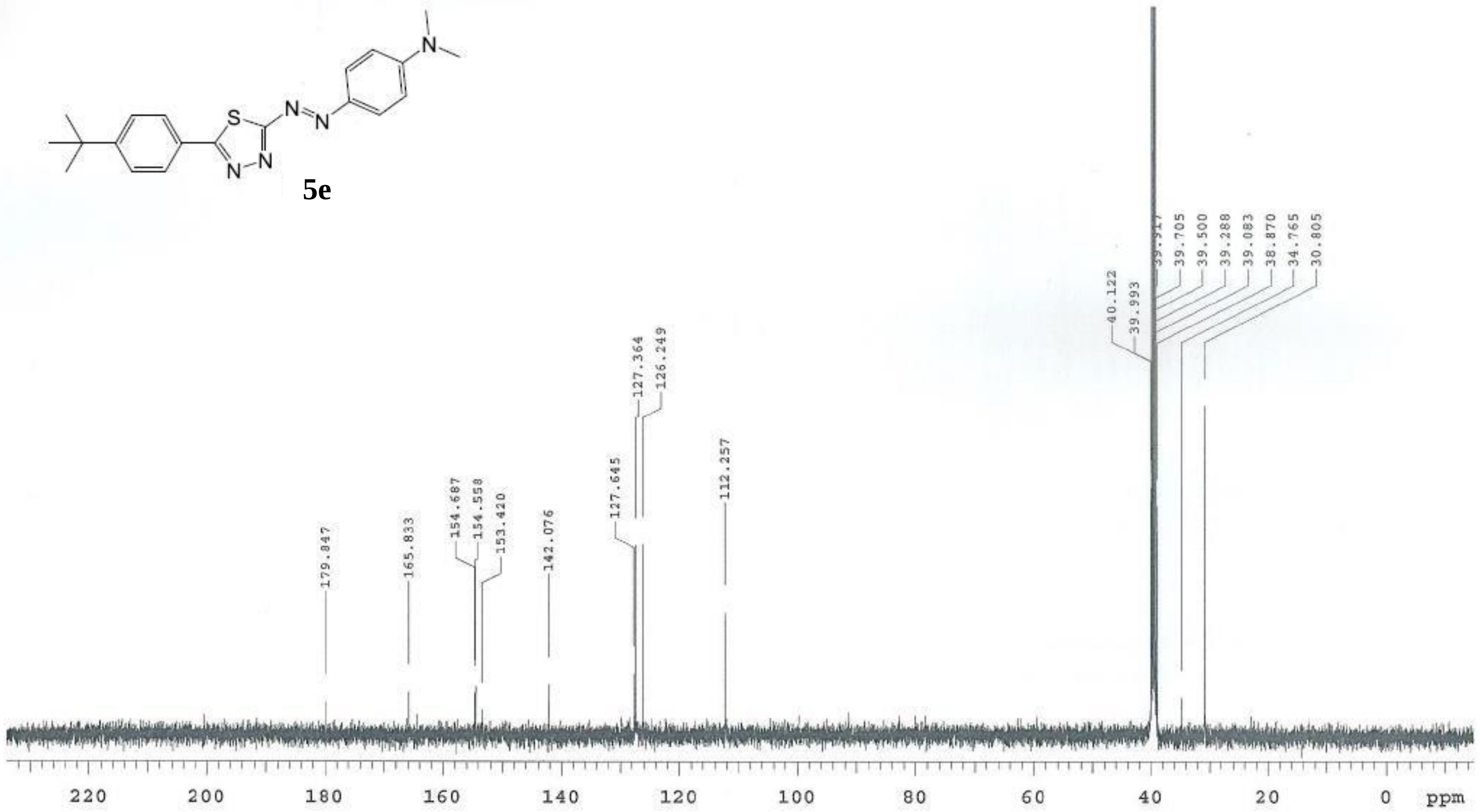

PULSE SEQUENCE	OBSERVE C13, 100.5513202	DATA PROCESSING		MWF50-51-13c
Relax. delay 1.000 sec	DECOUPLE H1, 399.8874340	Line broadening 0.5 Hz		Solvent: dmso
Pulse 45.0 degrees	Power 37 dB	FT size 65536		Temp. 26.0 C / 299.1 K
Acq. time 0.880 sec	continuously on	Total time 46 minutes		Operator: main
Width 25000.0 Hz	WALTZ-16 modulated			VNMRS-400 "nmr400"
1472 repetitions				

2-(4-Hydroxyphenylazo)-5-phenyl-1,3,4-thiadiazole (**6a**)

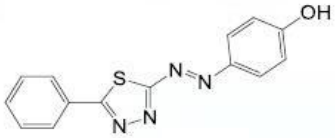

6a

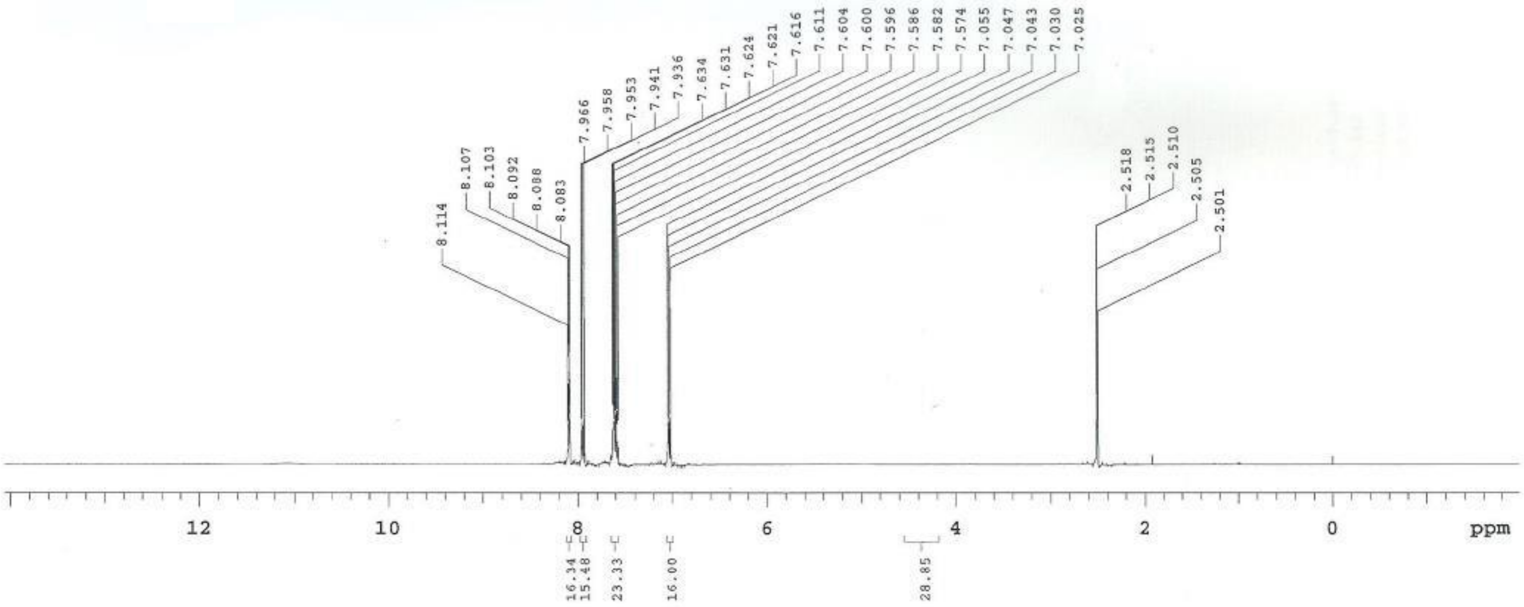

PULSE SEQUENCE	OBSERVE H1, 399.8854174	DATA PROCESSING		MWP13-1h
Relax. delay 1.000 sec<br>Pulse 45.0 degrees<br>Acq. time 2.000 sec<br>Width 6410.3 Hz<br>16 repetitions		FT size 32768<br>Total time 1 minute		Solvent: dmso<br>Temp. 26.0 C / 299.1 K<br>Operator: main<br>VNMRS-400 "nmr400"

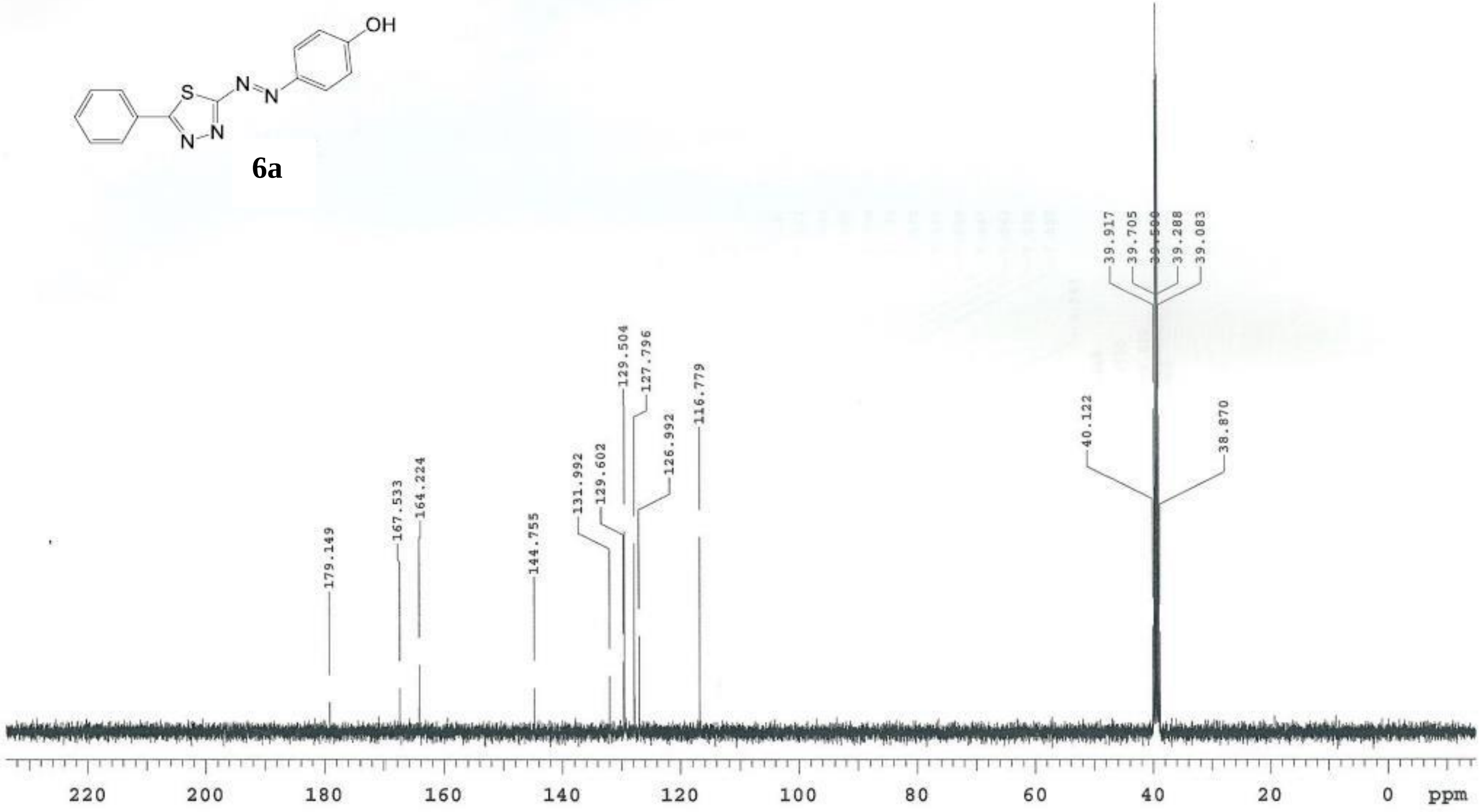

PULSE SEQUENCE
Relax. delay 2.000 sec
Pulse 45.0 degrees
Acq. time 1.311 sec
Width 25000.0 Hz
96 repetitions

OBSERVE C13, 100.5513202
DECOUPLE H1, 399.8874340
Power 37 dB
continuously on
WALTZ-16 modulated

DATA PROCESSING
Line broadening 0.5 Hz
FT size 65536
Total time 5 minutes

MWP13-13c

Solvent: dmso
Temp. 26.0 C / 299.1 K
Operator: main
VNMRS-400 "nmr400"

2-(4-Hydroxyphenylazo)-5-(4-methoxyphenyl)-1,3,4-thiadiazole (**6b**)

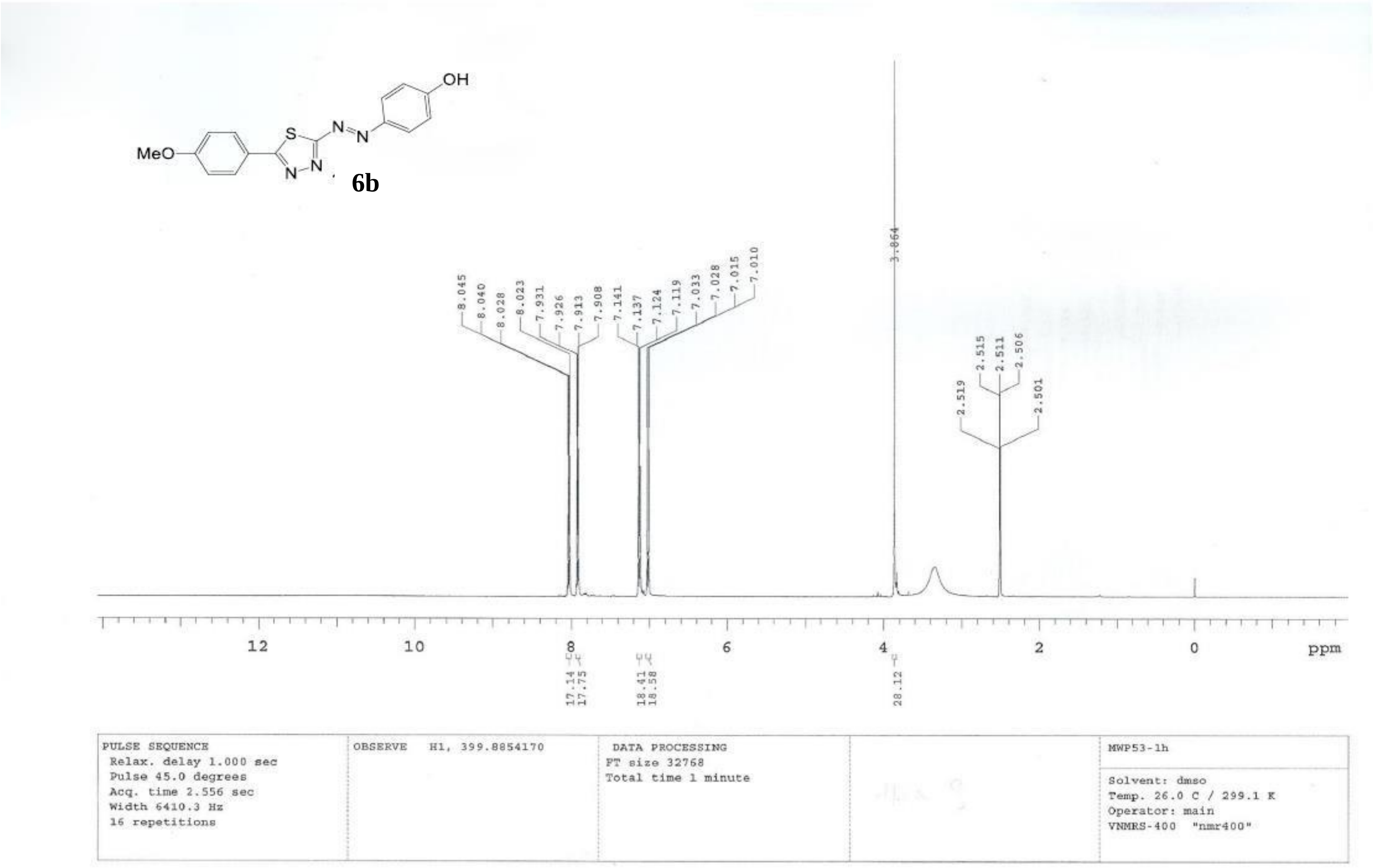

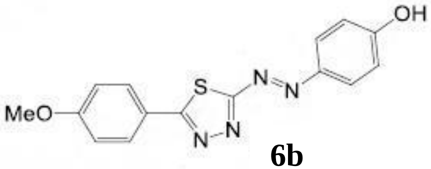

6b

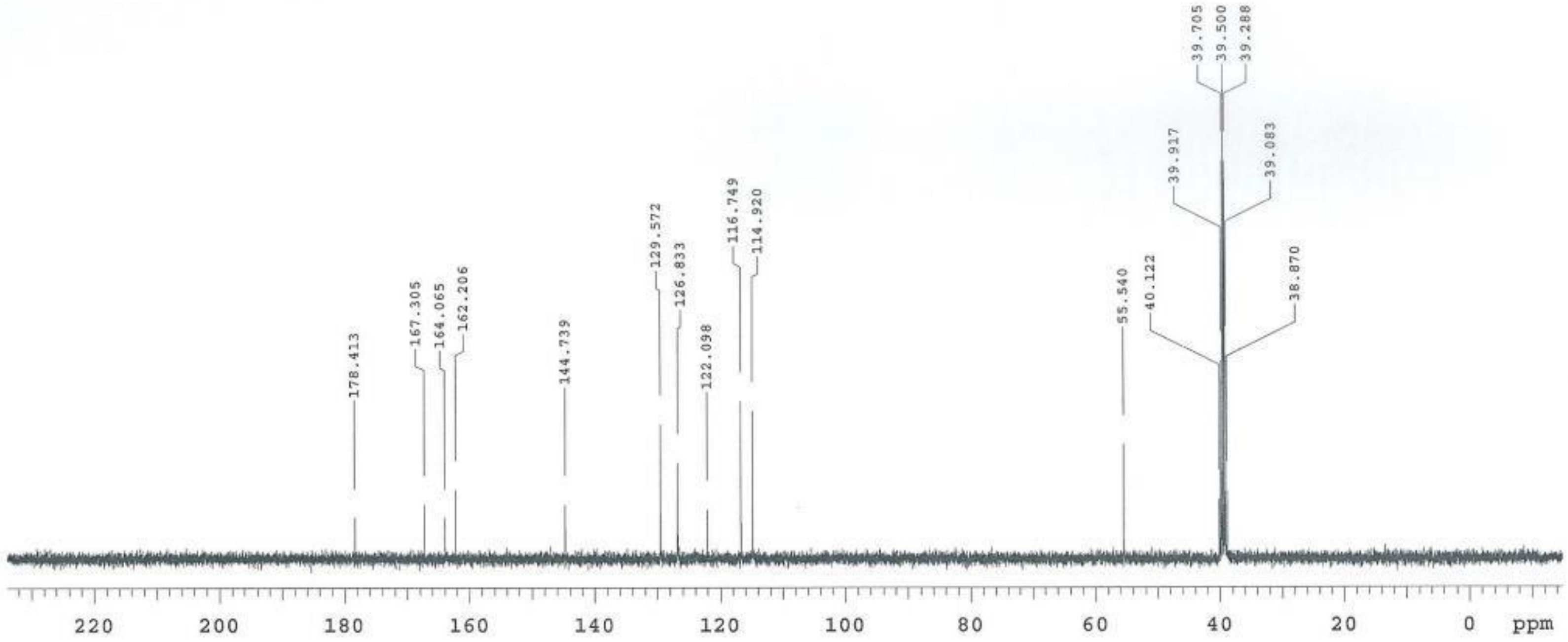

PULSE SEQUENCE	OBSERVE C13, 100.5513194	DATA PROCESSING		MWP53-13c
Relax. delay 1.000 sec	DECOUPLE H1, 399.8874340	Line broadening 0.5 Hz		Solvent: dmso
Pulse 45.0 degrees	Power 37 dB	FT size 65536		Temp. 26.0 C / 299.1 K
Acq. time 1.311 sec	continuously on	Total time 4 minutes		Operator: main
Width 25000.0 Hz	WALTZ-16 modulated			VNMRS-400 "nmr400"
128 repetitions				

2-(4-Hydroxyphenylazo)-5-(4-nitrophenyl)-1,3,4-thiadiazole (**6c**)

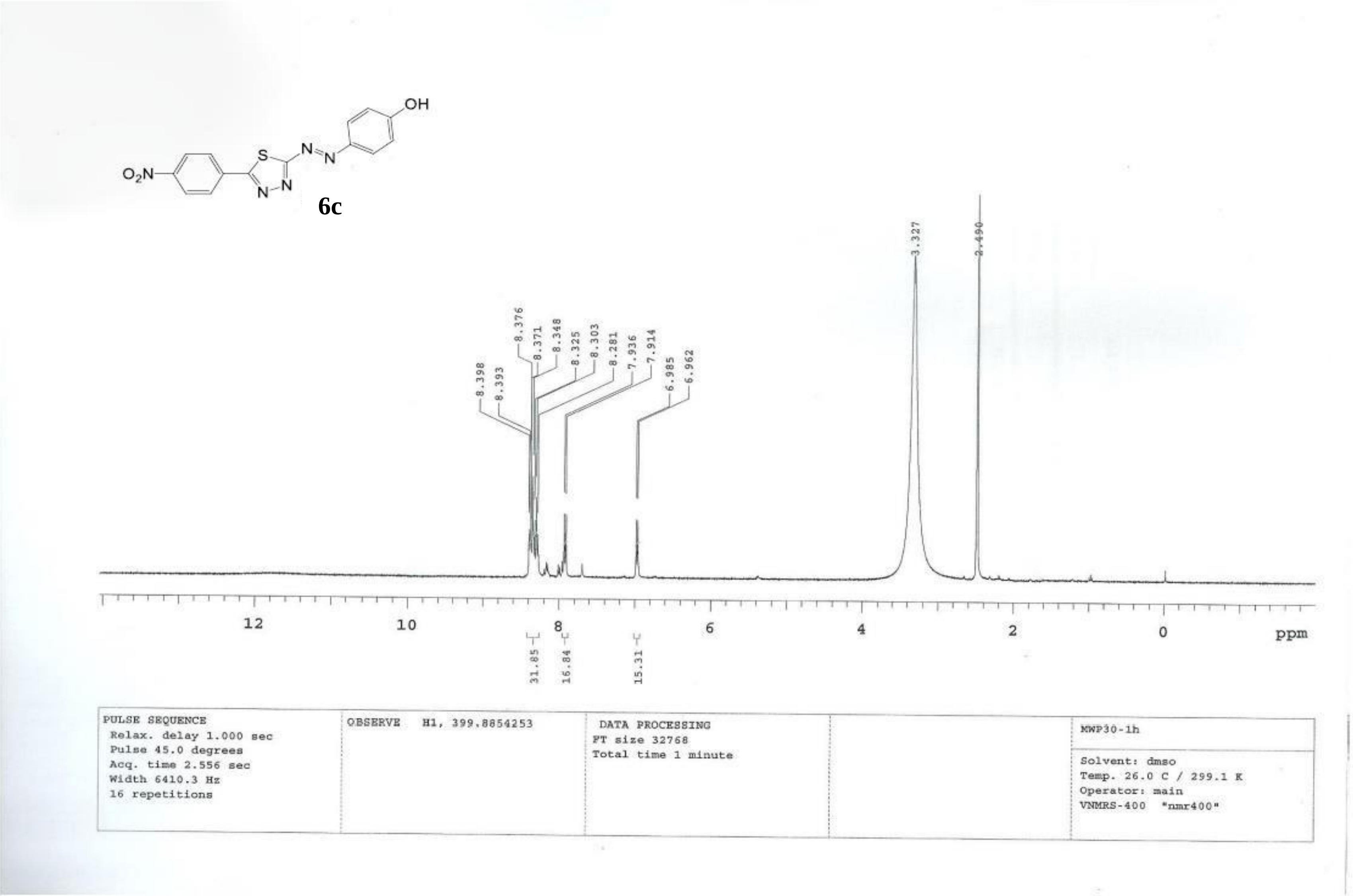

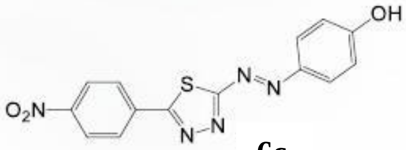

6c

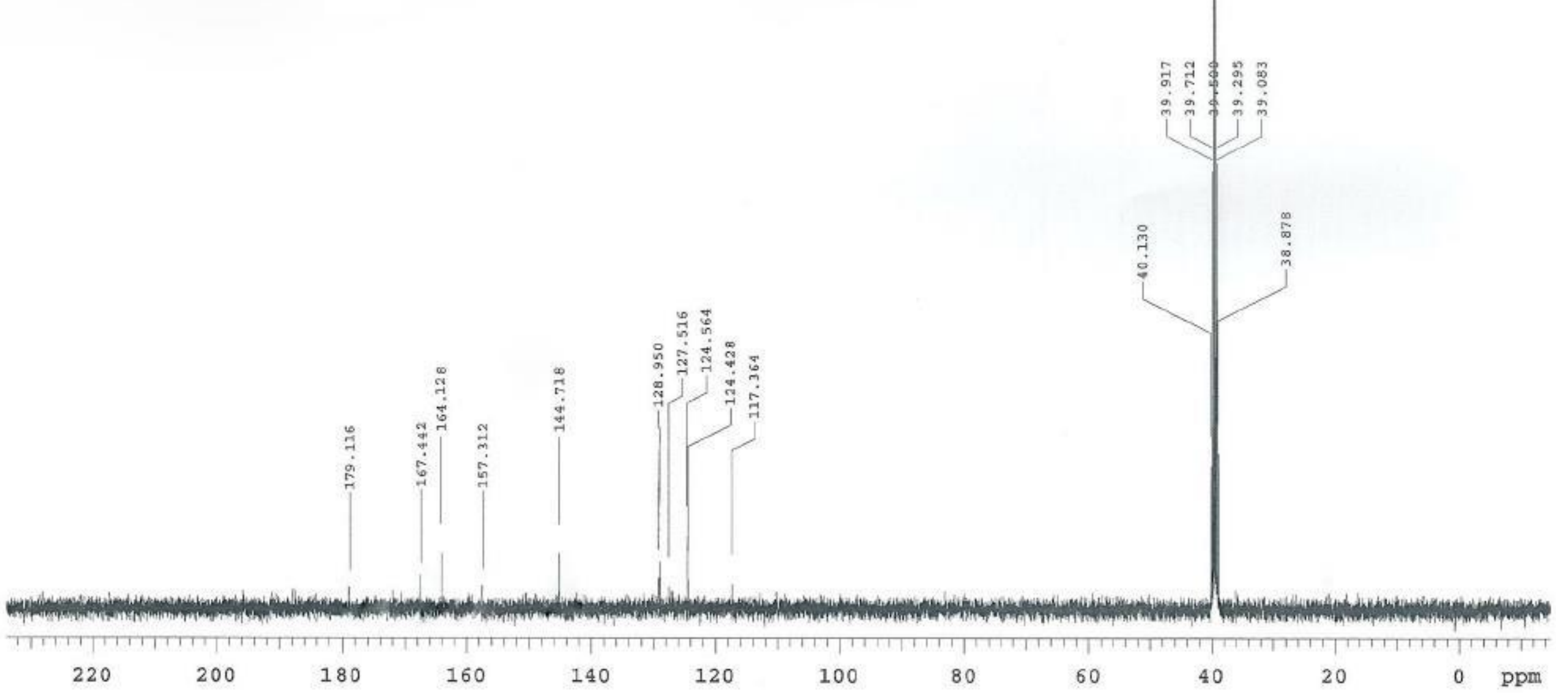

PULSE SEQUENCE
Relax. delay 1.000 sec
Pulse 45.0 degrees
Acq. time 1.311 sec
Width 25000.0 Hz
320 repetitions

OBSERVE C13, 100.5513186
DECOUPLE H1, 399.8874340
Power 37 dB
continuously on
WALTZ-16 modulated

DATA PROCESSING
Line broadening 0.5 Hz
FT size 65536
Total time 12 minutes

MWP30-13c

Solvent: dmso
Temp. 26.0 C / 299.1 K
Operator: main
VNMRS-400 "nmr400"

5-(4-Bromophenyl)-2-(4-hydroxyphenylazo)-1,3,4-thiadiazole (**6d**)

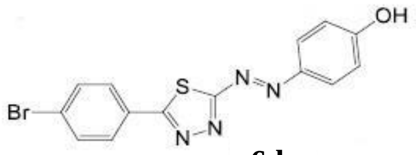

6d

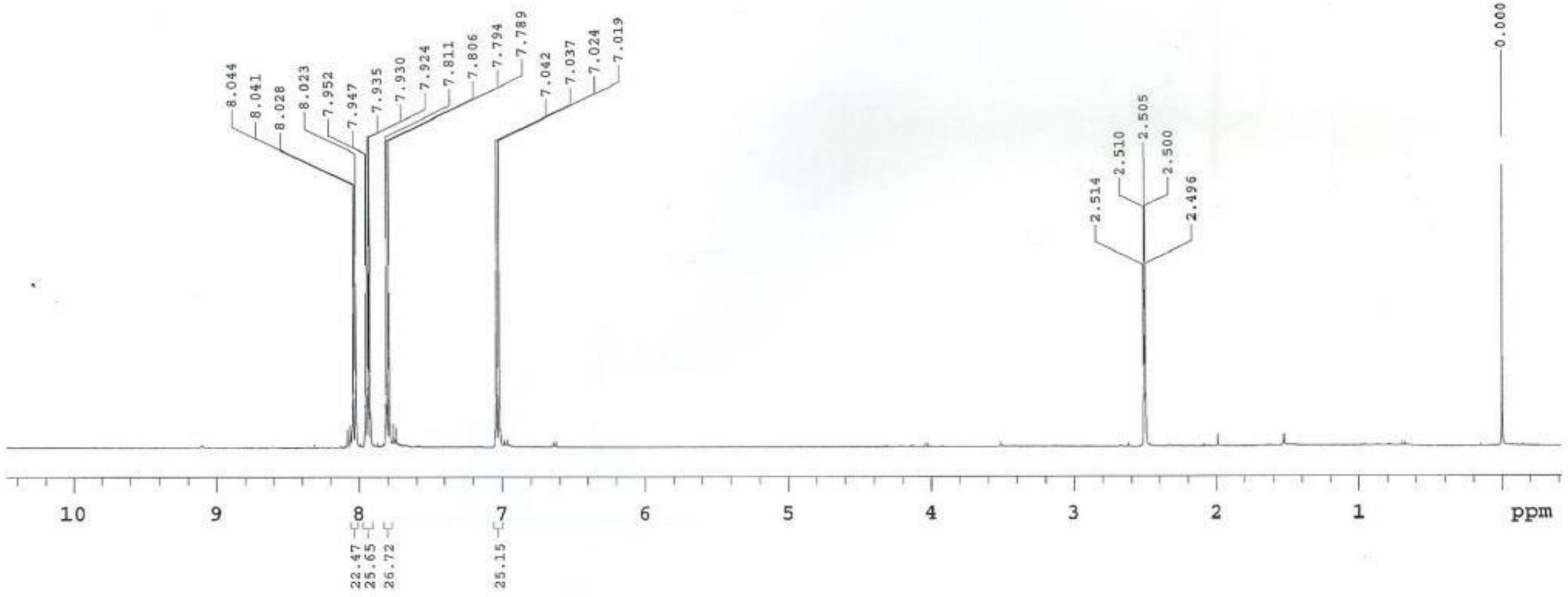

PULSE SEQUENCE<br>Relax. delay 1.000 sec<br>Pulse 90.0 degrees<br>Acq. time 2.556 sec<br>Width 6410.3 Hz<br>16 repetitions	OBSERVE H1, 399.8854194	DATA PROCESSING<br>FT size 32768<br>Total time 1 minute		KJp05_1h<br>Solvent: dmso<br>Temp. 26.0 C / 299.1 K<br>Operator: main<br>VNMRS-400 "nmr400"

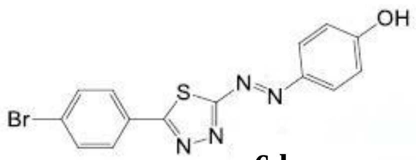

6d

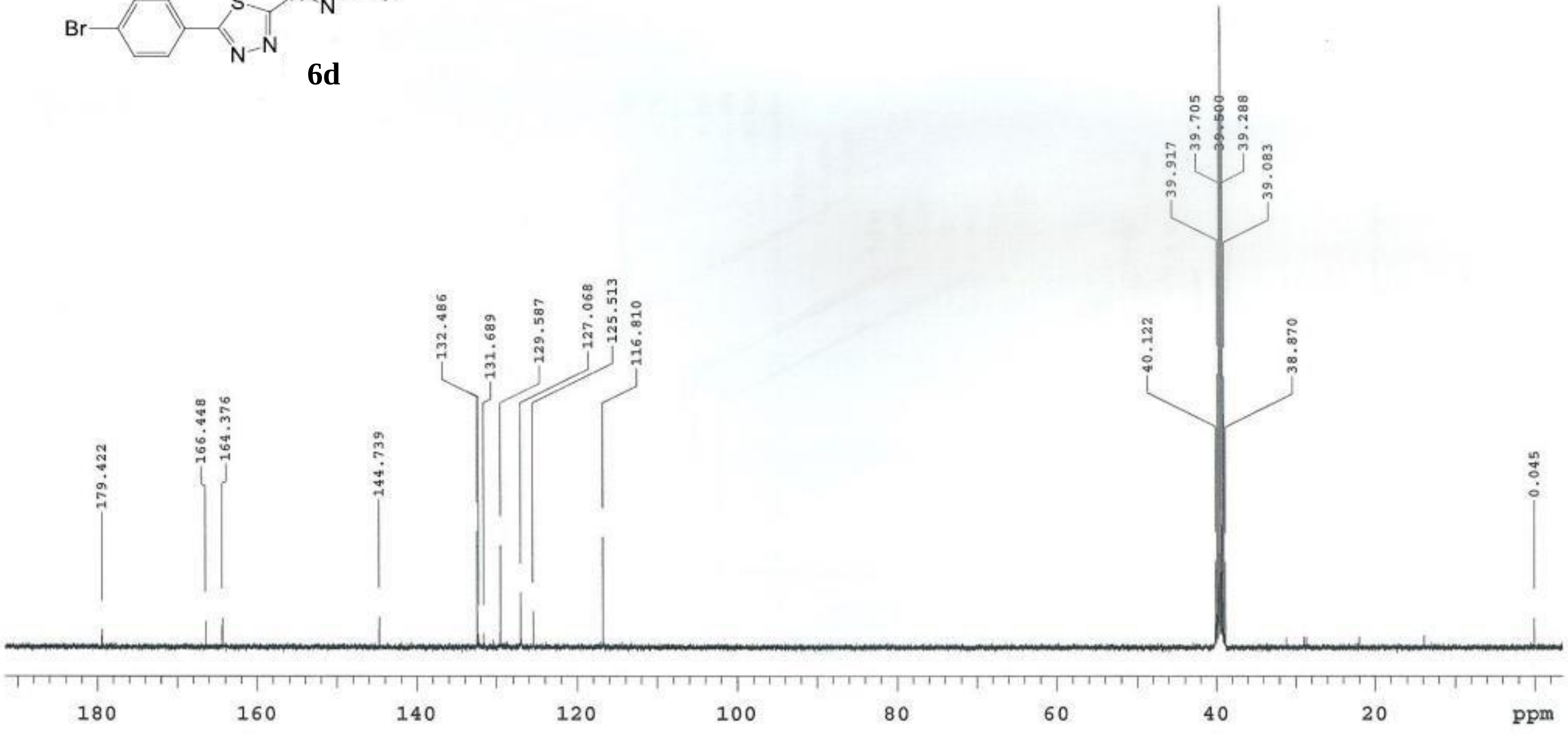

PULSE SEQUENCE<br>Relax. delay 1.000 sec<br>Pulse 45.0 degrees<br>Acq. time 1.311 sec<br>Width 25000.0 Hz<br>1808 repetitions	OBSERVE C13, 100.5513217<br>DECOUPLE H1, 399.8874340<br>Power 37 dB<br>continuously on<br>WALTZ-16 modulated	DATA PROCESSING<br>Line broadening 0.5 Hz<br>FT size 65536<br>Total time 69 minutes		KJp05_13c<br>Solvent: dmso<br>Temp. 26.0 C / 299.1 K<br>Operator: main<br>VNMRS-400 "nmr400"

5-(4-t-Butylphenyl)-2-(4-hydroxyphenylazo)-1,3,4-thiadiazole (**6e**)

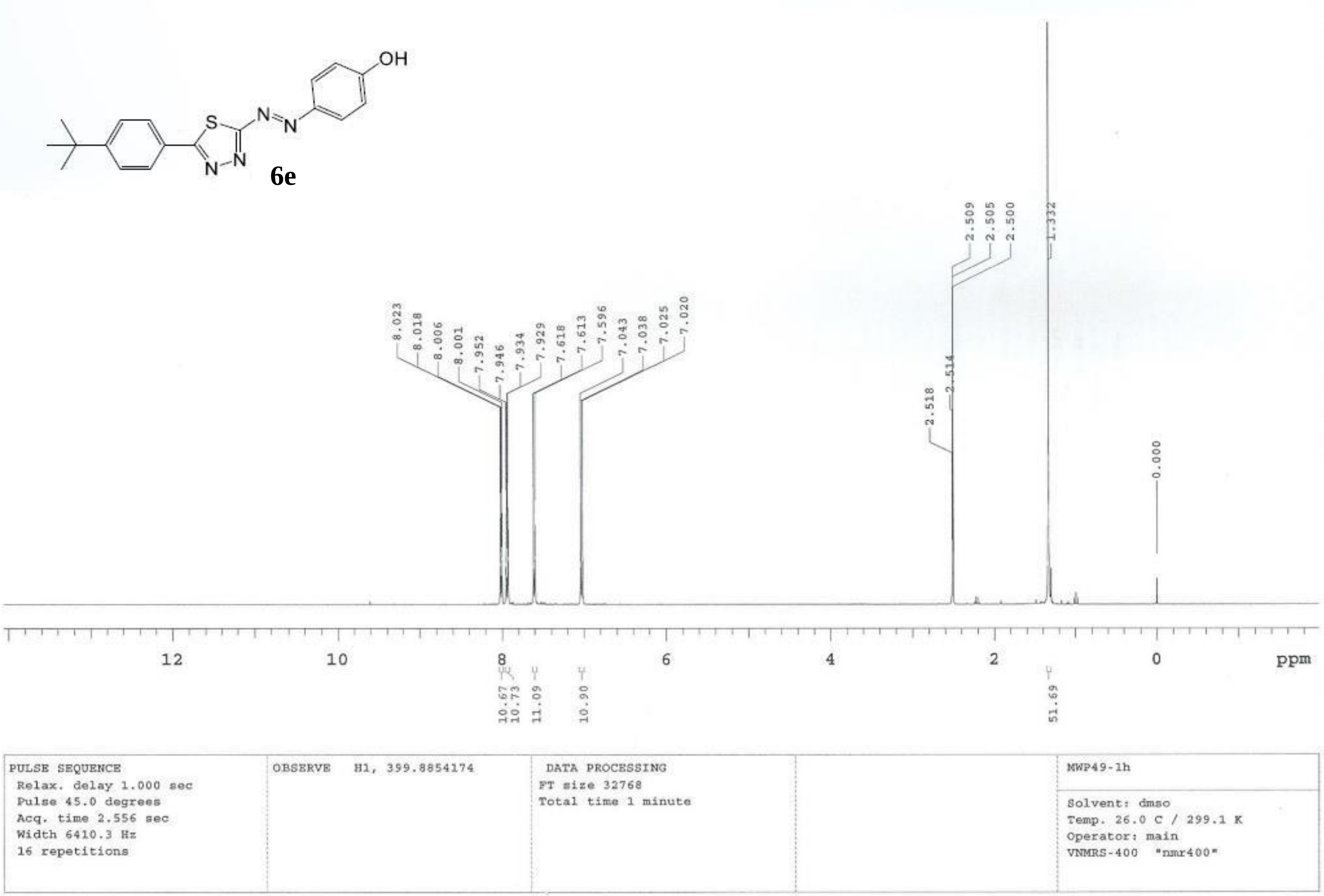

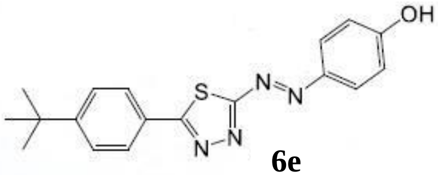

6e

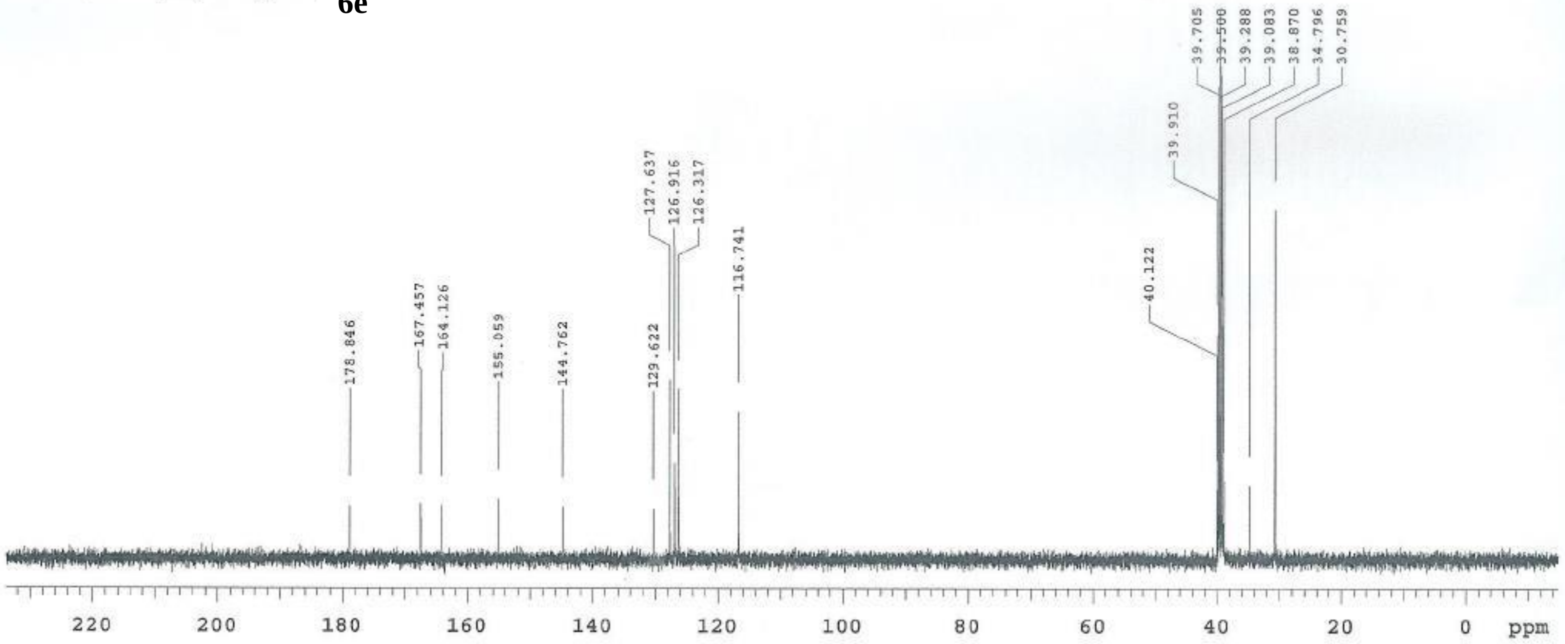

PULSE SEQUENCE<br>Relax. delay 1.000 sec<br>Pulse 45.0 degrees<br>Acq. time 1.311 sec<br>Width 25000.0 Hz<br>128 repetitions	OBSERVE C13, 100.5513209<br>DECOUPLE H1, 399.8874340<br>Power 37 dB<br>continuously on<br>WALTZ-16 modulated	DATA PROCESSING<br>Line broadening 0.5 Hz<br>FT size 65536<br>Total time 4 minutes		MWP49-13c<br>Solvent: dmso<br>Temp. 26.0 C / 299.1 K<br>Operator: main<br>VNMRS-400 "nmr400"

2. UV-Vis absorption data for compounds 4a-e, 5a-e, 6a-e

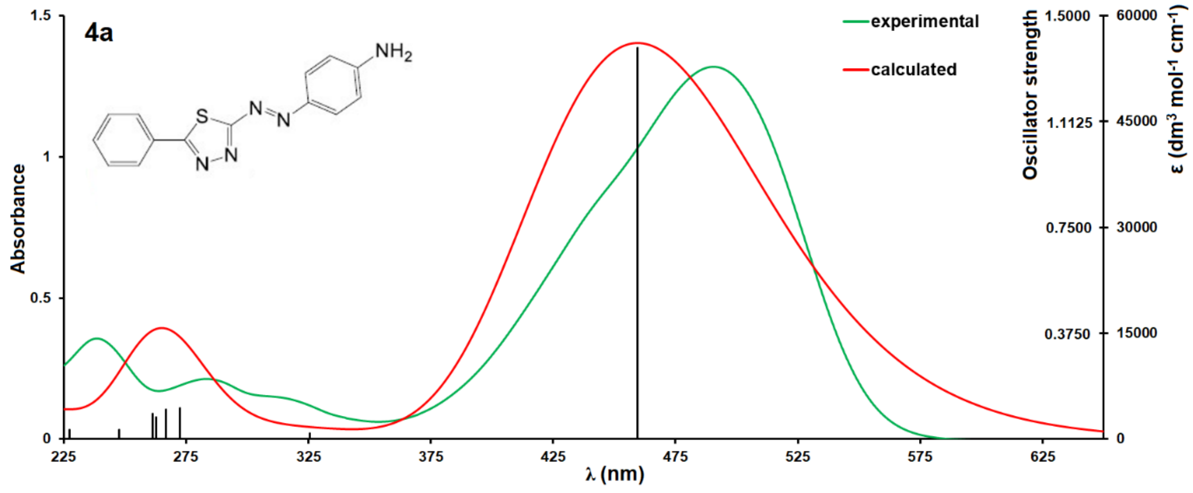

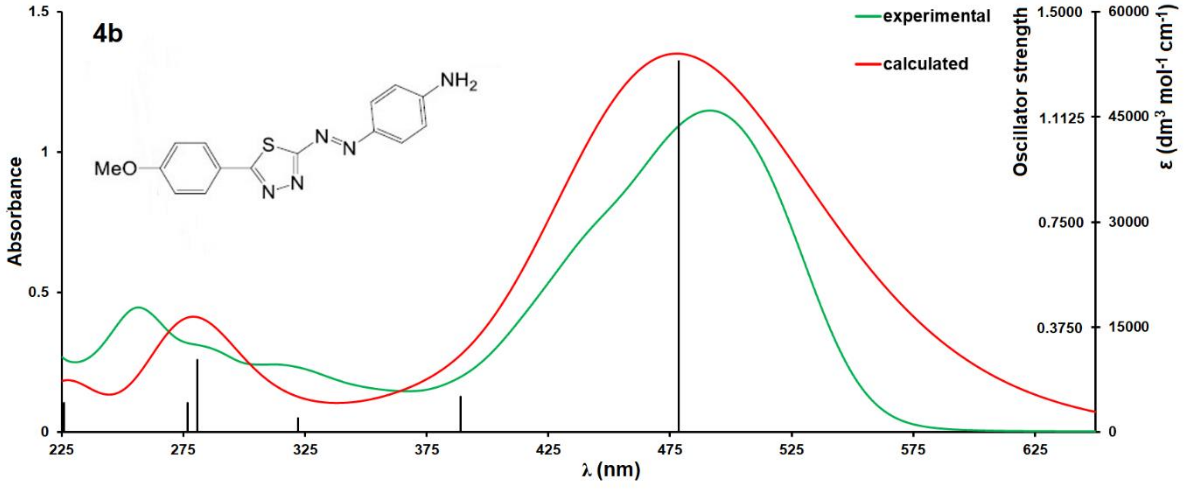

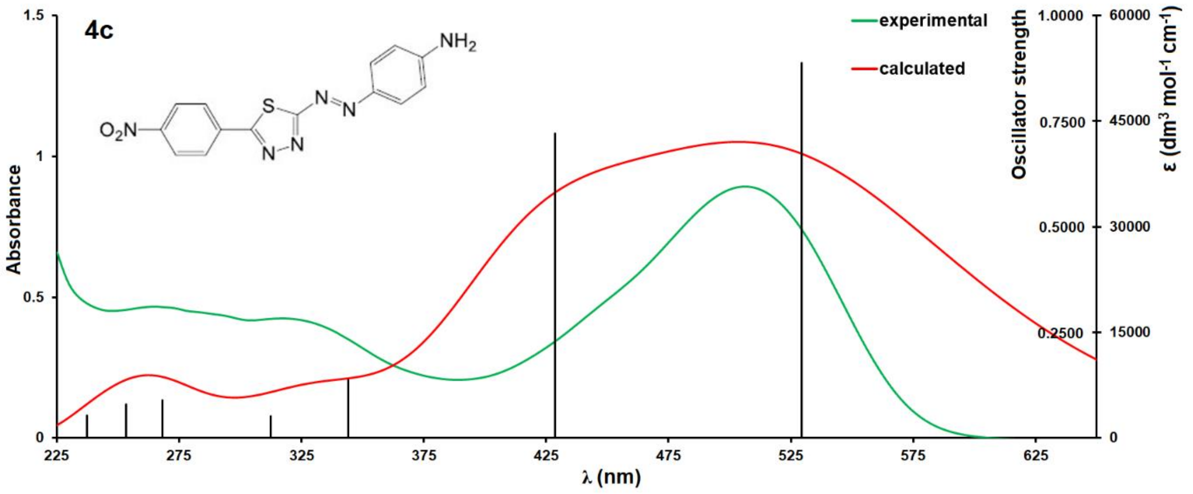

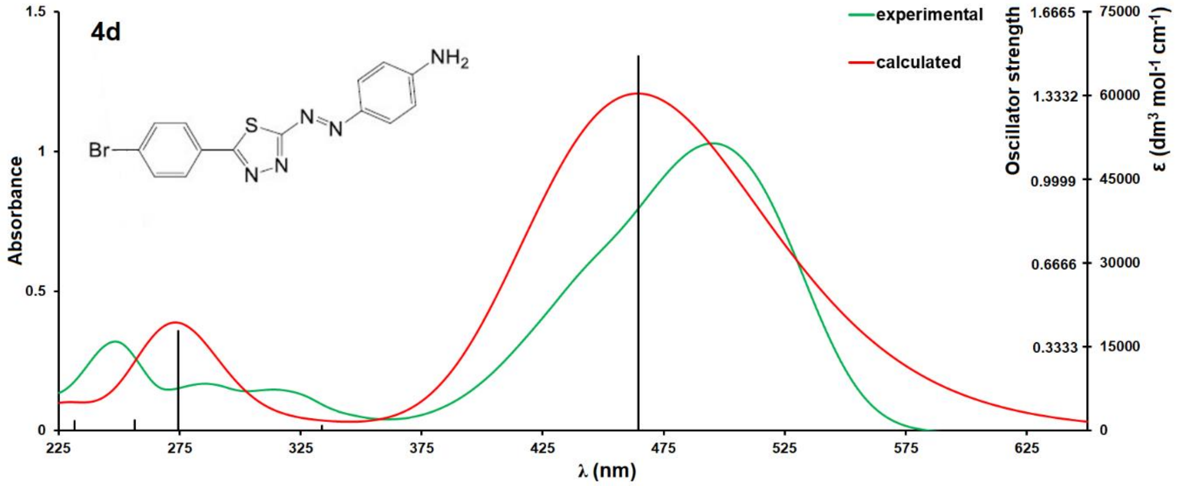
4d
experimental
calculated
Absorbance
Oscillator strength
ε (dm3 mol-1 cm-1)
λ (nm)
NH2
Br
S
N
N-N

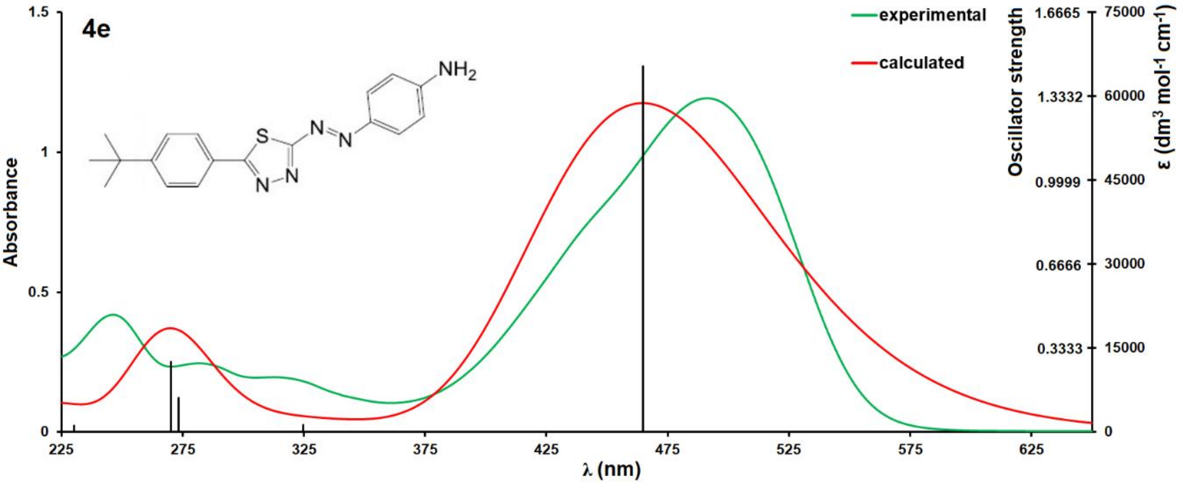
4e
experimental
calculated
Absorbance
Oscillator strength
ε (dm3 mol-1 cm-1)
λ (nm)
NH2
S
N
N-N

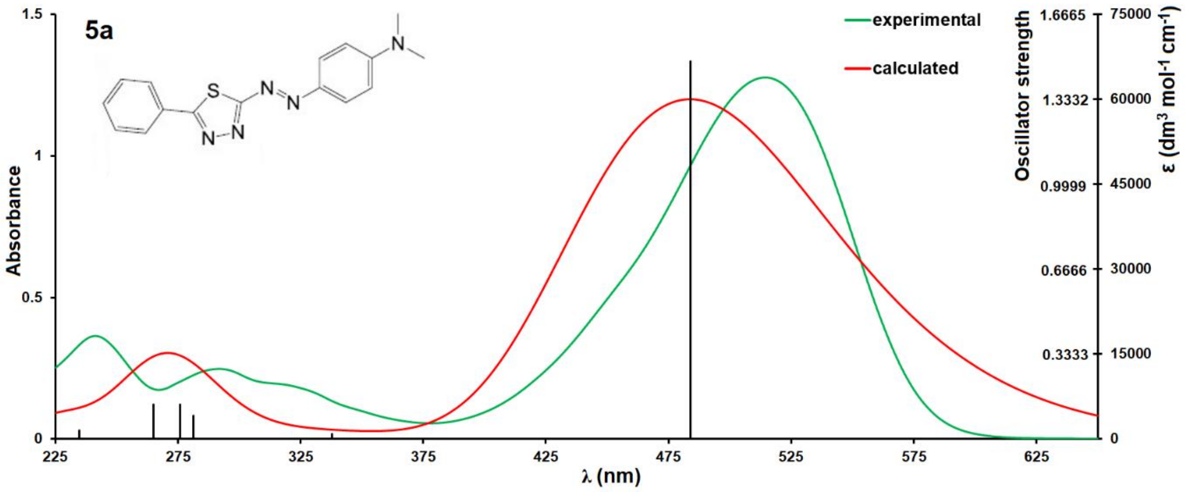
5a
experimental
calculated
Absorbance
Oscillator strength
ε (dm3 mol-1 cm-1)
λ (nm)
N
S
N-N

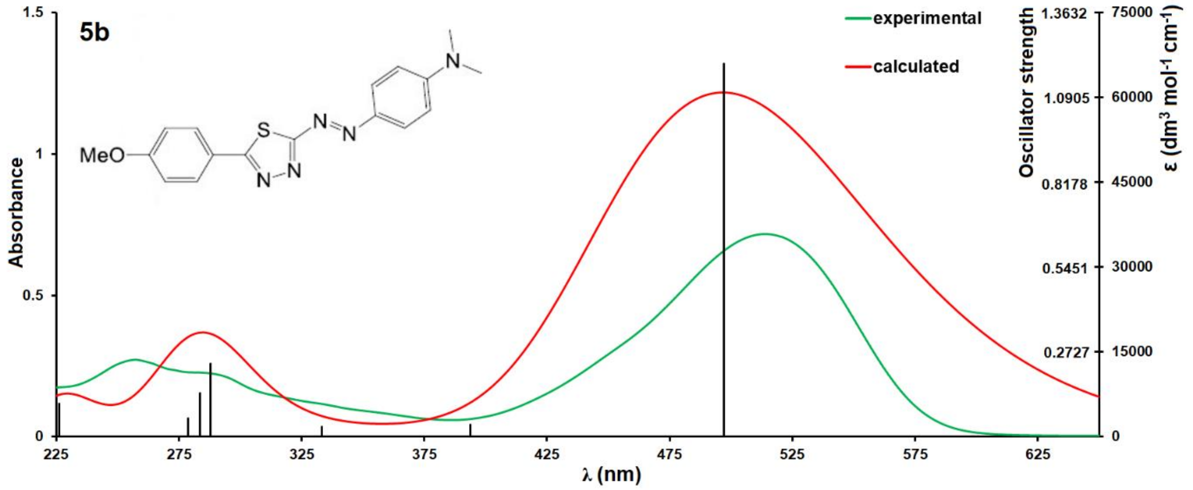
5b
MeO
S
N
N
N
N
N
experimental
calculated
Absorbance
Oscillator strength
ε (dm3 mol-1 cm-1)
λ (nm)
1.5
1
0.5
0
225
275
325
375
425
475
525
575
625
1.3632
1.0905
0.8178
0.5451
0.2727
75000
60000
45000
30000
15000

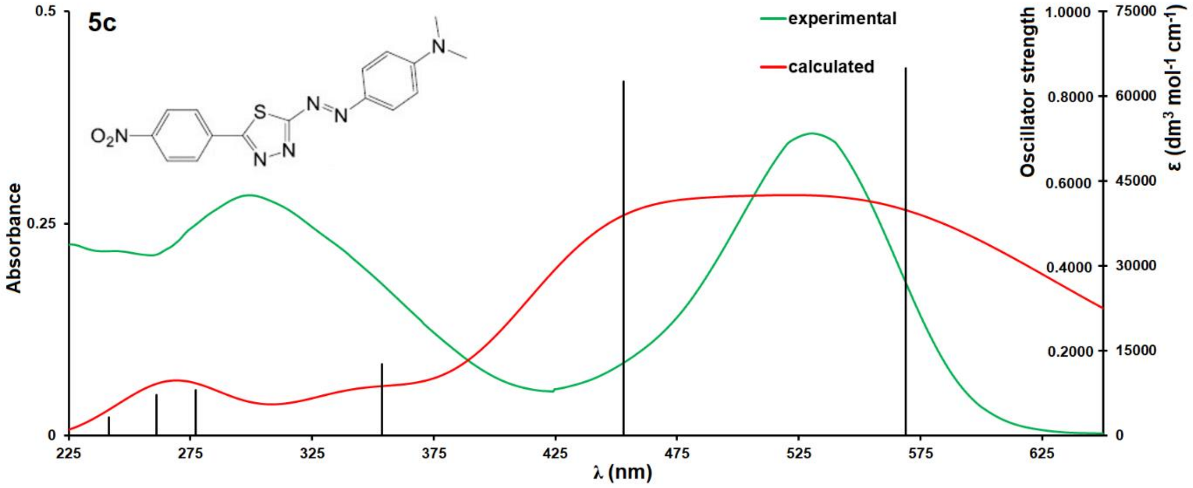
5c
O2N
S
N
N
N
N
N
experimental
calculated
Absorbance
Oscillator strength
ε (dm3 mol-1 cm-1)
λ (nm)
0.5
0.25
0
225
275
325
375
425
475
525
575
625
1.0000
0.8000
0.6000
0.4000
0.2000
75000
60000
45000
30000
15000

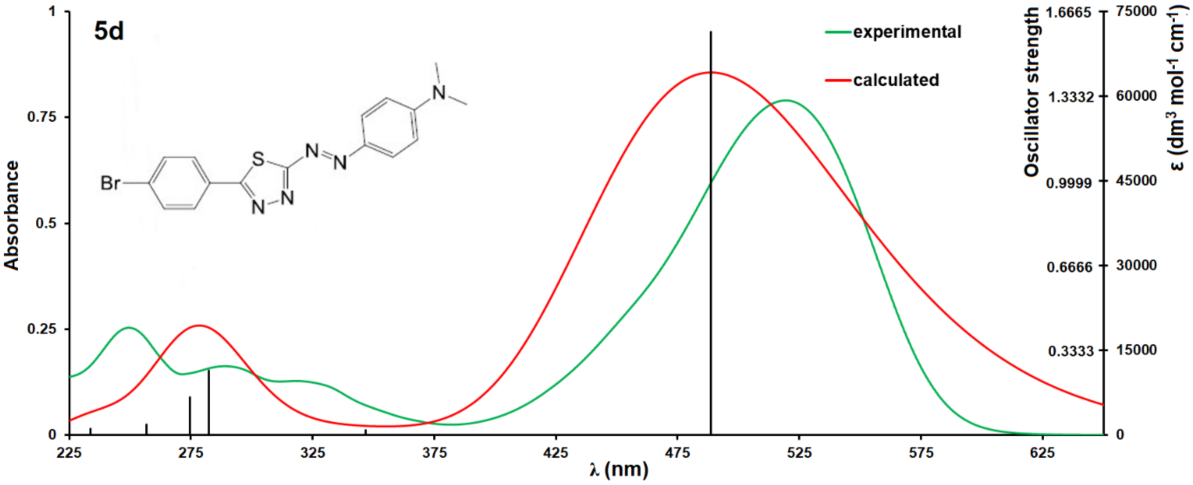
5d
Br
S
N
N
N
N
N
experimental
calculated
Absorbance
Oscillator strength
ε (dm3 mol-1 cm-1)
λ (nm)
1
0.75
0.5
0.25
0
225
275
325
375
425
475
525
575
625
1.6665
1.3332
0.9999
0.6666
0.3333
75000
60000
45000
30000
15000

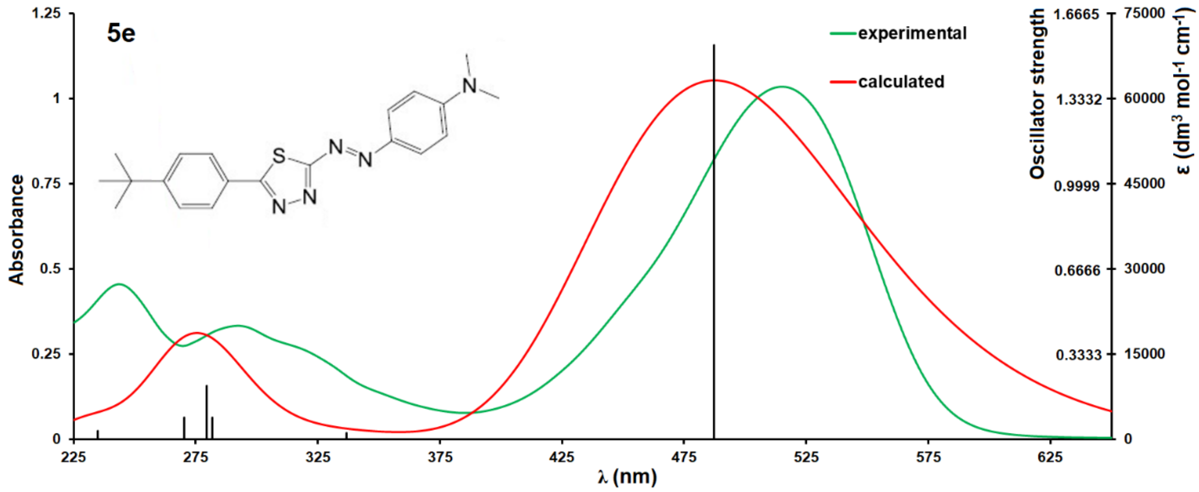
5e
experimental
calculated
Absorbance
Oscillator strength
ε (dm3 mol-1 cm-1)
λ (nm)
S
N
N
N
N
N
0 0.25 0.5 0.75 1 1.25
225 275 325 375 425 475 525 575 625
0 0.3333 0.6666 0.9999 1.3332 1.6665
0 15000 30000 45000 60000 75000

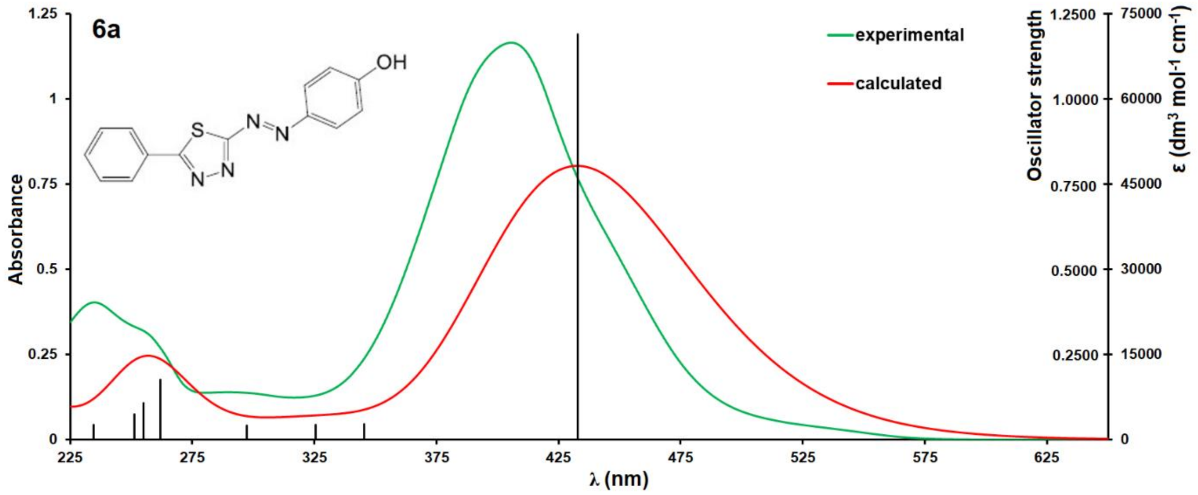
6a
experimental
calculated
Absorbance
Oscillator strength
ε (dm3 mol-1 cm-1)
λ (nm)
OH
S
N
N
N
N
0 0.25 0.5 0.75 1 1.25
225 275 325 375 425 475 525 575 625
0 0.2500 0.5000 0.7500 1.0000 1.2500
0 15000 30000 45000 60000 75000

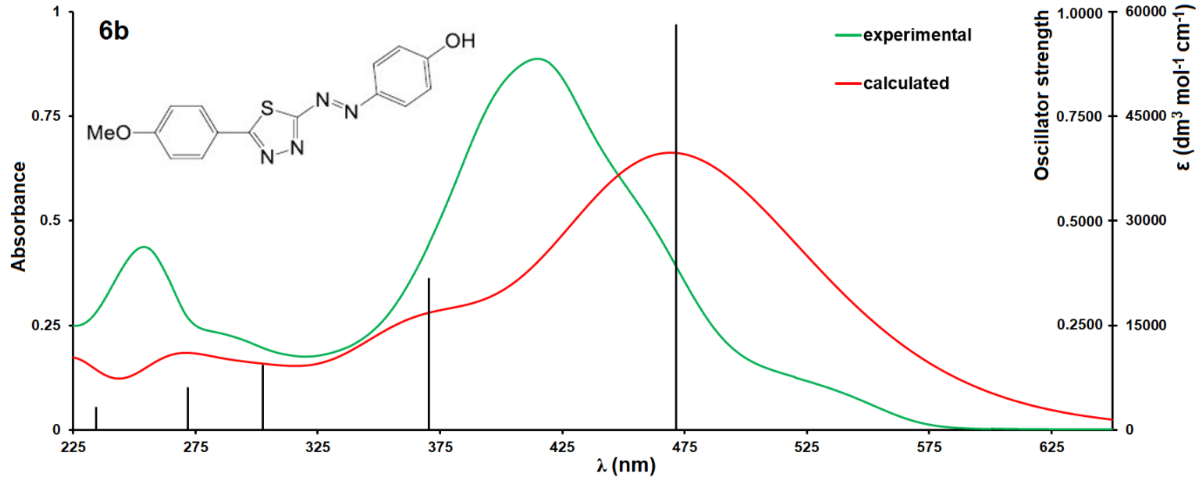
6b
experimental
calculated
Absorbance
Oscillator strength
ε (dm3 mol-1 cm-1)
λ (nm)
OH
MeO
S
N
N
N
N
0 0.25 0.5 0.75 1
225 275 325 375 425 475 525 575 625
0 0.2500 0.5000 0.7500 1.0000
0 15000 30000 45000 60000

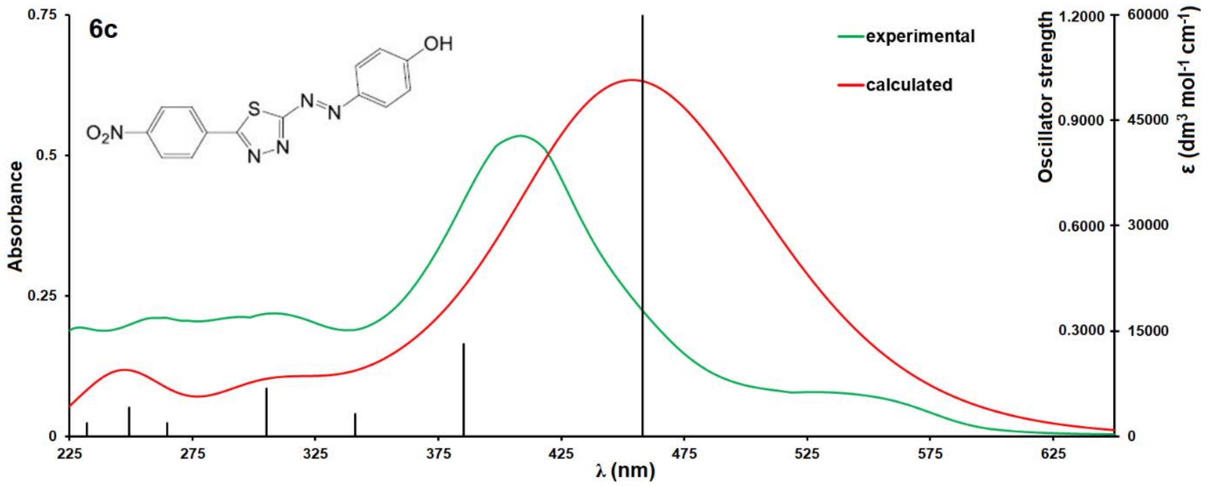
6c
OH
O_2N
S
N
N
N
N
experimental
calculated
Absorbance
0.75
0.5
0.25
0
225
275
325
375
425
475
525
575
625
λ (nm)
Oscillator strength
1.2000
0.9000
0.6000
0.3000
ε (dm³ mol⁻¹ cm⁻¹)
60000
45000
30000
15000

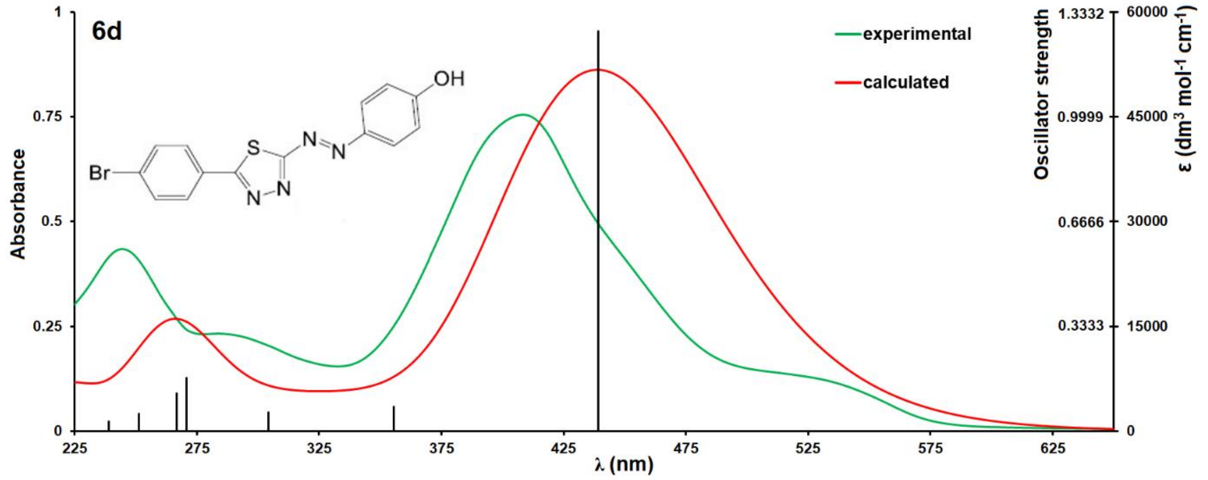
6d
OH
Br
S
N
N
N
N
experimental
calculated
Absorbance
1
0.75
0.5
0.25
0
225
275
325
375
425
475
525
575
625
λ (nm)
Oscillator strength
1.3332
0.9999
0.6666
0.3333
ε (dm³ mol⁻¹ cm⁻¹)
60000
45000
30000
15000

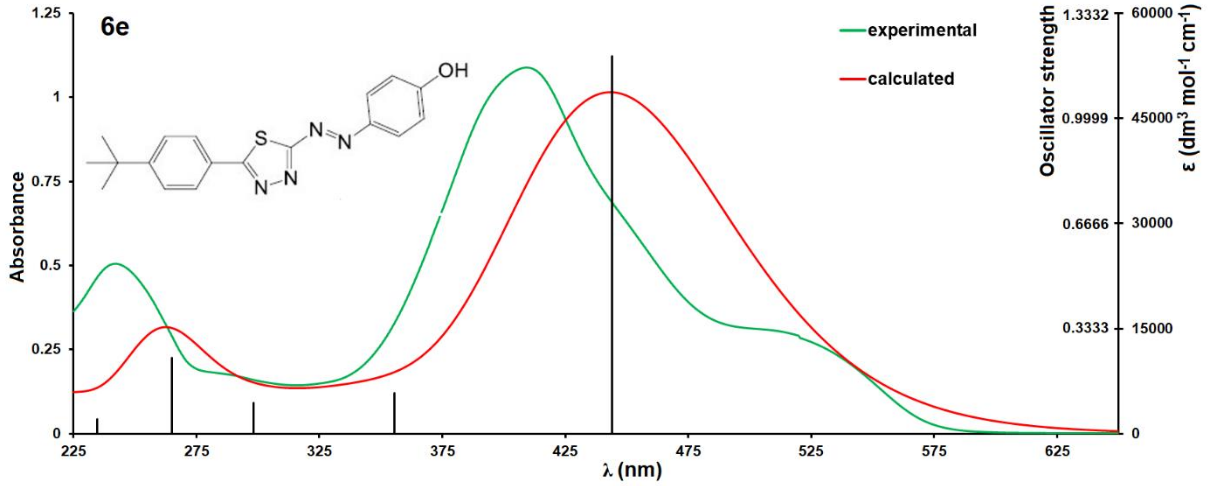
6e
OH
S
N
N
N
N
experimental
calculated
Absorbance
1.25
1
0.75
0.5
0.25
0
225
275
325
375
425
475
525
575
625
λ (nm)
Oscillator strength
1.3332
0.9999
0.6666
0.3333
ε (dm³ mol⁻¹ cm⁻¹)
60000
45000
30000
15000

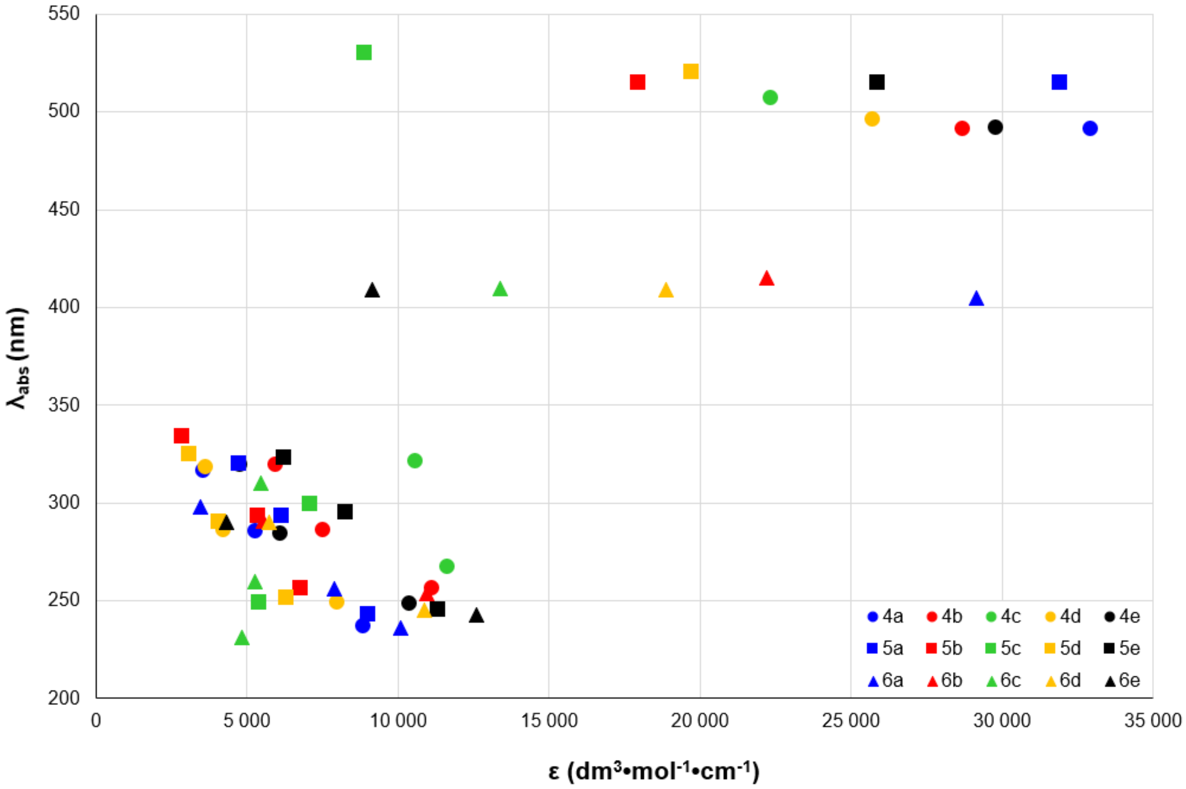

Figure S1. Wavelengths of absorption maxima in relation to their molar absorption coefficients for the studied compounds.

3. Plots of the most important molecular orbitals of compounds 4a-e, 5a-e, 6a-e

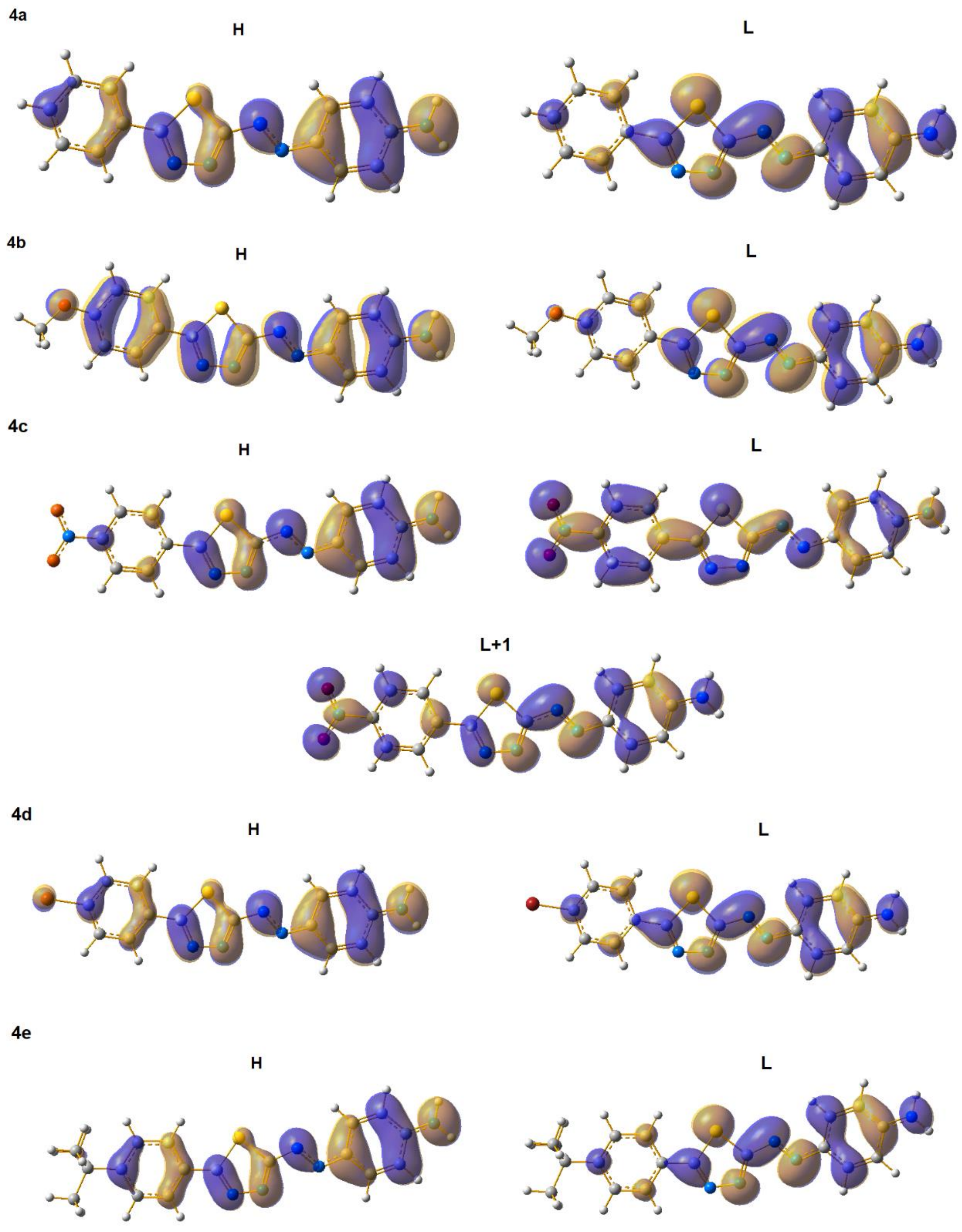

Figure S2. Calculated molecular orbitals for compounds belonging to series 4. The molecular orbitals are plotted with the isovalue equal to 0.02 au.

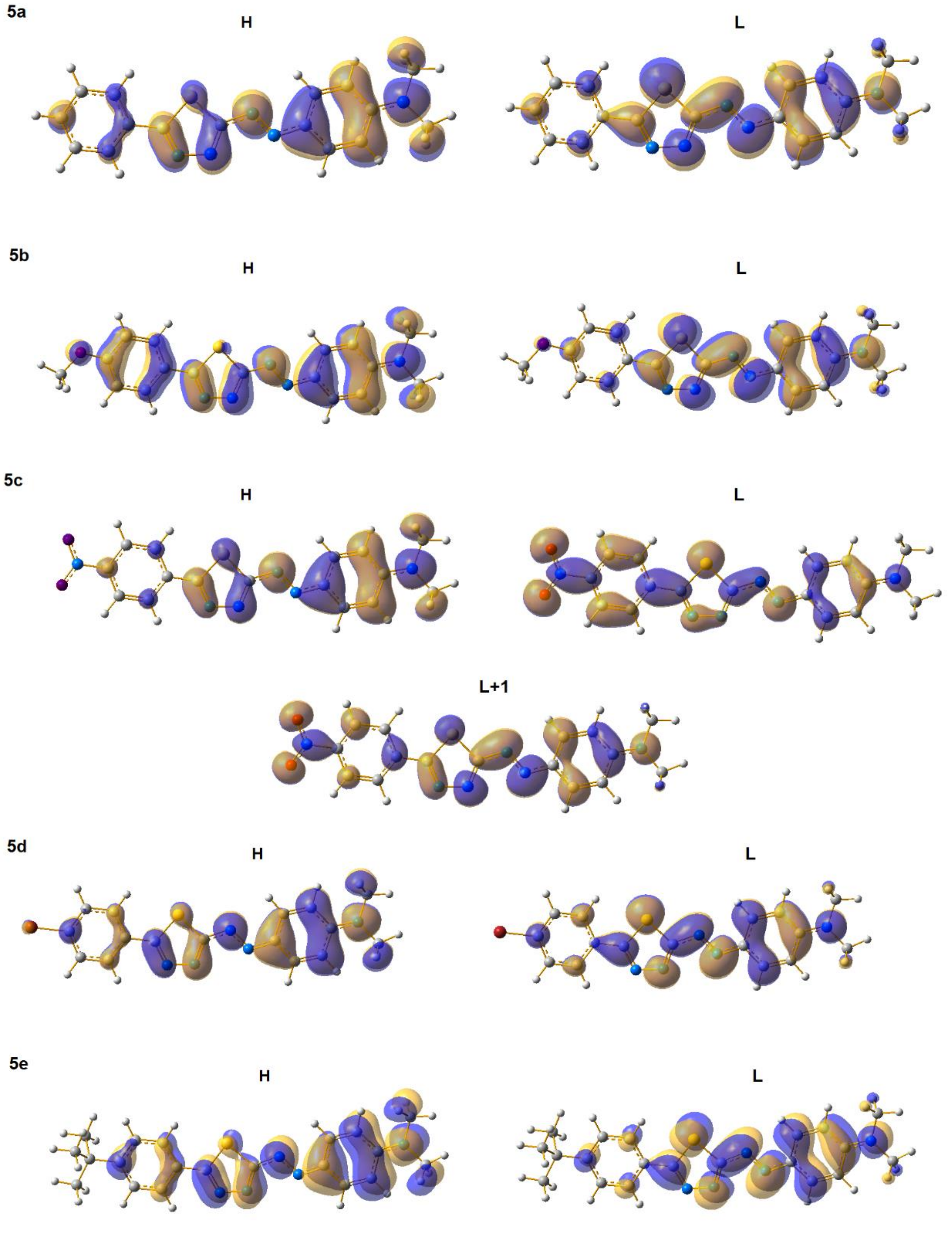

Figure S3. Calculated molecular orbitals for compounds belonging to series 5. The molecular orbitals are plotted with the isovalue equal to 0.02 au.

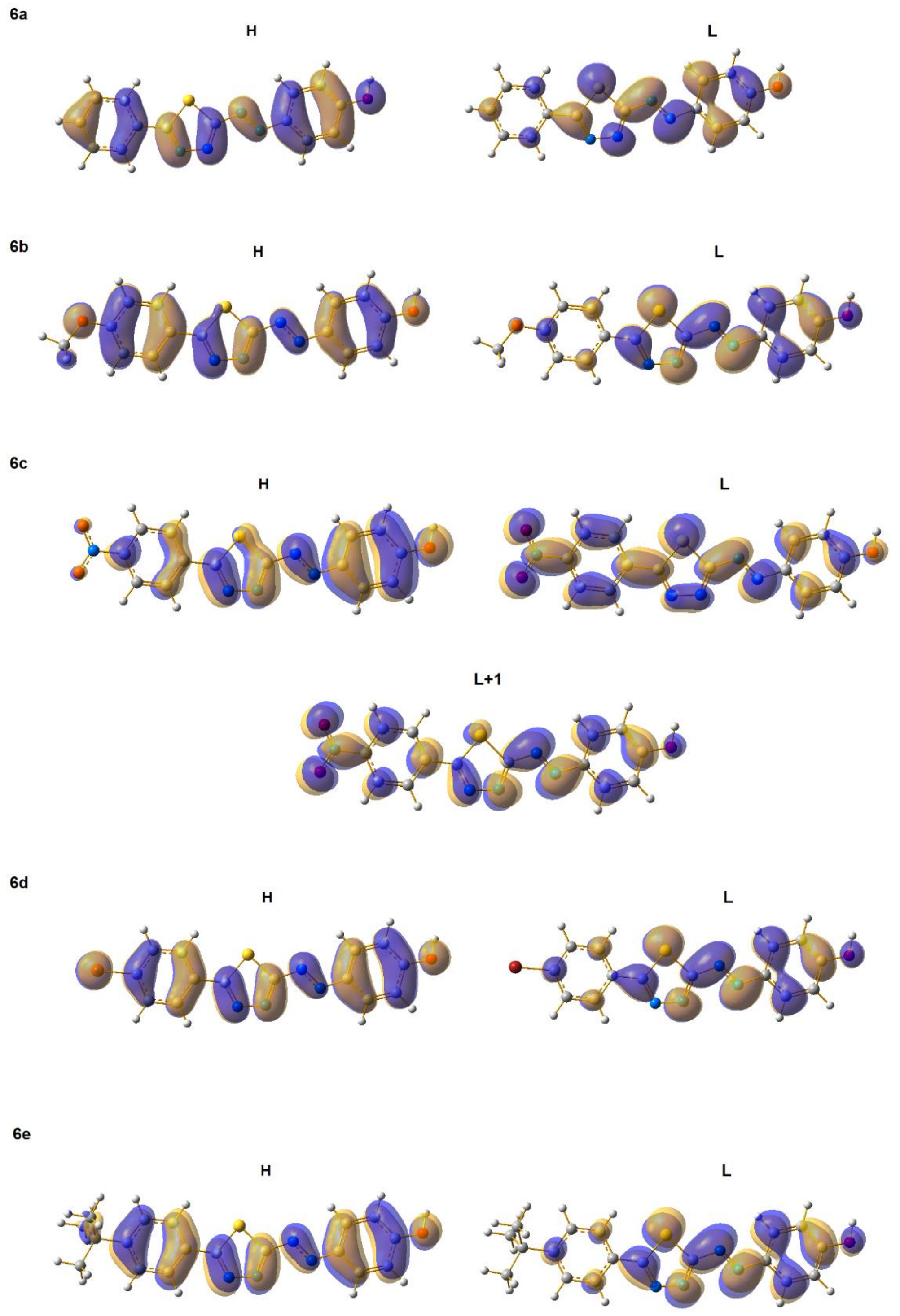

Figure S4. Calculated molecular orbitals for compounds belonging to series 6. The molecular orbitals are plotted with the isovalue equal to 0.02 au.

4. FT-IR spectra of compounds 4a-e, 5a-e, 6a-e

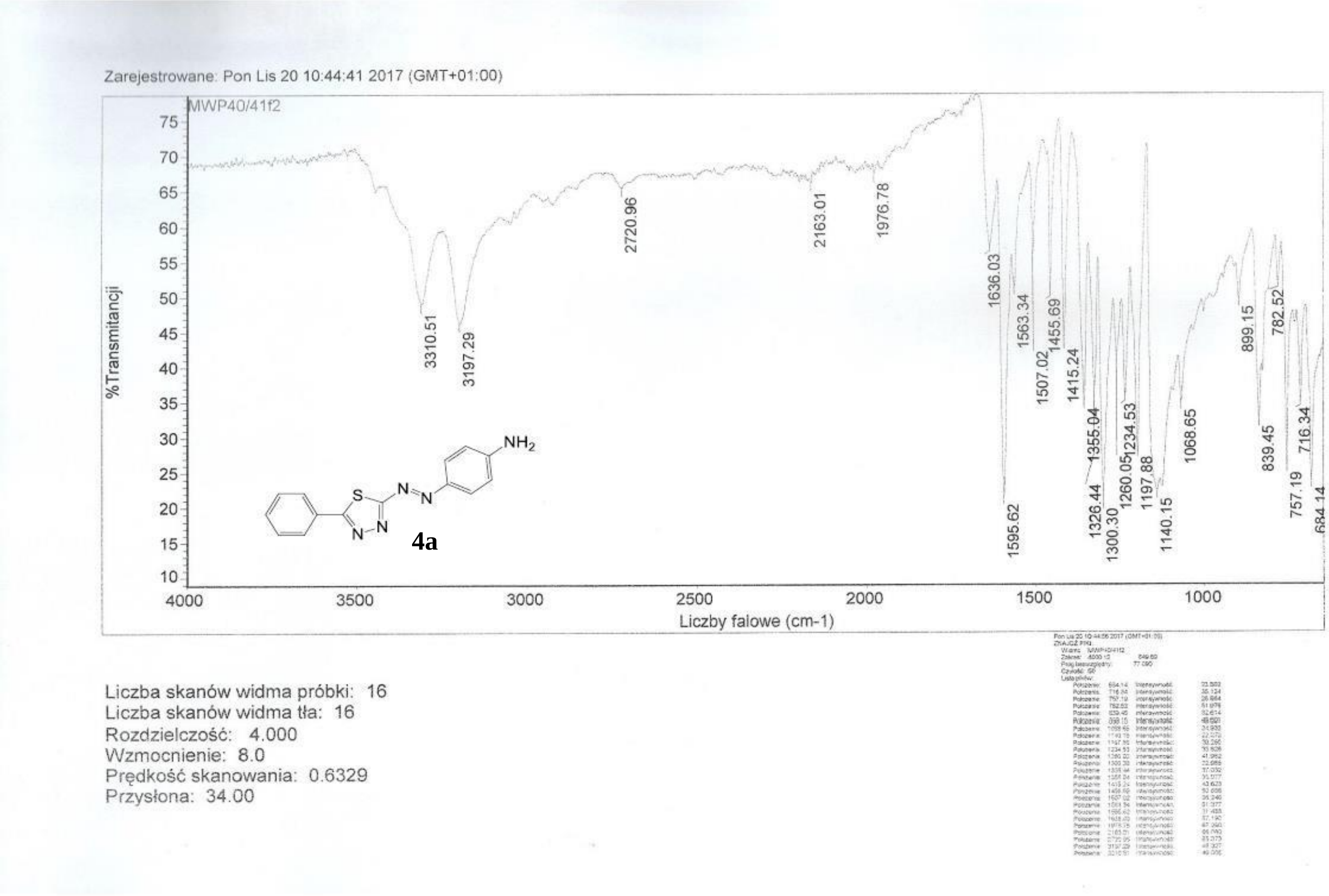

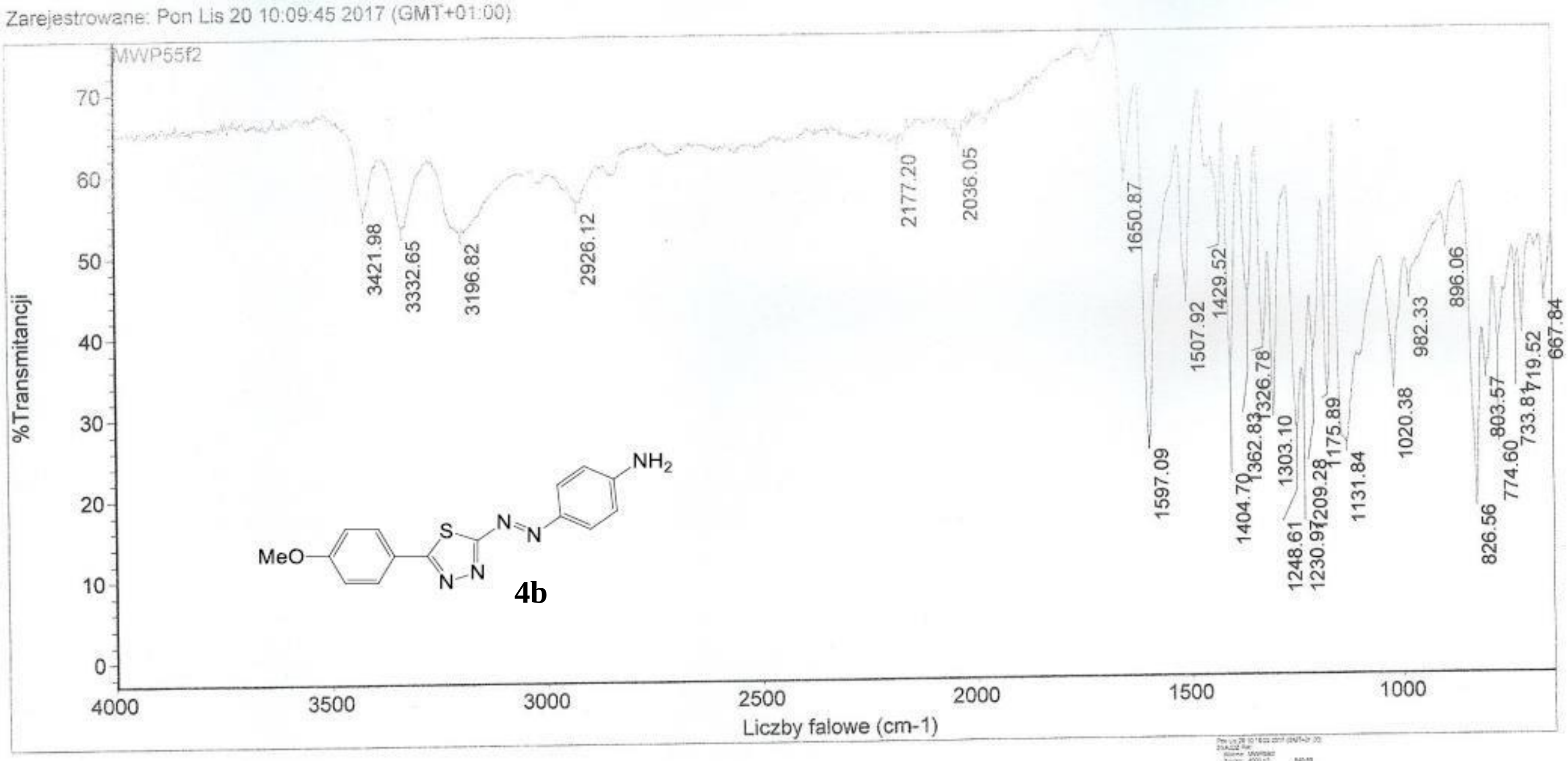

Liczba skanów widma próbki: 16
Liczba skanów widma tła: 16
Rozdzielczość: 4.000
Wzmocnienie: 8.0
Prędkość skanowania: 0.6329
Przysłona: 34.00

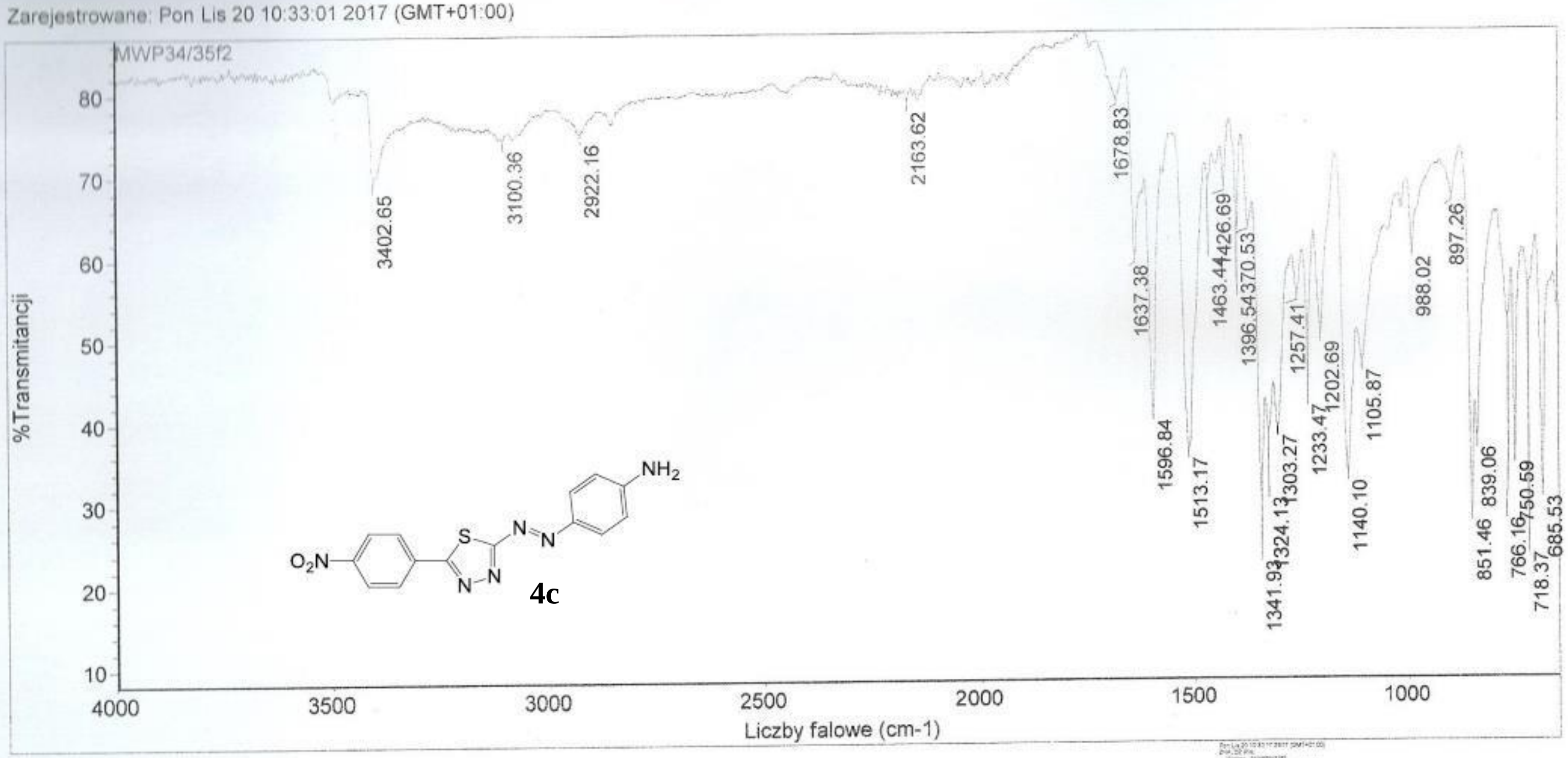

Liczba skanów widma próbki: 16
Liczba skanów widma tła: 16
Rozdzielczość: 4.000
Wzmocnienie: 8.0
Prędkość skanowania: 0.6329
Przysłona: 34.00

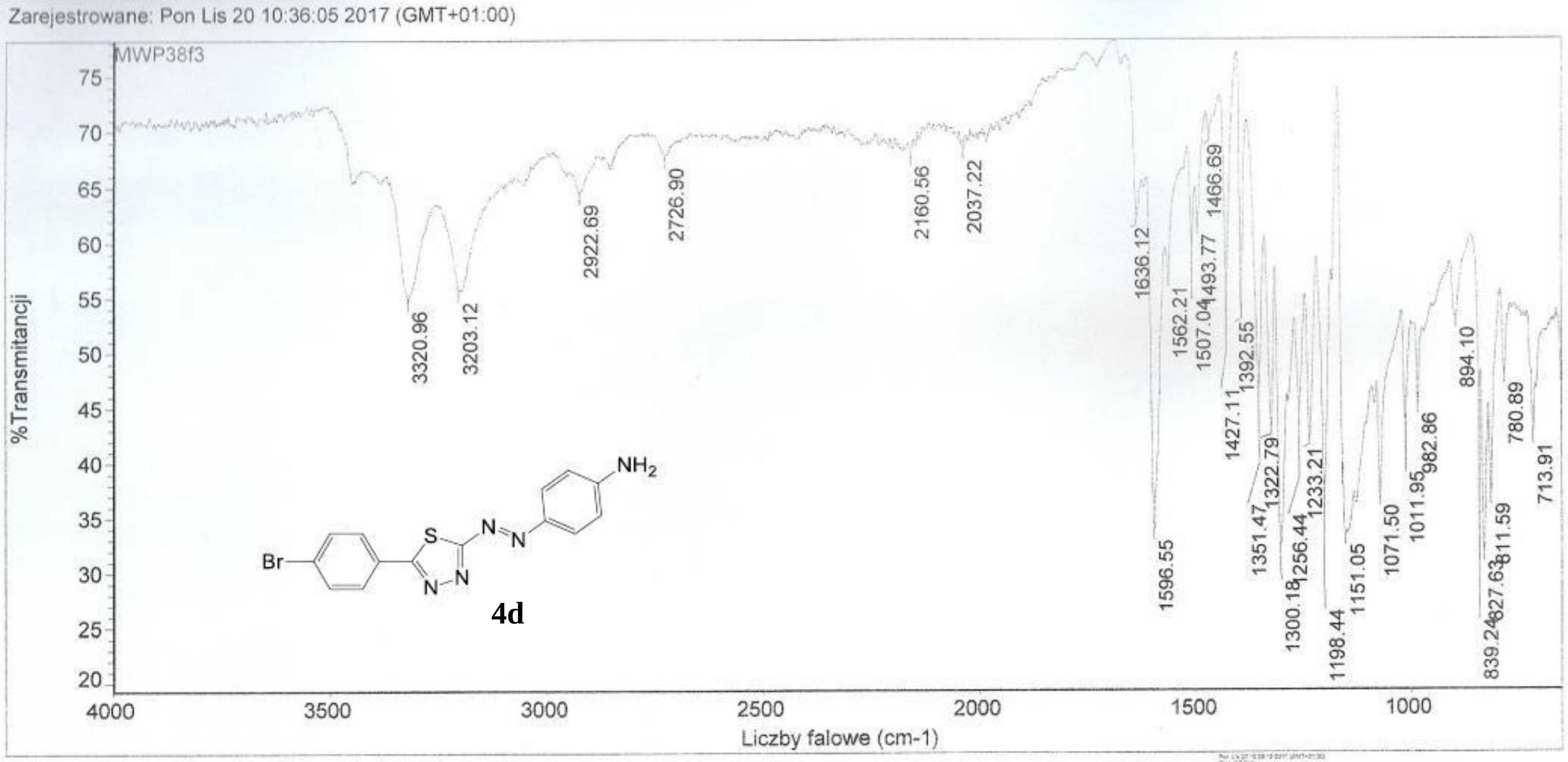

[illegible]

Liczba skanów widma próbki: 16
Liczba skanów widma tła: 16
Rozdzielczość: 4.000
Wzmocnienie: 8.0
Prędkość skanowania: 0.6329
Przysłona: 34.00

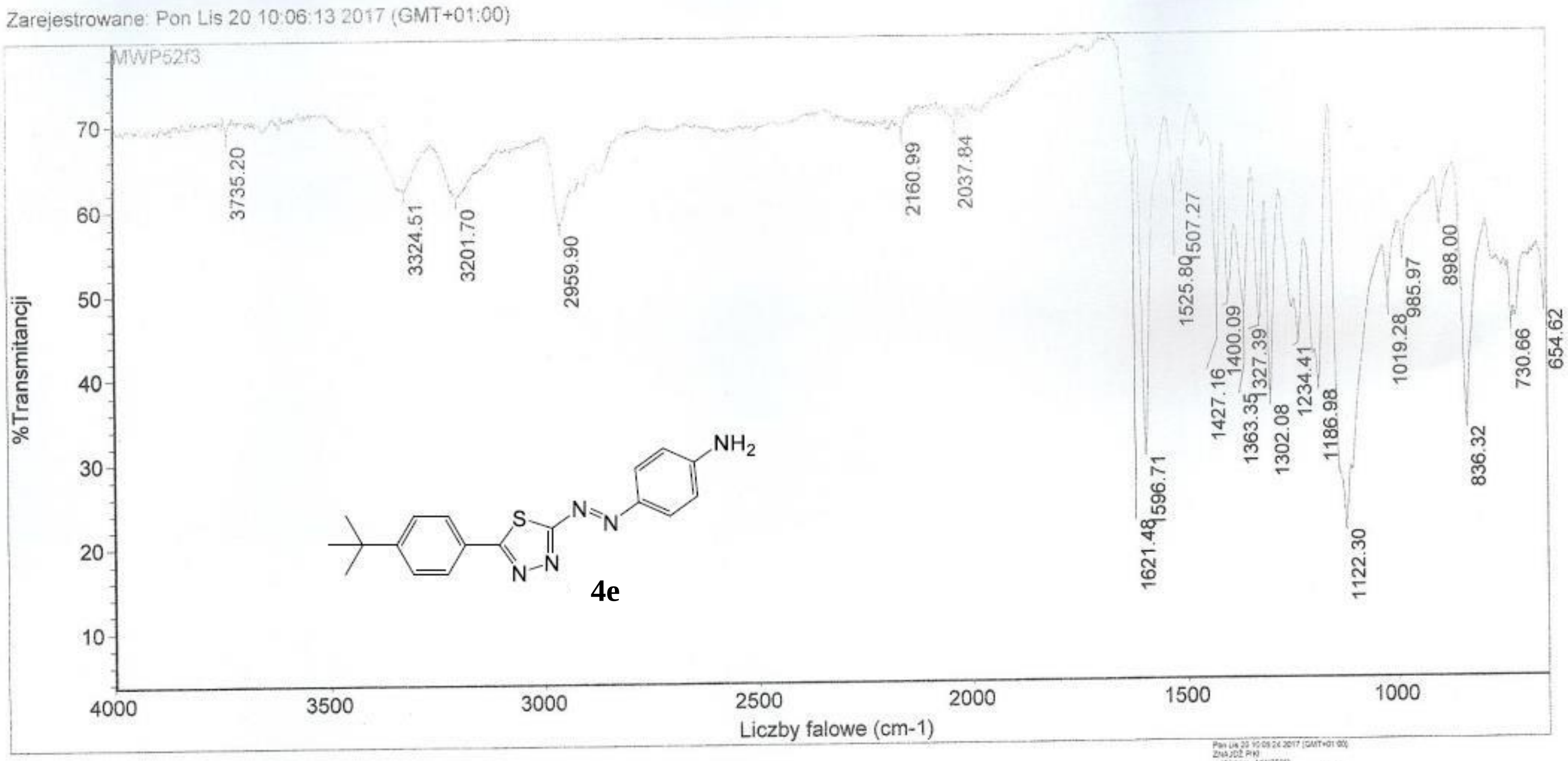

Liczba skanów widma próbki: 16
Liczba skanów widma tła: 16
Rozdzielczość: 4.000
Wzmocnienie: 8.0
Prędkość skanowania: 0.6329
Przysłona: 34.00

Zarejestrowane: Pon Lis 20 10:48:46 2017 (GMT+01:00)

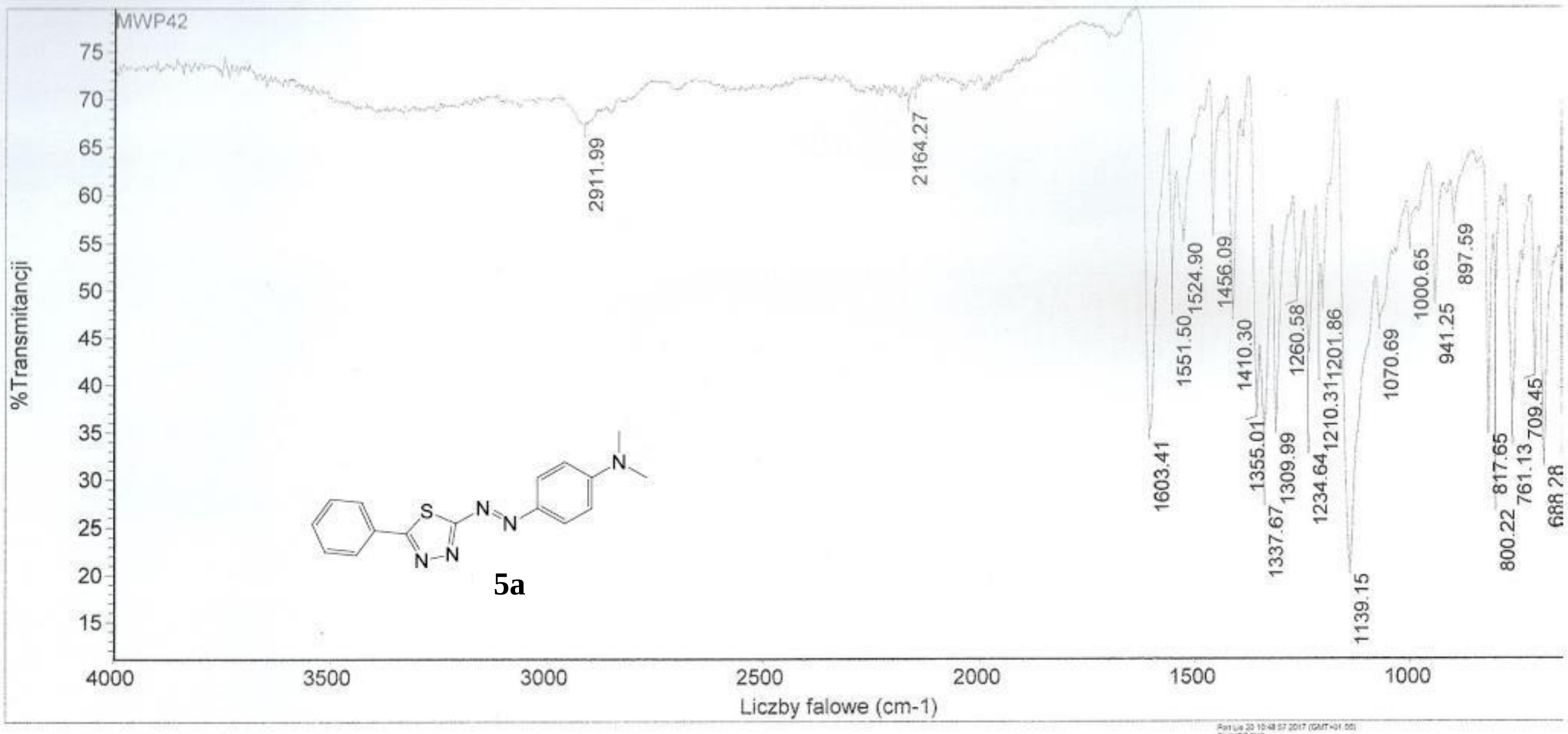

Liczba skanów widma próbki: 16
Liczba skanów widma tła: 16
Rozdzielczość: 4.000
Wzmocnienie: 8.0
Prędkość skanowania: 0.6329
Przysłona: 34.00

Pon Lis 20 10:48:57 2017 (GMT+01:00)
ZNAJDŹ PIKI:
Widmo: MWP42
Zakres: 4000.12 645.89
Próg bezwzględny: 78.930
Czułość: 50
Lista pików:

Położenie:	688.28	Intensywność:	30.912
Położenie:	709.45	Intensywność:	41.647
Położenie:	761.13	Intensywność:	38.173
Położenie:	800.22	Intensywność:	50.714
Położenie:	817.65	Intensywność:	35.937
Położenie:	897.59	Intensywność:	58.080
Położenie:	941.25	Intensywność:	49.727
Położenie:	1000.65	Intensywność:	56.538
Położenie:	1070.69	Intensywność:	47.044
Położenie:	1139.15	Intensywność:	21.160
Położenie:	1201.86	Intensywność:	49.157
Położenie:	1210.31	Intensywność:	[illegible]
Położenie:	1234.64	Intensywność:	43.161
Położenie:	1260.58	Intensywność:	40.998
Położenie:	1309.99	Intensywność:	33.980
Położenie:	1337.67	Intensywność:	30.675
Położenie:	1355.01	Intensywność:	37.262
Położenie:	1410.30	Intensywność:	40.070
Położenie:	1456.09	Intensywność:	[illegible]
Położenie:	1524.90	Intensywność:	[illegible]
Położenie:	1551.50	Intensywność:	52.794
Położenie:	1603.41	Intensywność:	[illegible]
Położenie:	2164.27	Intensywność:	69.580
Położenie:	2911.99	Intensywność:	67.274

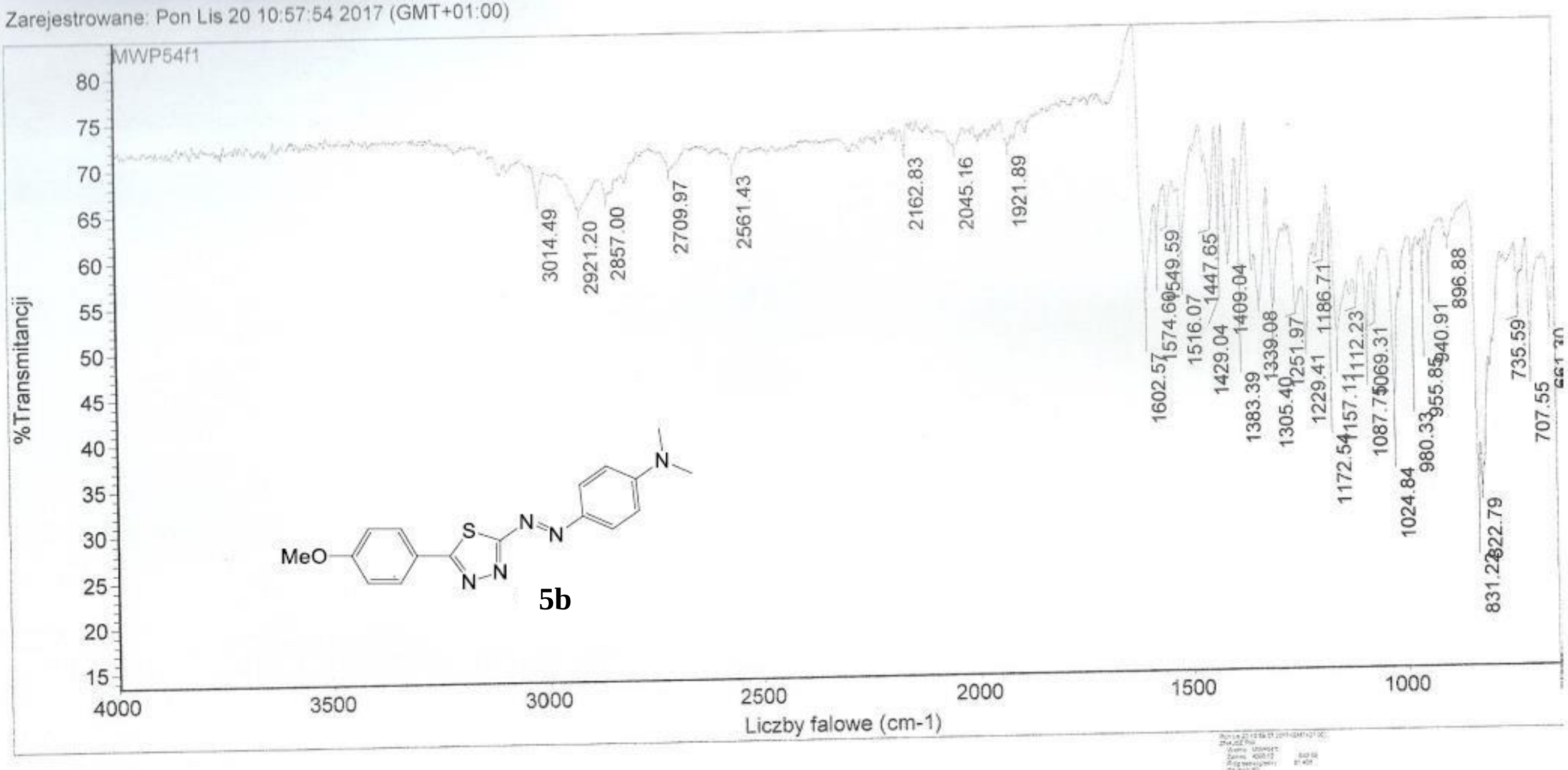

Liczba skanów widma próbki: 16
Liczba skanów widma tła: 16
Rozdzielczość: 4.000
Wzmocnienie: 8.0
Prędkość skanowania: 0.6329
Przysłona: 34.00

[illegible]

Zarejestrowane: Pon Lis 20 10:27:47 2017 (GMT+01:00)

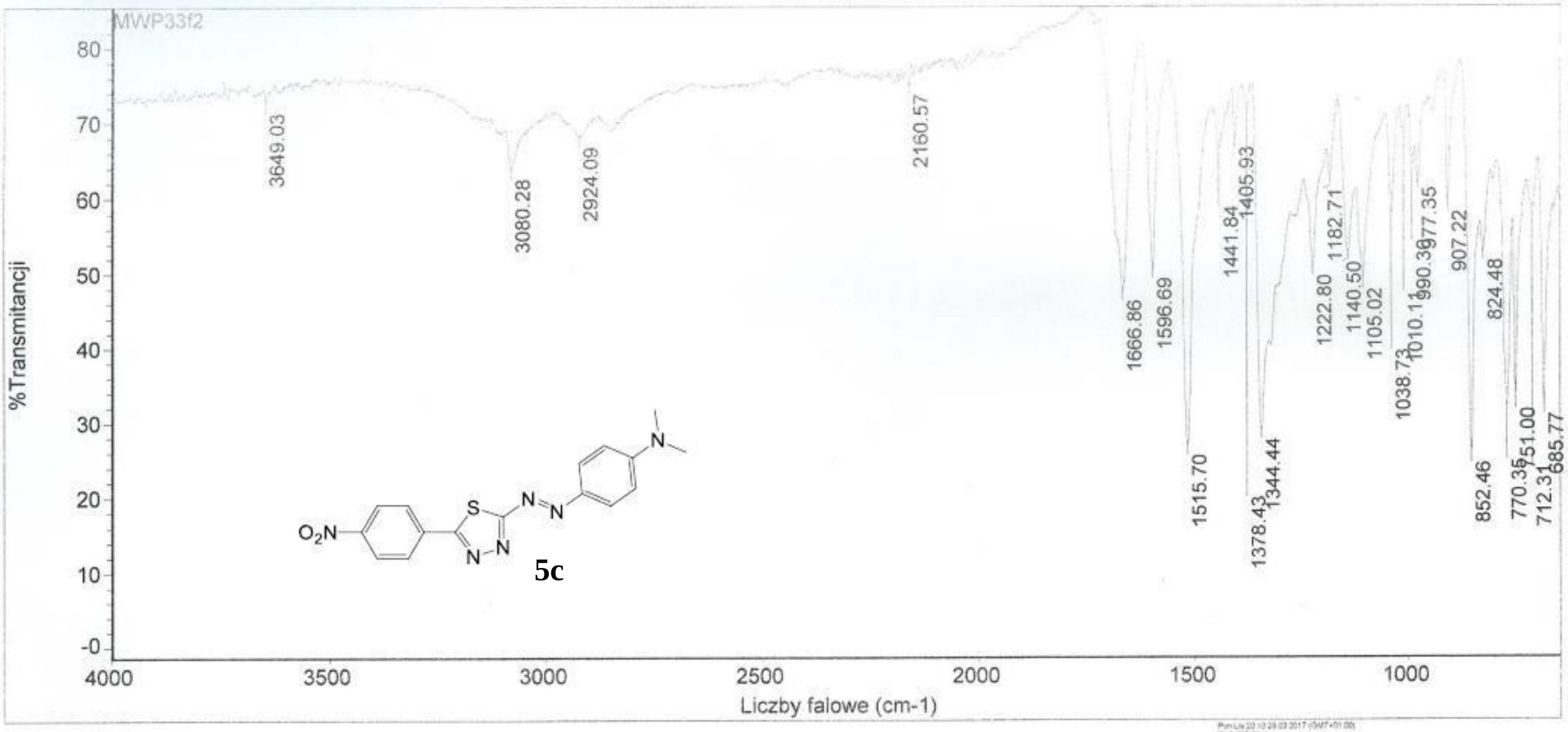

Liczba skanów widma próbki: 16
Liczba skanów widma tła: 16
Rozdzielczość: 4.000
Wzmocnienie: 8.0
Prędkość skanowania: 0.6329
Przysłona: 34.00

Pon Lis 20 10:28:03 2017 (GMT+01:00)
ZNAJDŹ PIKI:
Widmo: MWP33f2
Zakres: 4000.12 649.89
Próg bezwzględny: 85.211
Czułość: 50
Lista pików:

Położenie:	685.77	Intensywność:	30.247
Położenie:	712.31	Intensywność:	57.790
Położenie:	751.00	Intensywność:	32.997
Położenie:	770.35	Intensywność:	32.295
Położenie:	824.48	Intensywność:	53.043
Położenie:	852.46	Intensywność:	25.933
Położenie:	907.22	Intensywność:	59.491
Położenie:	977.35	Intensywność:	62.947
Położenie:	990.30	Intensywność:	64.325
Położenie:	1010.11	Intensywność:	58.125
Położenie:	1038.73	Intensywność:	37.897
Położenie:	1105.02	Intensywność:	49.111
Położenie:	1140.50	Intensywność:	52.704
Położenie:	1182.71	Intensywność:	62.360
Położenie:	1222.80	Intensywność:	30.924
Położenie:	1344.44	Intensywność:	29.054
Położenie:	1378.43	Intensywność:	71.779
Położenie:	1405.93	Intensywność:	67.974
Położenie:	1441.84	Intensywność:	66.828
Położenie:	1515.70	Intensywność:	26.784
Położenie:	1596.69	Intensywność:	53.864
Położenie:	1666.86	Intensywność:	47.025
Położenie:	2160.57	Intensywność:	75.055
Położenie:	2924.09	Intensywność:	69.150
Położenie:	3080.28	Intensywność:	63.640
Położenie:	3649.03	Intensywność:	72.669

Zarejestrowane: Pon Lis 20 10:40:18 2017 (GMT+01:00)

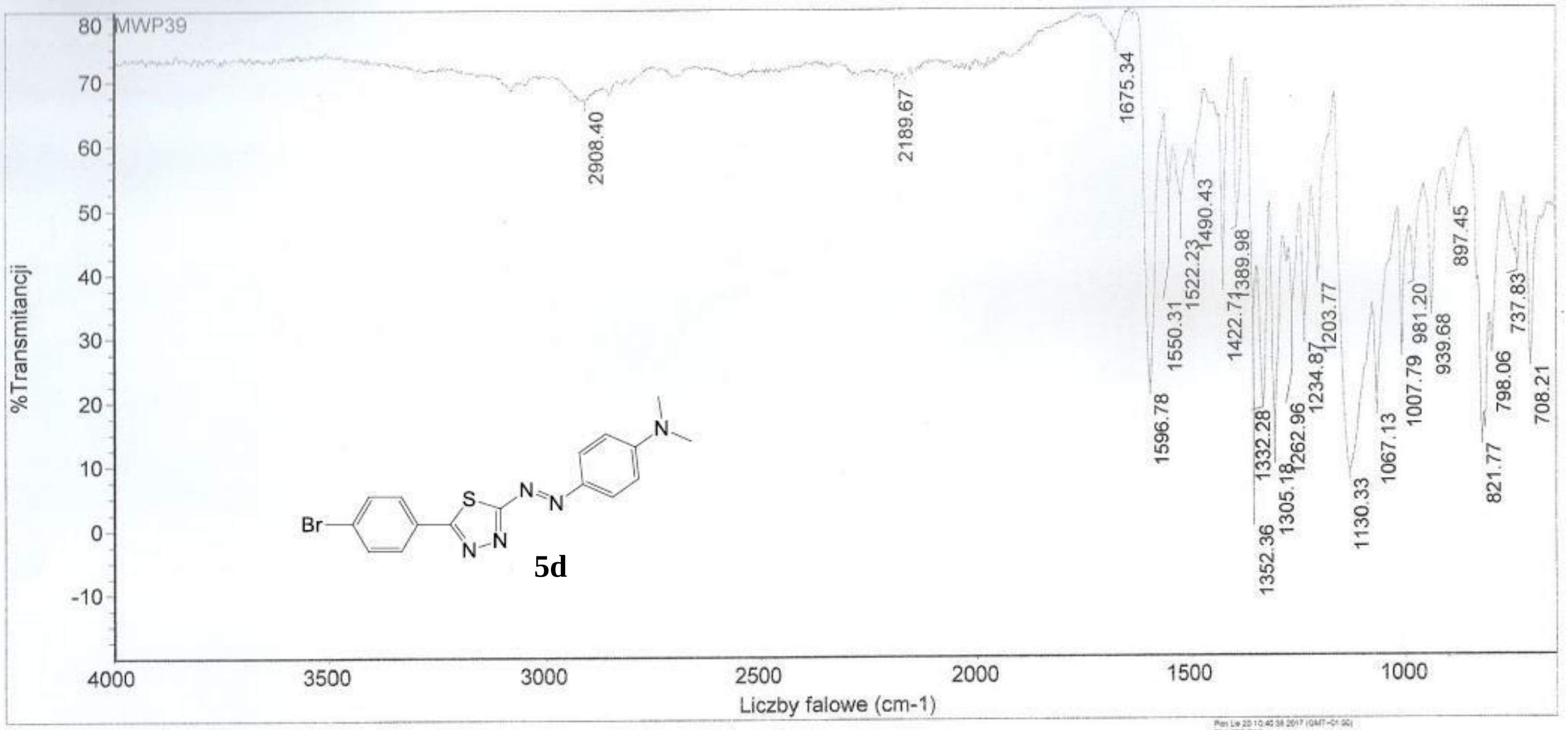

Pon Lis 20 10:40:38 2017 (GMT+01:00)
ZNAJDŹ PIKI
Widmo: MWP39
Zakres: 4000.12 649.89
Próg bezwzględny: 75.500
Czułość: 50
Lista pików:

Położenie:	708.21	Intensywność:	38.596
Położenie:	737.83	Intensywność:	40.843
Położenie:	798.06	Intensywność:	28.621
Położenie:	821.77	Intensywność:	14.277
Położenie:	897.45	Intensywność:	51.390
Położenie:	939.68	Intensywność:	34.574
Położenie:	981.20	Intensywność:	39.373
Położenie:	1007.79	Intensywność:	27.997
Położenie:	1067.13	Intensywność:	19.049
Położenie:	1130.33	Intensywność:	8.867
Położenie:	1203.77	Intensywność:	39.578
Położenie:	1234.87	Intensywność:	32.735
Położenie:	1262.96	Intensywność:	25.265
Położenie:	1305.18	Intensywność:	17.437
Położenie:	1332.28	Intensywność:	19.673
Położenie:	1352.36	Intensywność:	[illegible]
Położenie:	1389.98	Intensywność:	48.011
Położenie:	1422.71	Intensywność:	43.225
Położenie:	1490.43	Intensywność:	55.706
Położenie:	1522.23	Intensywność:	51.563
Położenie:	1550.31	Intensywność:	53.068
Położenie:	1596.78	Intensywność:	22.294
Położenie:	1675.34	Intensywność:	75.504
Położenie:	2189.67	Intensywność:	69.967
Położenie:	2908.40	Intensywność:	66.793

Liczba skanów widma próbki: 16
Liczba skanów widma tła: 16
Rozdzielczość: 4.000
Wzmocnienie: 8.0
Prędkość skanowania: 0.6329
Przysłona: 34.00

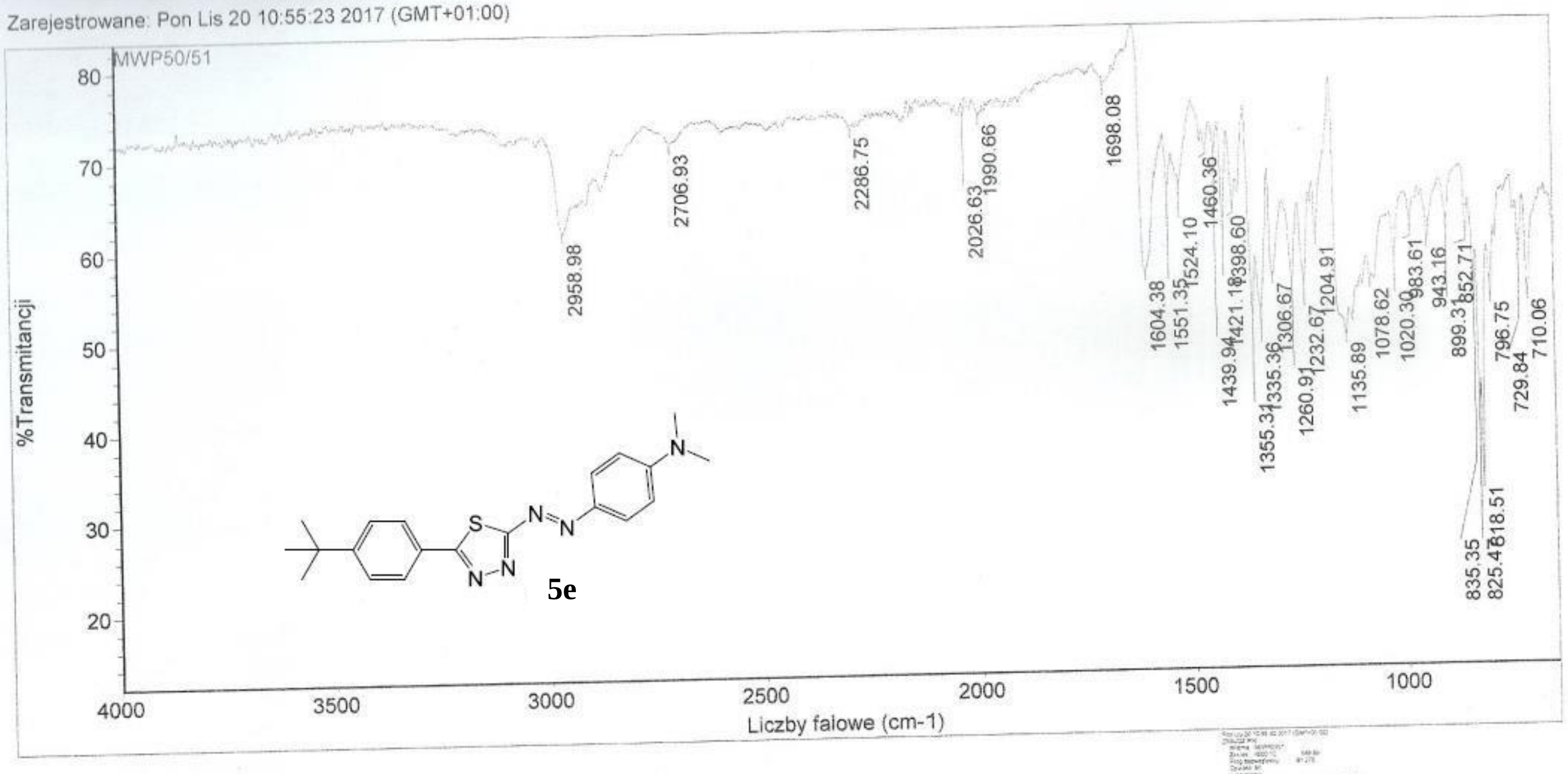

Liczba skanów widma próbki: 16
Liczba skanów widma tła: 16
Rozdzielczość: 4.000
Wzmocnienie: 8.0
Prędkość skanowania: 0.6329
Przysłona: 34.00

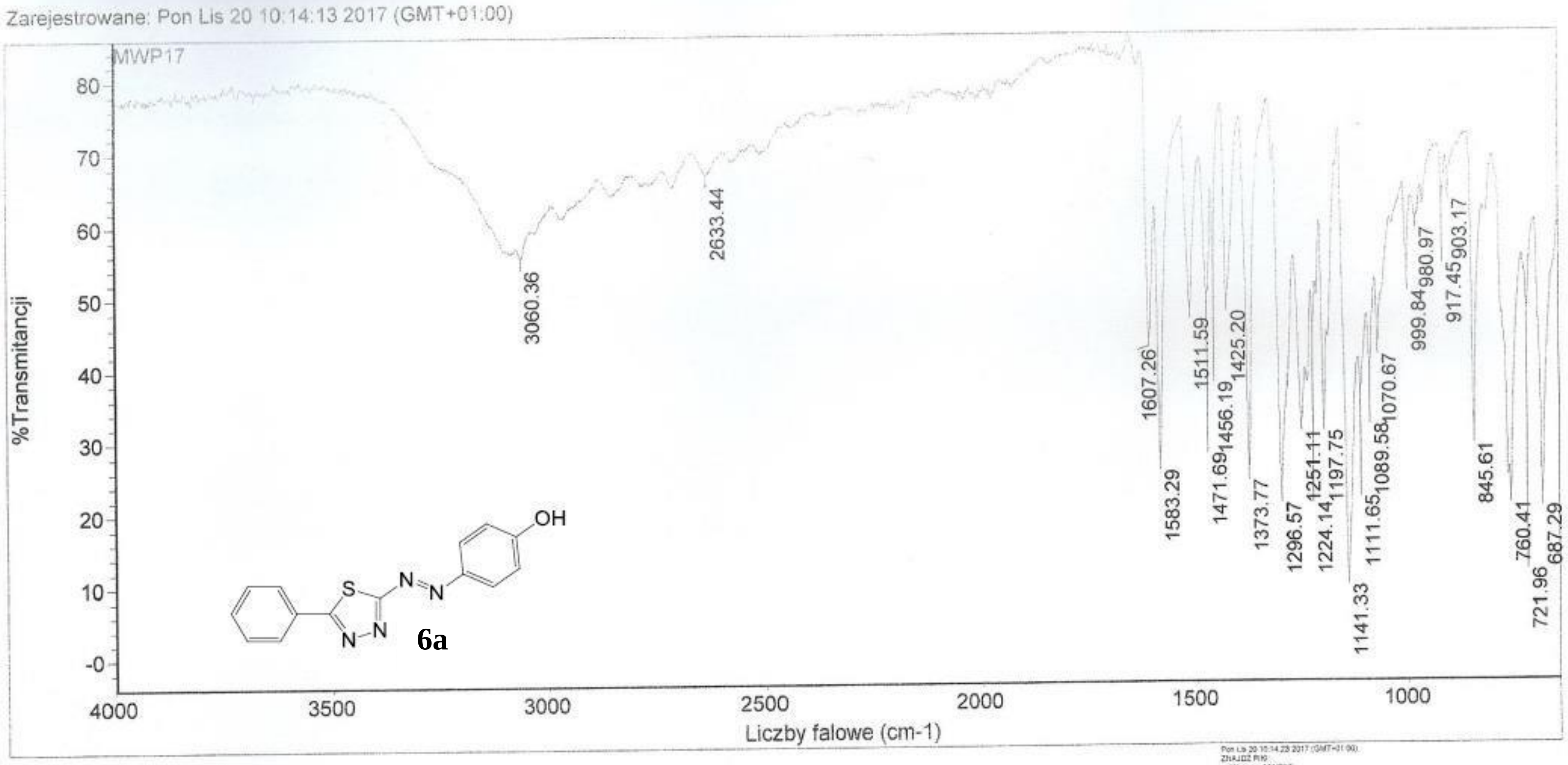

Liczba skanów widma próbki: 16
Liczba skanów widma tła: 16
Rozdzielczość: 4.000
Wzmocnienie: 8.0
Prędkość skanowania: 0.6329
Przysłona: 34.00

Pon Lis 20 10:14:23 2017 (GMT+01:00)
ZNAJDŹ PIKI:
Widmo: MWP17
Zakres: 4000.12 649.69
Próg bezwzględny: 63.524
Czułość: 50
Lista pików:

Położenie:	687.29	Intensywność:	21.551
Położenie:	721.96	Intensywność:	47.687
Położenie:	760.41	Intensywność:	22.141
Położenie:	845.61	Intensywność:	30.275
Położenie:	903.17	Intensywność:	64.212
Położenie:	917.45	Intensywność:	63.468
Położenie:	980.97	Intensywność:	60.166
Położenie:	999.84	Intensywność:	54.825
Położenie:	1070.67	Intensywność:	42.797
Położenie:	1089.58	Intensywność:	39.707
Położenie:	1111.65	Intensywność:	35.605
Położenie:	1141.33	Intensywność:	10.733
Położenie:	1197.75	Intensywność:	32.141
Położenie:	1224.14	Intensywność:	44.950
Położenie:	1251.11	Intensywność:	32.110
Położenie:	1296.57	Intensywność:	23.439
Położenie:	1373.77	Intensywność:	25.373
Położenie:	1425.20	Intensywność:	48.908
Położenie:	1456.19	Intensywność:	53.581
Położenie:	1471.69	Intensywność:	49.771
Położenie:	1511.59	Intensywność:	47.623
Położenie:	1583.29	Intensywność:	28.890
Położenie:	1607.26	Intensywność:	43.603
Położenie:	2633.44	Intensywność:	66.738
Położenie:	3060.36	Intensywność:	55.479

Zarejestrowane: Pon Lis 20 11:00:27 2017 (GMT+01:00)

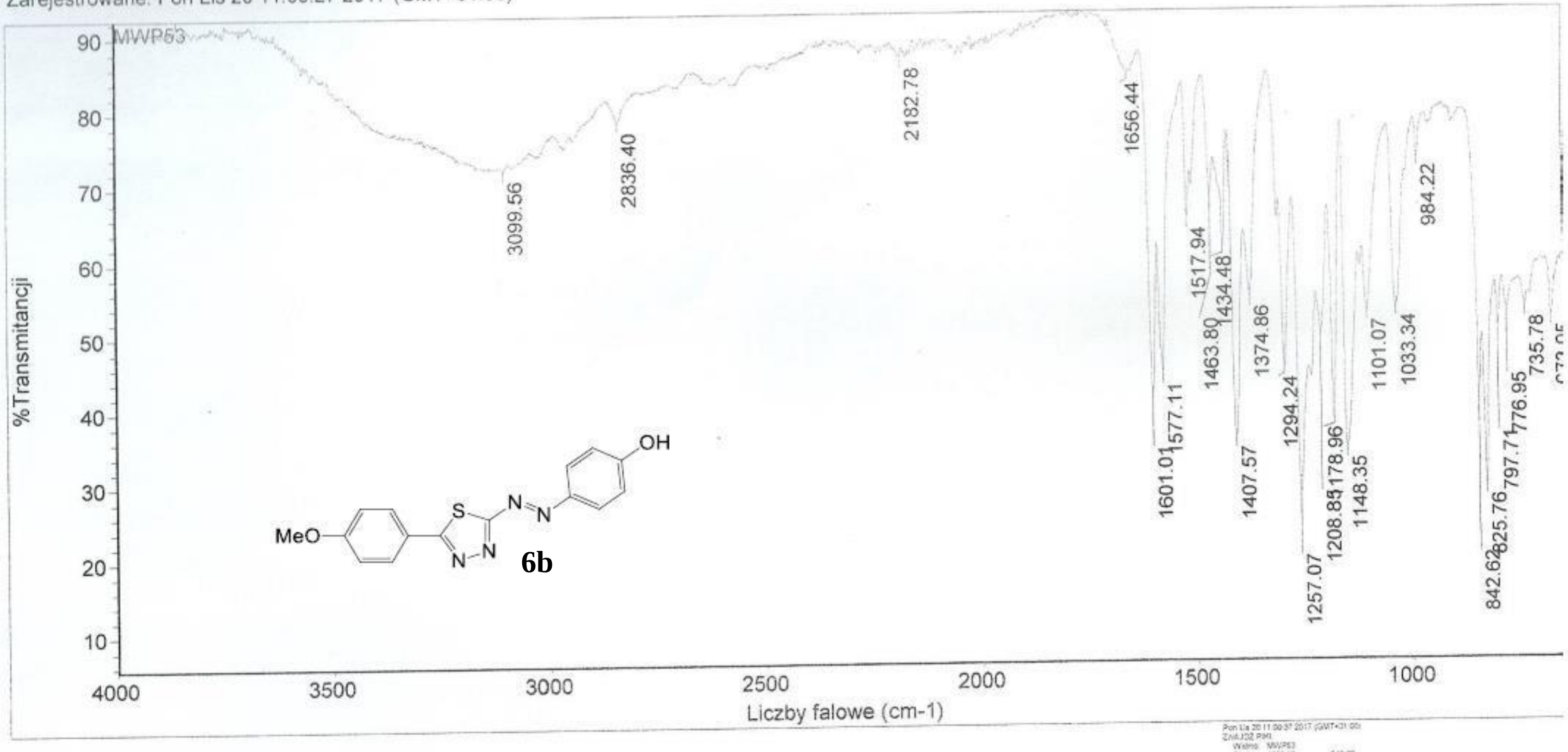

Liczba skanów widma próbki: 16
Liczba skanów widma tła: 16
Rozdzielczość: 4.000
Wzmocnienie: 8.0
Prędkość skanowania: 0.6329
Przysłona: 34.00

Pon Lis 20 11:00:37 2017 (GMT+01:00)
ZNAJDŹ PIKI
Widmo: MWP53
Zakres: 4000.12 549.09
Próg bezwzględny [illegible]
Czułość: 50
Lista pików:

Położenie:	673.95	Intensywność:	61.439
Położenie:	735.78	Intensywność:	62.562
Położenie:	776.95	Intensywność:	62.446
Położenie:	797.71	Intensywność:	[illegible]
Położenie:	825.76	Intensywność:	28.740
Położenie:	842.62	Intensywność:	[illegible]
Położenie:	984.22	Intensywność:	72.660
Położenie:	1033.34	Intensywność:	[illegible]
Położenie:	1101.07	Intensywność:	62.284
Położenie:	1148.35	Intensywność:	[illegible]
Położenie:	1178.96	Intensywność:	37.690
Położenie:	1208.85	Intensywność:	49.402
Położenie:	1257.07	Intensywność:	[illegible]
Położenie:	1294.24	Intensywność:	[illegible]
Położenie:	1374.86	Intensywność:	[illegible]
Położenie:	1407.57	Intensywność:	[illegible]
Położenie:	1434.48	Intensywność:	[illegible]
Położenie:	1463.80	Intensywność:	[illegible]
Położenie:	1517.94	Intensywność:	64.675
Położenie:	1577.11	Intensywność:	[illegible]
Położenie:	1601.01	Intensywność:	[illegible]
Położenie:	1656.44	Intensywność:	[illegible]
Położenie:	2182.78	Intensywność:	[illegible]
Położenie:	2836.40	Intensywność:	[illegible]
Położenie:	3099.56	Intensywność:	[illegible]

Zarejestrowane: Pon Lis 20 10:18:18 2017 (GMT+01:00)

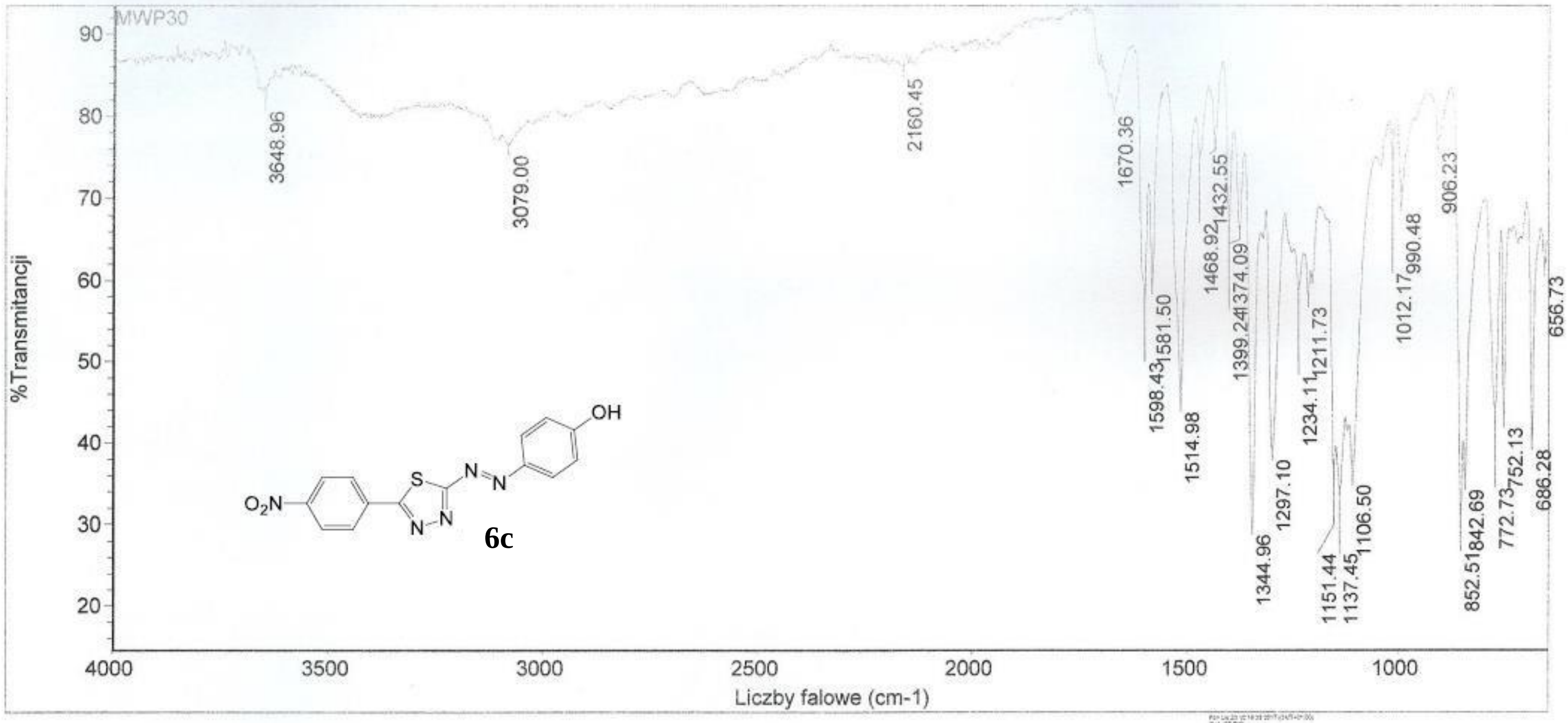

Liczba skanów widma próbki: 16
Liczba skanów widma tła: 16
Rozdzielczość: 4.000
Wzmocnienie: 8.0
Prędkość skanowania: 0.6329
Przysłona: 34.00

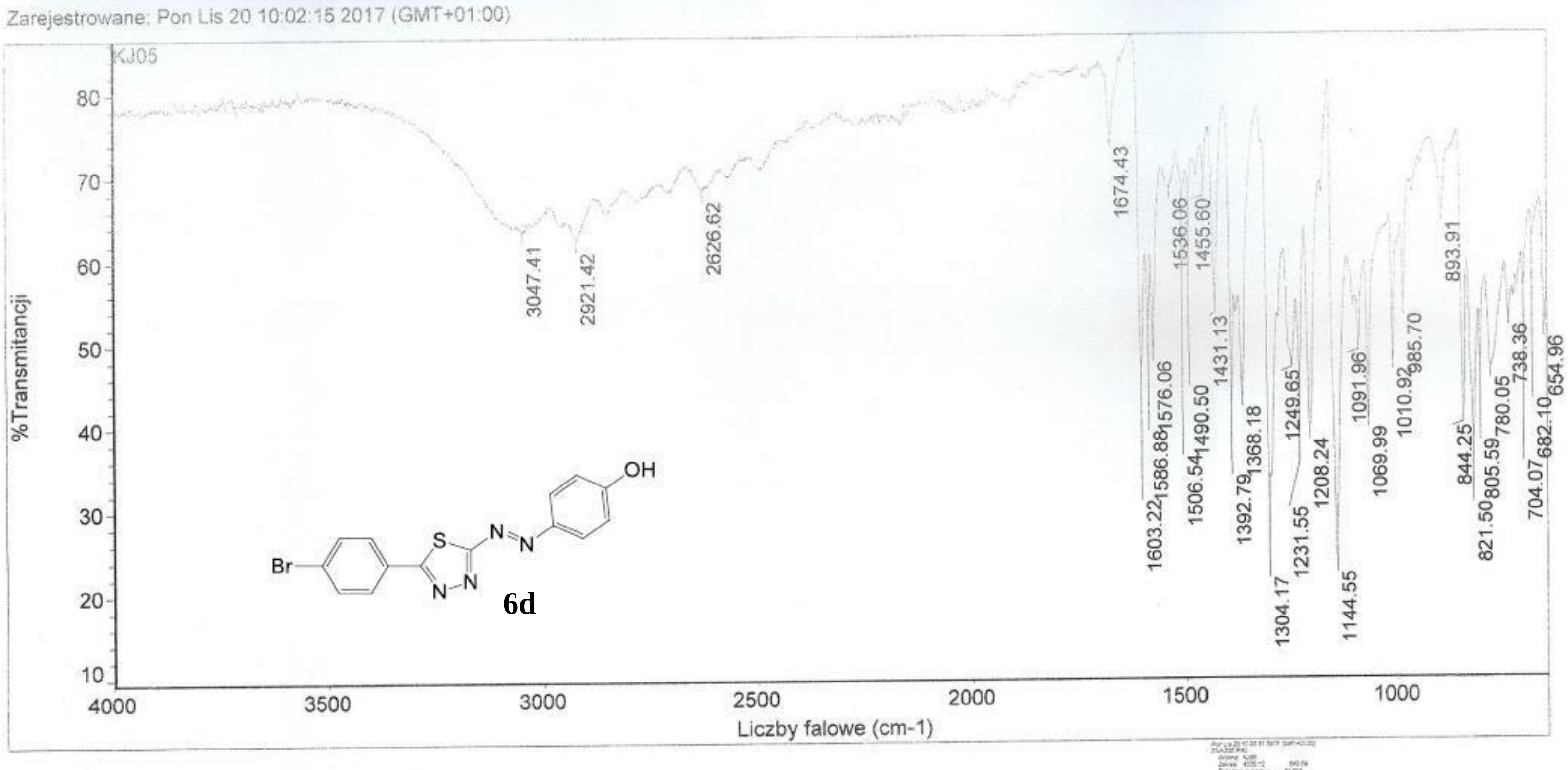

Liczba skanów widma próbki: 16
Liczba skanów widma tła: 16
Rozdzielczość: 4.000
Wzmocnienie: 8.0
Prędkość skanowania: 0.6329
Przysłona: 34.00

Zarejestrowane: Pon Lis 20 10:51:59 2017 (GMT+01:00)

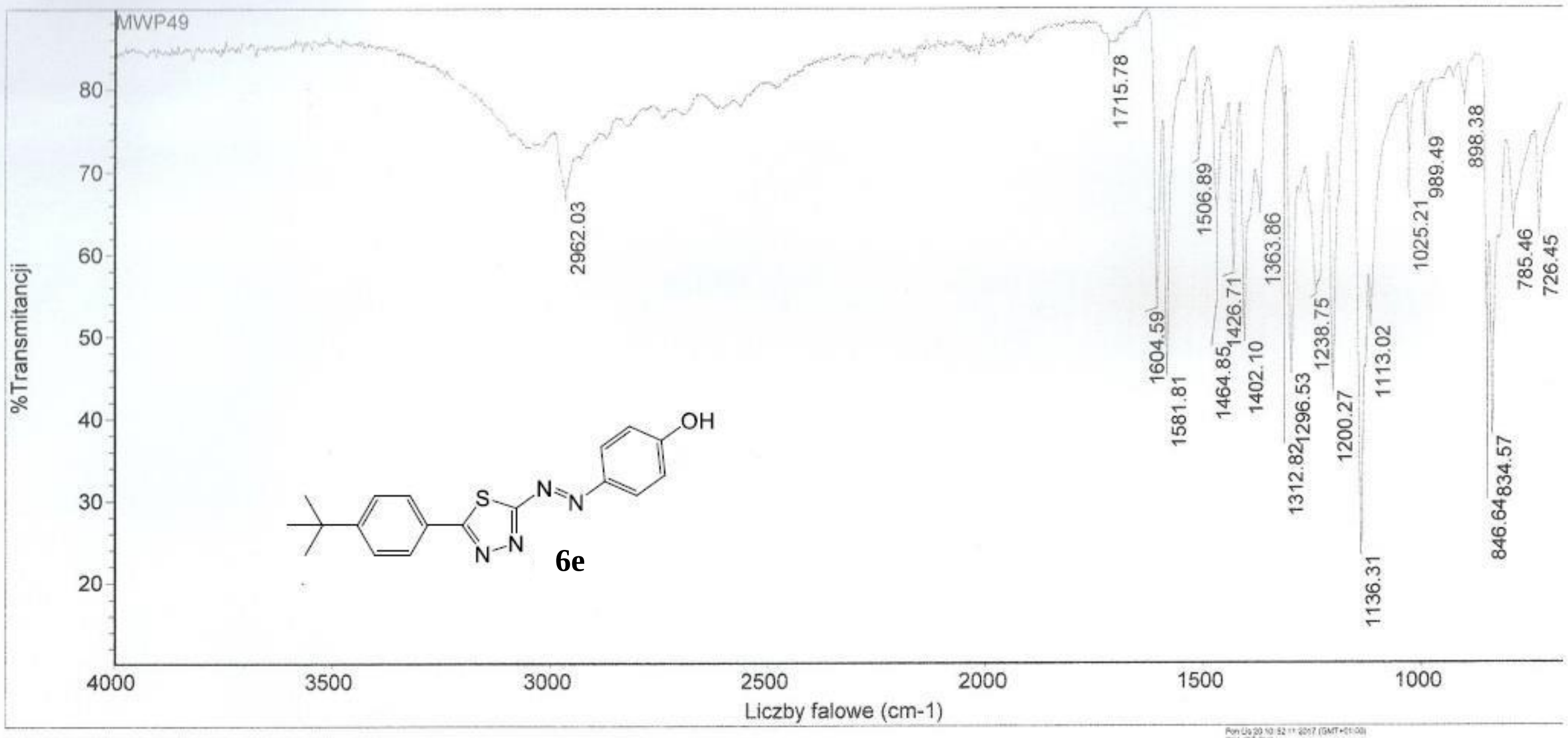

Pon Lis 20 10:52:11 2017 (GMT+01:00)
ZNAJDŹ PIKI:
Widmo: MWP49
Zakres: 4000.12 649.89
Próg bezwzględny: 67.369
Czułość: 50
Lista pików:

Położenie:	857.96	Intensywność:	71.100
Położenie:	726.45	Intensywność:	63.488
Położenie:	785.46	Intensywność:	64.194
Położenie:	834.57	Intensywność:	39.113
Położenie:	846.64	Intensywność:	32.838
Położenie:	898.38	Intensywność:	79.202
Położenie:	989.49	Intensywność:	75.536
Położenie:	1025.21	Intensywność:	72.274
Położenie:	1113.02	Intensywność:	52.432
Położenie:	1136.31	Intensywność:	24.440
Położenie:	1200.27	Intensywność:	44.591
Położenie:	1238.75	Intensywność:	59.635
Położenie:	1296.53	Intensywność:	46.504
Położenie:	1312.82	Intensywność:	77.745
Położenie:	1363.86	Intensywność:	68.288
Położenie:	1402.10	Intensywność:	60.604
Położenie:	1426.71	Intensywność:	55.492
Położenie:	1464.85	Intensywność:	22.796
Położenie:	1506.89	Intensywność:	77.348
Położenie:	1581.81	Intensywność:	48.563
Położenie:	1604.59	Intensywność:	54.283
Położenie:	1715.78	Intensywność:	55.402
Położenie:	2962.03	Intensywność:	67.870

Liczba skanów widma próbki: 16
Liczba skanów widma tła: 16
Rozdzielczość: 4.000
Wzmocnienie: 8.0
Prędkość skanowania: 0.6329
Przysłona: 34.00

5. Crystal structure determination

A red plate crystal of 2-[4-(*N*,*N*-dimethylamino)phenylazo]-5-(4-methoxyphenyl)-1,3,4-thiadiazole (**5b**) was mounted on a Rigaku Synergy Dualflex automatic diffractometer equipped with Pilatus 300K detector, and used for data collection. X-Ray intensity data was collected with mirror monochromatic CuK_{α} (λ = 1.54178 Å, micro-focus sealed PhotonJet X-ray tube) radiation at a temperature of 100.0(1) K, with ω scan mode. The shutterless mode was used, and reflections inside the Ewald sphere were collected up to θ = 78.9°. The unit cell parameters were determined from the 13352 strongest reflections. Details concerning crystal data and refinement are given in **Table 1**. Examination of the same reference reflections, obtained before and after measurement, showed no loss in intensity during measurement. Lorentz polarization, and Gaussian absorption (using Gaussian integration over a multifaceted crystal model) corrections were applied during data reduction. The structure was solved by a partial structure expansion procedure. All non-hydrogen atoms were refined anisotropically using a full-matrix, least-squares technique on F2. All hydrogen atoms were identified from different Fourier synthesis after four cycles of anisotropic refinement, and refined as "riding" on the adjacent atom with geometric idealisation after each refinement cycle. Individual isotropic displacement factors equalled 1.2 times the value of equivalent displacement factors of the aromatic carbon atoms, and 1.5 times of parent methyl carbon atoms. SHELXT [1], SHELXL [2], and SHELXTL [3] programs were used for all calculations. Atomic scattering factors were taken from International Tables for Crystallography [4]. Selected interatomic bond distances, dihedral angles and intermolecular interactions are presented in the publication. Tables of crystal data for **5b**, structure refinement, anisotropic displacement coefficients, atomic coordinates, and equivalent isotropic displacement parameters for non-hydrogen atoms, H-atom coordinates, and isotropic displacement parameters, bond lengths, and interbond angles were deposited with the Cambridge Crystallographic Data Centre under No. CCDC1946289.

Table 1. Crystal data and structure refinement details for compound **5b**.

Compound	5b
Empirical formula	$C_{17}H_{17}N_5OS$
Formula weight	339.41
Crystal system	Monoclinic
Space group	$P2_1/c$ (No. 14)
Temperature [K]	100.0(1)
Wavelength [Å]	λ(Cu$K\alpha$) 1.54184
Unit cell dimensions [Å, °]	a = 19.0674(2)
	b = 9.7671(1)
	c = 8.7924(1)
	α = 90.00
	β = 102.721(1)
	γ = 90.00
Volume [Å^3]	1597.24(3)
Z	4
Calculated density [Mg/m^3]	1.411
Absorption coefficient [mm^{-1}]	1.923
F(000)	712
Crystal size [mm]	0.013 x 0.060 x 0.129
θ Range for data collection [°]	4.755 to 78.905
Index ranges	$-24 \leq h \leq 24$,
	$-12 \leq k \leq 12$,
	$-10 \leq l \leq 10$
Reflections collected / unique (R_{int})	25674 / 3335 (0.0388)
Completeness to θ = 67° [%]	100.0
Min. and max. transmission	0.765 and 1.000

Data / restraints / parameters	3335 / 0 / 220
Goodness-of-fit on F^2	1.067
Final R indices [$I>2\sigma(I)$]	$R1$ = 0.0319,
	$wR2$ = 0.0820
R indices (all data)	$R1$ = 0.0360,
	$wR2$ = 0.0842
Largest diff. peak and hole [e **x** $Å^{-3}$]	0.283 and -0.310

References

1. Sheldrick, G.M. SHELXT - Integrated space-group and crystal-structure determination. *Acta Cryst.* **2015**, A71, 3-8.
2. Sheldrick, G.M. Crystal structure refinement with SHELXL. *Acta Cryst.* **2015**, C71, 3-8.
3. Sheldrick, G.M. A short history of SHELX. *Acta Cryst.* **2008**, A64, 112-122.
4. Cowley, J.M. Scattering factors for the diffraction of electrons by crystalline solids. *In International Tables for Crystallography, Volume C: Mathematical, physical and chemical tables*, 3rd ed.; Prince, E., Ed.; Kluwer Academic Publishers: Dordrecht, Netherlands, 2004; pp. 259-262.

checkCIF/PLATON report

Structure factors have been supplied for datablock(s) mwp54_f1

THIS REPORT IS FOR GUIDANCE ONLY. IF USED AS PART OF A REVIEW PROCEDURE FOR PUBLICATION, IT SHOULD NOT REPLACE THE EXPERTISE OF AN EXPERIENCED CRYSTALLOGRAPHIC REFEREE.

No syntax errors found. CIF dictionary Interpreting this report

Datablock: mwp54_f1

```
Bond precision:  C-C = 0.0019 A          Wavelength=1.54184

Cell:            a=19.0674(2)     b=9.7671(1)         c=8.7924(1)
                 alpha=90         beta=102.721(1)     gamma=90
Temperature:     100 K

                Calculated                Reported
Volume          1597.24(3)                1597.24(3)
Space group     P 21/c                    P 21/c
Hall group      -P 2ybc                   -P 2ybc
Moiety formula  C17 H17 N5 O S            C17 H17 N5 O S
Sum formula     C17 H17 N5 O S            C17 H17 N5 O S
Mr              339.42                    339.41
Dx,g cm-3       1.411                     1.411
Z               4                         4
Mu (mm-1)       1.923                     1.923
F000            712.0                     712.0
F000'           715.22
h,k,lmax        24,12,11                  24,12,10
Nref            3461                      3335
Tmin,Tmax       0.871,0.975               0.765,1.000
Tmin'           0.780

Correction method= # Reported T Limits: Tmin=0.765 Tmax=1.000
AbsCorr = GAUSSIAN

Data completeness= 0.964          Theta(max)= 78.905

R(reflections)= 0.0319( 2985)     wR2(reflections)= 0.0842( 3335)

S = 1.067                  Npar= 220
```

The following ALERTS were generated. Each ALERT has the format
test-name_ALERT_alert-type_alert-level.
Click on the hyperlinks for more details of the test.

Alert level G

```
PLAT910_ALERT_3_G Missing # of FCF Reflection(s) Below Theta(Min).          1 Note
PLAT912_ALERT_4_G Missing # of FCF Reflections Above STh/L=  0.600        114 Note
PLAT978_ALERT_2_G Number C-C Bonds with Positive Residual Density.         10 Info
```

```
   0 ALERT level A = Most likely a serious problem - resolve or explain
   0 ALERT level B = A potentially serious problem, consider carefully
   0 ALERT level C = Check. Ensure it is not caused by an omission or oversight
   3 ALERT level G = General information/check it is not something unexpected

   0 ALERT type 1 CIF construction/syntax error, inconsistent or missing data
   1 ALERT type 2 Indicator that the structure model may be wrong or deficient
   1 ALERT type 3 Indicator that the structure quality may be low
   1 ALERT type 4 Improvement, methodology, query or suggestion
   0 ALERT type 5 Informative message, check
```

It is advisable to attempt to resolve as many as possible of the alerts in all categories. Often the minor alerts point to easily fixed oversights, errors and omissions in your CIF or refinement strategy, so attention to these fine details can be worthwhile. In order to resolve some of the more serious problems it may be necessary to carry out additional measurements or structure refinements. However, the purpose of your study may justify the reported deviations and the more serious of these should normally be commented upon in the discussion or experimental section of a paper or in the "special_details" fields of the CIF. checkCIF was carefully designed to identify outliers and unusual parameters, but every test has its limitations and alerts that are not important in a particular case may appear. Conversely, the absence of alerts does not guarantee there are no aspects of the results needing attention. It is up to the individual to critically assess their own results and, if necessary, seek expert advice.

Publication of your CIF in IUCr journals

A basic structural check has been run on your CIF. These basic checks will be run on all CIFs submitted for publication in IUCr journals (*Acta Crystallographica*, *Journal of Applied Crystallography*, *Journal of Synchrotron Radiation*); however, if you intend to submit to *Acta Crystallographica Section C* or *E* or *IUCrData*, you should make sure that full publication checks are run on the final version of your CIF prior to submission.

Publication of your CIF in other journals

Please refer to the *Notes for Authors* of the relevant journal for any special instructions relating to CIF submission.

PLATON version of 07/08/2019; check.def file version of 30/07/2019

Datablock mwp54_f1 - ellipsoid plot

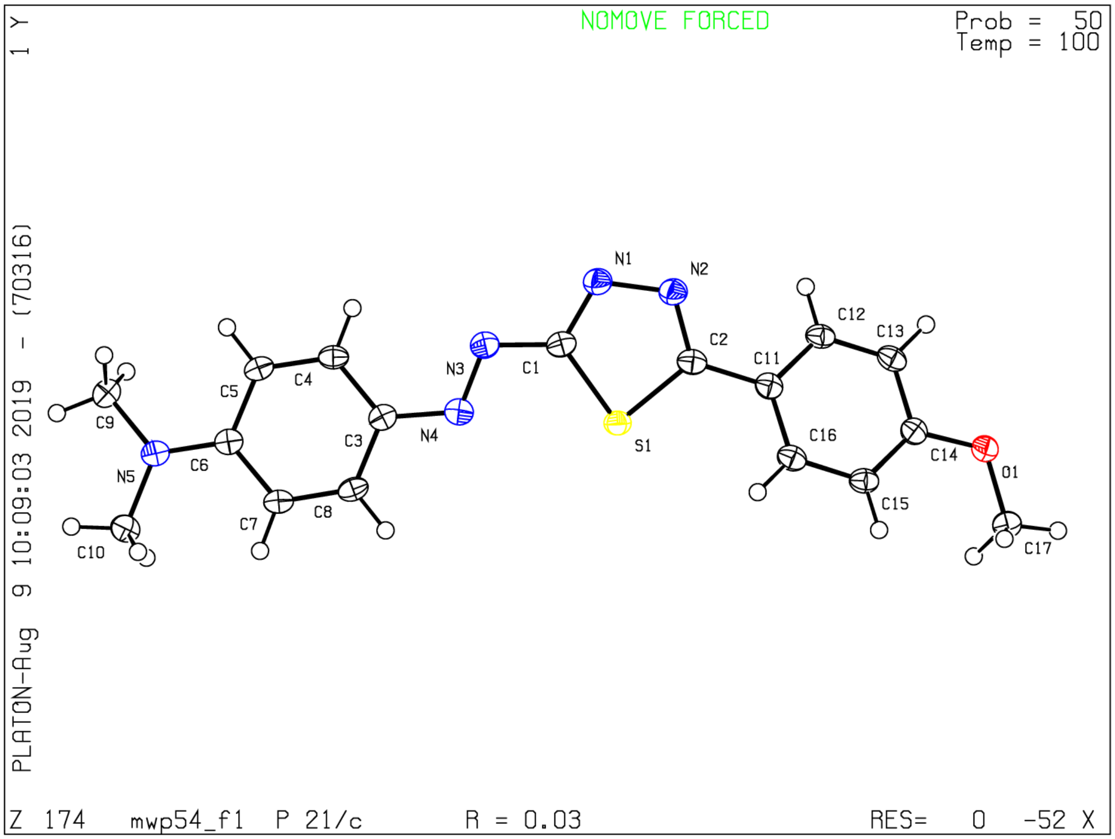

Supplement: Supplementary file 1 [file molecules-25-02822-s001.pdf]
